# Mental health, social determinants and One Health in skin neglected tropical diseases (NTDs): A scoping review

**DOI:** 10.1371/journal.pntd.0014034

**Published:** 2026-08-06

**Authors:** India Hotopf, Idiatou Diallo, Jennifer Ljungqvist, Josefine Tecklenborg, Sascha Knauf, Anna S. Fahrion, Alexandra Juhász, Rosalind McCollum, Gillian Turner, Julian Eaton

**Affiliations:** 1 Department of International Public Health, Liverpool School of Tropical Medicine (LSTM), Pembroke Place, Liverpool, United Kingdom; 2 Department of Global Health and Population, Harvard TH Chan School of Public Health, Boston, Massachusetts, United States of America; 3 Institute of International Animal Health/One Health, Friedrich-Loeffler-Institut, Federal Research Institute for Animal Health, Greifswald - Insel Riems, Germany; 4 Faculty of Veterinary Medicine, Justus Liebig University, Giessen, Germany; 5 Department of Tropical Disease Biology, Liverpool School of Tropical Medicine (LSTM), Pembroke Place, Liverpool, United Kingdom; 6 Department of Clinical Sciences, Liverpool School of Tropical Medicine (LSTM), Pembroke Place, Liverpool, United Kingdom; KEMRI-Wellcome Trust Research Programme: Centre for Geographic Medicine Research Coast, KENYA

## Abstract

**Background:**

Skin NTDs cause mental ill health, primarily through social stigma and discrimination, overburdening services and undermining elimination efforts. A holistic One Health (OH) approach is essential for NTD elimination, but evidence and operational guidance lack. This scoping review documents the current state of integration of mental health and OH-based skin-NTD programmes. It forms part of a series of reviews on the role of OH in skin NTDs, led by the WHO skin NTD working groups.

**Methodology:**

We searched four peer-reviewed databases for terms related to skin NTDs, OH, mental health and social determinants of health (SDH). Peer-reviewed articles exploring all four concepts were eligible, with no language/geographical/time constraints. Grey literature, conference abstracts and non-peer-reviewed materials were excluded. Abstract screening and extraction was supported by ASreview and NotebookLM. Outcomes related to mental health, OH/SDH and skin NTDs were analysed and charted, using a descriptive thematic analysis approach.

**Principal Findings:**

Twenty-two papers were included; predominantly quantitative studies in Africa, focused on cutaneous leishmaniasis (CL), with few intervention studies. Mental health was typically explored as a downstream effect (primarily stigma and distress), with limited use of validated instruments. Few studies meaningfully embedded OH approaches, including cross-sectoral collaboration and changes were rarely attributed to climate change. A theoretical framework for the multi-directional and intersectional relationship between mental health, SDH and OH is presented.

**Conclusions/Significance:**

The underlying mechanisms of mental health remain poorly characterised, with a lack of integrated OH thinking. Exposure to skin NTDs is generally due to lack of agency and choice. Evidence on the role of household economic conditions and climate change in relation to mental health and skin NTDs lacks. Participatory research has a critical role to play in co-designing nuanced OH-informed interventions, but it is critical that we build on identified synergies within existing frameworks, including person-centred care and syndemics.

## Author summary

Skin neglected tropical diseases (NTDs), including diseases such as leprosy, are associated with mental illness, primarily due to social stigma and discrimination. This burdens the health system and undermines control efforts. The WHO’s NTD Roadmap calls for a holistic One Health (OH) approach, which addresses the health of humans, animals and the environment. However, there is a lack of evidence and guidelines on how to implement such programmes. To address this, the WHO Skin NTD Working Groups launched a series of reviews on the role of OH and skin NTDs. This review focuses on mental health and the social environment and aims to document the current state of integration of mental health and OH-based skin NTD programmes. We conducted a search of peer-reviewed literature covering four dimensions: skin NTDs, OH, mental health and social determinants of health (SDH) (e.g., socioeconomic status). We found limited evidence, with very few articles embedding interventions, measuring mental health outcomes and meaningfully embedding OH. There was also a lack of evidence on household economic conditions and climate change, despite their known importance in shaping mental health and skin NTDs. We present a theoretical framework for the multi-directional and intersectional relationship between mental health, SDH and OH – our hope is that it can support future interventions. The social sciences have a critical role to play in co-designing appropriate OH-informed interventions, but it is vital that we align with existing frameworks like person-centred care.

## 1 Introduction

OH is an integrated, unifying approach to balance and optimize the health of people, animals and the environment [1]. The OH concept has gained traction in the last two decades, with the gaps in knowledge and practice highlighted during the COVID-19 pandemic further catalysing investment in understanding and applying OH [1]. In the area of infectious disease, OH has primarily focused on zoonoses and vectors, and how animals intersect with environment and humans. At the same time, there is growing recognition of the importance of mental health in NTD work, with high comorbidity reported leading to a huge burden on services and caregivers, alongside suffering and exclusion of those affected [2,3]. Including depression comorbidity in calculation of burden of disease estimates for cutaneous leishmaniasis, for example, increases estimate ten-fold [4]. This mental health dimension of NTDs is shaped by SDH, which are mediated by the intersection of humans, animals and the environment [5–8]. Hence, the OH approach is considered vital in addressing multidimensional health challenges and achieving the Sustainable Development Goals (SDGs), including goal number three: to ensure healthy lives and promote well-being for all [9]. Although mental health is a key component of OH, a recent scoping review revealed a lack of literature on the relationship between OH and mental health [10].

Globally, 1.7 billion people are impacted by NTDs, mainly in marginalised communities in low- and middle-income countries [11]. In addition to causing lifelong disability, NTDs are associated with mental illness [2]. Whilst some NTDs directly cause mental illness directly, mental health impacts are largely associated with social exclusion and discrimination – which is particularly prominent in skin NTDs [2,12]. Myths, misinformation and exclusion from education and employment typically drive generational poverty and perceptions of persons affected by skin NTDs as individuals who are failing to contribute to society [2,13–15]. Stigma and mental illness are further associated with health-seeking delays and inaction, impeding elimination efforts [2,16,17]. Consequently, the past decade has witnessed a growing interest in the relationship between mental health and NTDs, particularly skin NTDs [18].

Skin NTDs are often caused by zoonotic pathogens and in many cases are linked to environmental factors such as insect vectors or animal disease reservoir systems, which requires evidence-based decision making and cross-sectoral control strategies [1,18]. The World Health Organization (WHO) recognises OH as essential in The NTD Roadmap 2021–2030 targets, but the evidence-base is scarce [1,18,19]. The absence of a unified operational framework, combined with the cross-sectoral and context-specific nature of the OH framework, challenges the implementation of integrated disease control and prevention programmes [20,21]. Yet, existing knowledge gaps in disease ecology [11] present challenges to effective management of skin NTDs. A more holistic approach incorporating social sciences and mental health is essential for sustainable prevention and control, including through people-centred management of mental illness [22].

In response to the evidence gaps and global priority of embedding a OH approach in NTD elimination efforts, a complementing series of scoping reviews on the role of OH set out to inform WHO skin NTD strategies and may provide lessons for other disease programmes wishing to embed OH approaches [18]. This scoping review sought to document the current state of integration of mental health and OH-based skin NTD programmes, through the following objectives:

1. Map the existing body of evidence on the interface between mental health and OH within the context of skin NTDs
2. Outline putative mechanisms and theories of action for poor mental health in persons affected by skin NTDs, through a OH lens
3. Provide evidence-informed recommendations on future action on mental health and skin NTDs incorporating the OH approach

## 2 Methods

A scoping review methodology was used to map the available evidence, covering a range of topics and study designs [23]. The scoping review is presented in line with the Preferred Reporting Items for Systematic Reviews and Meta-Analyses (PRISMA) guidelines [24] and was registered on the Open Science Framework (10.17605/OSF.IO/WQ53M) (please see SI1 for the completed PRISMA scoping review checklist adapted from Rahman, Rajaratnam [19].

### 2.1 Concepts

The search strategy included keywords and Medical Subject Headings (MeSH) terms related to skin-NTDs, OH, mental health and the SDH of mental disorders and SDGs framework from Lund, Brooke-Sumner [6].

### 2.2 Data sources and search strategy

IH and ID developed the search strategy in consultation with technical experts in the WHO skin NTD OH working group, the core research team and the LSTM Academic Liaison and Training Specialist. Terms related to skin NTDs, and OH were gathered from Tecklenborg [25] with some additions. The search strategy was translated into French, Spanish, Portuguese and German and refined and piloted in Pubmed. The finalised search strategy was run on the 30/07/2025 on: Embase, PsychINFO, Web of Science and Pubmed (see SI2 for the full search strategy).

### 2.3 Eligibility criteria

The PCC framework (Population, Concept, Context) shaped our inclusion and exclusion criteria and search strategy development [26]. Our population was persons affected by skin NTDs and articles had to address all four key areas of skin NTDs, OH, mental health and SDH. There were no contextual constraints. Peer-reviewed primary research articles published in all languages were eligible and there was no timeframe constraint. Grey literature, conference abstracts, dissertations, non-peer-reviewed materials and reviews were ineligible.

The main variables of interest were:

1) Mental health outcomes (including psychological well-being, mental illness, stigma, psychosocial distress, and disability)
2) SDH (demographic factors, economic conditions, neighbourhood characteristics, environmental and climate-related exposures, and social and cultural practices and knowledge)
3) OH dimensions (including human-animal-environment interactions, ecological and climate-related factors, and health systems integration). Please note that use of the specific ‘One Health’ term was not a requirement.

### 2.4 Screening and selection

Following the search, all identified citations were collated and uploaded into EndNote [27], with duplicates removed, before exporting citations as.txt format. Titles and abstracts were screened using ASreview version 1.6.5 [28] which supports researcher-in-the-loop screening prioritisation using active learning models [28], to align with the methods of Tecklenborg [25]. The algorithm ranks citations from most to least relevant, presenting the most relevant citation to the reviewer first, iteratively learning as you label citations as relevant and irrelevant.

IH and ID independently double screened the first 20% and compared agreement, achieving >90% agreement [29]. Remaining citations were shared and screened independently. Batches included a selection of citations deemed as relevant/irrelevant from the 20% screening. Before beginning the second batch of screening, these citations were labelled as either irrelevant or relevant using the ‘prior knowledge’ setting. This trained the algorithm, improving its ability to identify the most relevant citations [30].

### 2.5 Data extraction and synthesis

IH and ID developed an extraction tool in Excel, based on the study outcomes and the literature. Once the extraction tool was finalised, the tool was fed into Chat GPT-4o to convert each piece of information into a question [31]. The NotebookLM [32] question sheet was piloted and amended, using chat GPT to gather suggestions on appropriate edits and amendments to improve the accuracy and quality of form. This included adding a command that NotebookLM provide all verbatim quotes alongside summaries, to support quality checking (see SI3 for data extraction question list). The prompts were also iteratively amended throughout the extraction process. Where papers were in a language not spoken by the screener, translation software DeepL [33]. Note that all included articles were in English and did not require translation.

Once tools were piloted and finalised, relevant citations were split among IH, ID and JL for full text screening and extraction, using NotebookLM. The process included reading papers in full and carefully checking the accuracy of the summaries and quotes, making careful iterations to the prompts and triangulating by reviewing sections of the papers, to ensure quality – known as a “human-in-the-loop approach” [34,35]. The input data provided for NotebookLM consisted of the question sheet (with prompts for each piece of information) and PDF of the article being analysed (please see SL4 for the extraction guide used).

Afterwards, relevant sections of summaries were pasted into the extraction table, along with any additional text required. To promote consistency, extractors met regularly to clarify uncertainties and align interpretation. Once the results were drafted, IH cross-checked all papers and found a limited number of omissions that were not captured initially. Following data extraction, study findings were manually analysed and charted, using a descriptive thematic analysis approach. Findings were synthesised and presented by IH, ID and JL according to the three domains of mental health, OH/SDH.

As this was a scoping review, no participant-level data was analysed and no meta-analyses were conducted. Where papers had incomplete reporting of certain variables (e.g., sample size), relevant fields were recorded as ‘not reported’. Descriptive summaries were based on the information available in the included studies, with no imputations of missing data performed.

## 3 Results

### 3.1 Study characteristics

**Fig 1:**
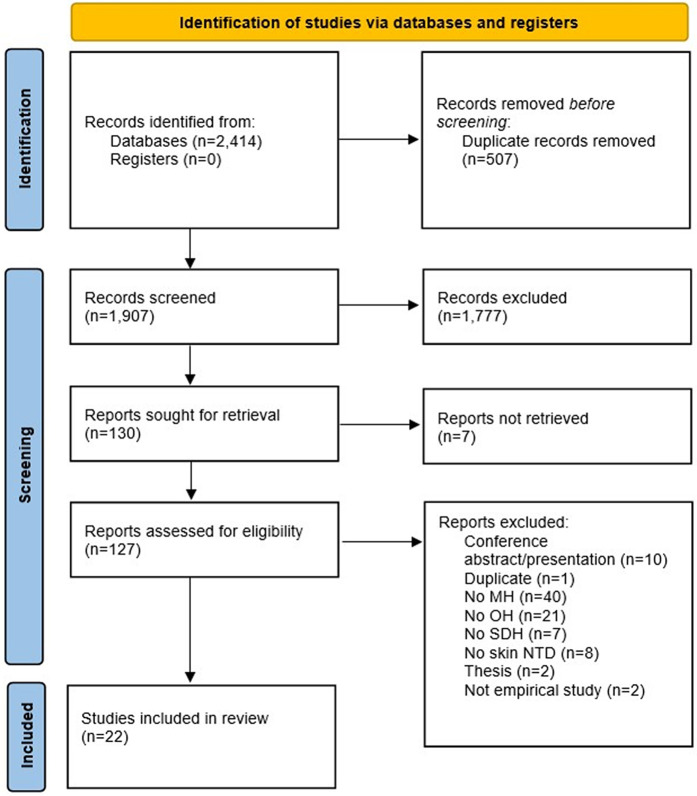
PRISMA flow diagram.

Our searches identified 2414 articles for potential inclusion, with 22 deemed eligible (Fig 1). Please see SI4 for the complete screening and extraction table.

Articles predominantly focused on a single disease (n = 19), with cutaneous leishmaniasis (CL) (n = 9), lymphatic filariasis (LF) (n = 4) and tungiasis (n = 3) most common. Most articles focused on a single country (n = 19) and were conducted in Africa (n = 10), followed by Asia (n = 6), with Uganda (n = 2), Cameroon (n = 2) and Indonesia (n = 2) most common (Table 1). Only one study was conducted in high income country (Taiwan). Most research was conducted at the community level, and participants were typically persons affected by skin NTDs only (i.e., Persons with clinical diagnoses) (n = 13) or the general population of settings where research was conducted, including persons affected by skin NTDs (n = 6).

**Table 1:** Characteristics of included studies.

Author (year)	Title	Country	Quant/qual/mixed	Study design	Participants	Sample size	Disease	Setting	Population	Framework	Aim	Outcome measure	Intervention	Risk factors
Asiedu, Kwarteng [36]	Financial burden impact quality of life among lymphatic Filariasis patients	Ghana	Quantitative	Cross-sectional	Patients	155	LF	Community	Patients	Quality of Life	Assess the overall quality of life of LF pathology patients in some selected endemic communities in rural Ghana (hotspots)	Quality of Life - Lymphatic Filariasis Quality of Life Questionnaire (LFSQQ)	None	Built environment and infrastructure Human-animal interaction Demographics Economic factors
Bamorovat, Sharifi [37]	A prospective longitudinal study on the elimination trend of rural cutaneous leishmaniasis in southeastern Iran: Climate change, population displacement, and agricultural transition from 1991 to 2021	Iran	Mixed methods	Longitudinal	Patients	N/A	CL (ACL and ZCL)	Health system (clinic, health centre, network)	Patients	None	To examine the trends in the epidemiology of CL over 30 years in Bam County. It explored its relationship with the increasing urbanization and changes in the climate, environment, and agricultural practice.	Disease incidence	None - conducted within context of sustained control measures and agrarian reform policies	Land use changes Climate change Built environment and infrastructure Demographics Conflict and migration
Bantie, Kassaw [38]	Magnitude and associated factors of cutaneous leishmaniasis among patients visiting Nefas Mewcha primary hospital, Northern Ethiopia, 2022: An institution-based Cross-sectional study	Ethiopia	Quantitative	Cross sectional	People visiting hospital (including NTD patients)	332	CL (LCL and MCL)	Hospital	People visiting hospital (including NTD patients)	None	To investigate the magnitude, clinical presentation and associated factors of CL among patients visiting NMPH in 2022.	Disease burden (magnitude/prevalence)	None	Built environment and infrastructure Human-animal interaction Education and awareness Demographics Economic factors
Brieger, Oshiname [39]	Stigma associated with onchocercal skin disease among those affected near the Ofiki and Oyan Rivers in western Nigeria	Nigeria	Mixed methods	Pre and post (linked to RCT)	Inhabitants (5 + years)	1032	Onchocerciasis (Onchocercal Skin Disease (OSD)	Community	Inhabitants (5 + years)	Westbrook et al. (1992) stigma framework	To present the baseline perceptions of stigma among participants affected by OSD in the Ifeloju LGA.	Self-perceived social stigma	The study gathered baseline data for a multi-country double blind, placebo controlled trial testing the effect of ivermectin on OSD and severe itching. The trial tested 3-month, 6-month, and 12-month treatment regimens.	Built environment and infrastructure Cultural practices and beliefs Demographics Economic factors
Chahed, Bellali [40]	Psychological and Psychosocial Consequences of Zoonotic Cutaneous Leishmaniasis among Women in Tunisia: Preliminary Findings from an Exploratory Study	Tunisia	Quantitative	Cross sectional	Women affected	500	CL (ZCL).	Community and health system (clinics)	Women affected	Self-regulatory theory framework by Leventhal, Meyer & Neren	To examine the psychosocial impact of ZCL scars among Tunisian women.	Psychosocial impact: illness perception (IPQ-R scores), psychosocial adjustment to stress/stigma (PLSI scores), and quality of life (WHOQOL-26 scores).	None	Seasonality Climate change Human-animal interaction Education and awareness Demographics
Deribe, Simpson [41]	Predicting the environmental suitability and population at risk of podoconiosis in Africa	29 African countries	Quantitative	Secondary analysis (modelling)	General population	N/A	Podoconiosis	Unspecified	General population	None	To i) map the geographical distribution of podoconiosis in Africa, ii) estimate the population at risk of podoconiosis, iii) determine the number of WHO implementation units (IUs) where mapping is required, and iv) delineate overlapping risk of podoconiosis and LF.	Estimation of the total human population at risk in areas environmentally suitable for podconiosis	None	Seasonality Economic factors
Garchitorena, Sokolow [42]	Disease ecology, health and the environment: a framework to account for ecological and socio-economic drivers in the control of neglected tropical diseases	Cameroon and Benin, Senegal	Quantitative	Secondary analysis (modelling)	Inhabitants, families, community leaders, school teachers, HWs, MSF personell and local authorities	Not reported	BU and schistosomiasis	Unspecified	Persons affected	system dynamic; coupled economic–epidemiological systems framework; planetary health	To describe a simple framework for studying the complex relationships between poverty, infectious diseases, and the environment. The aim of this framework is to help investigate environmental drivers of joint poverty–disease dynamics, with the ultimate goal of identifying sustainable solutions for human and planetary health	N/A – conceptual/theoretical paper	None - conducted within the context of national MDA programme	Seasonality
Giles-Vernick, Owona-Ntsama [43]	The puzzle of Buruli ulcer transmission, ethno-ecological history and the end of “love” in the Akonolinga district, Cameroon	Cameroon	Qualitative	Descriptive (Ethnographic)	Patients	52	BU	Community	Inhabitants, families, community leaders, school teachers, HWs, MSF personell and local authorities	One World One Health Initiative; ‘participatory model’ approach Leach and Scoones (2013)	To build on debates about the contributions of “local knowledge” and “alternative models” to biomedical knowledge of disease transmission.	Emergent themes/findings regarding historical, ecological, agricultural, and social changes associated with Buruli ulcer transmission	None	Land use changes Education and awareness Cultural practices and beliefs Demographics Economic factors
Hamouchi, Daoui [44]	Epidemiological features of a recent zoonotic cutaneous leishmaniasis outbreak in Zagora province, southern Morocco	Morocco	Quantitative	Descriptive	Persons affected	114	CL (ZCL)	Community	Patients	None	To understand the epidemiological characteristics of the recent outbreak and to shed light on the features of the ZCL outbreak in order to enable more focused and engaged control procedures to prevent new outbreaks in the future.	Epidemiological features of the outbreak (total number, geographical spread, and age/sex distribution) and the molecular confirmation and identification of the causative Leishmania species.	None	Seasonality Built environment and infrastructure Education and awareness Demographics
Jamaluddin [45]	The hygiene hypothesis of crime: Examining the link between disgust-related diseases and crime incidence	Indonesia	Quantitative	Secondary analysis (modelling)	Persons affected	N/A	Leprosy and LF	Unspecified	Persons affected	Pathogen Avoidance Theory and the Behavioral Immune System.	To examine the relationship between disgust-related diseases (diarrhea, HIV, and leprosy) and violent crimes (sex- related crimes, assault, and homicide) in Indonesia	Overall Crime Index and specific categories of violent crimes (sex-related crimes, assault, and homicide).	None	Cultural practices and beliefs
Krentel and Aunger [46]	Causal chain mapping: A novel method to analyse treatment compliance decisions relating to lymphatic filariasis elimination in Alor, Indonesia	Indonesia	Qualitative	Descriptive	Persons affected (compliars and non compliars)	43	LF	Community	Persons affected (compliars and non compliars)	Semantic Network Analysis; health promotionn models (The Health Belief Model [Strecher and Rosenstock 1997], Theory of Planned Behaviour [Ajzen and Fishbein 1980] and Social Cognitive Theory [Bandura 1986])	To present a formal method for analysing relevant factors in the situational context of the compliant behaviour, identifying how these factors may interact within the individual	DALYs attributable to environmentally mediated infectious diseases	None - conducted within the context of national MDA programme	Education and awareness Demographics Economic factors
Lin, Chang [47]	Increased risk of bipolar disorder in patients with scabies: A nationwide population-d-cohort study	Taiwan	Quantitative	Cohort study	Patients	35471	Scabies	Nationwide	Patients	None	To investigate the possible relationship between scabies and BPD	Incidence rate of scabies and bipolar disorder	None	Demographics
Lu, Khan [48]	Epidemiology of cutaneous leishmaniasis in children of Khyber Pakhtunkhwa, Pakistan	Pakistan	Quantitative	Secondary analysis (modelling)/cross sectional	Patients and refugees	1011	CL	Community and health system	Patients and refugees	None	To scrutinise the prevalence, spatial distribution and socio-demographic and behavioural risk factors associated with cutaneous leishmaniasis in children aged <5–15 years in Khyber Pakhtunkhwa.	Prevalence, spatial distribution and risk factors associated with cutaneous leishmaniasis	None	“Seasonality Built environment and infrastructure Human-animal interaction Education and awareness Demographics Economic factors“
McNeilly, Mutebi [49]	Reduction of tungiasis prevalence, intensity, and morbidity during a two-year long community-based tungiasis control project in a hyperendemic region in Karamoja, Uganda	Uganda	Quantitative	Pre and post implementation study	Household caretakers	1329	Tungiasis	Community	Inhabitants	One Health	To investigates tungiasis-related stigma and control practices in the impoverished Napak District in rural northeastern Uganda, where tungiasis is hyperendemic and effective treatment is unavailable	Prevalence of tungiasis, the prevalence of embarrassment, and the quantification of judging attitudes and control practices.	Endline data collection for a two-year community-based One Health intervention combined quarterly detection and immediate NYDA treatment of human and animal tungiasis with continuous health education. Sixteen community dialogue meetings—attended by over 803 adults—provided feedback opportunities, addressed local needs, and delivered information on tungiasis prevention and care.	Built environment and infrastructure Human-animal interaction Education and awareness Cultural practices and beliefs Demographics Economic factors Conflict and migration
McNeilly, Thielecke [50]	Tungiasis Stigma and Control Practices in a Hyperendemic Region in Northeastern Uganda	Uganda	Quantitative	Cross sectional (baseline for intervention)	Inhabitants	6558	Tungiasis	Community	Household caretakers	Stigma Theory; Bio-Social approach	To evaluate a two-year long humanitarian One Health tungiasis control project in 17 villages in Napak district, Karamoja region, Northeastern Uganda	Tungiasis prevalence among residents in the study area	Baseline data collection for a two-year long tungiasis control intervention project (2021/2022), which includes regular screening of the population for tungiasis; treatment of cases with a mixture of two dimeticone oils (NYDA); and tungiasis-related health education and community engagement.	Built environment and infrastructure Education and awareness Cultural practices and beliefs Demographics Economic factors
Mørkve ÅW. et al (2023)	A qualitative case study of community experiences with Tungiasis in high prevalence villages of Bungoma County, Kenya: “The whole body aches and the jiggers are torturing me!”	Kenya	Qualitative	Participatory action research	Infected children and adults, teachers and pupils, public health officers, community health workers and NGO volunteers” families who participated in program	48	Tungiasis	Community and health system (programme)	Infected children and adults, teachers and pupils, public health officers, community health workers and NGO volunteers	Kleinman‘s explanatory model of health and illness (Kleinman, Eisenberg, and Good, 1978); Dialogic epistemology	To contribute with knowledge about the experiences of those affected, perceived causes and local coping strategies, to improve the control and elimination of this neglected condition.	Themes related to Suffering the condition, Causal explanations of the jigger plague and The creation of hopelessness”	Conducted within context of Red Cross’ Jigger removal program - some collaboration with Red cross and previous links	Built environment and infrastructure Human-animal interaction Education and awareness Demographics Economic factors
Oliveira, Galati [51]	Neurological disability in leprosy: incidence and gender association in Sergipe, Brazil	Brazil	Quantitative	Secondary analysis	Patients	N/A	Leprosy and LF	Unspecified	Patients	None	To assess the detection rate of new cases, the geographical distribution and association with gender and clinical forms in Sergipe.	New case detection rate/incidence, geographical distribution, disease classification (MB/PB), and neurological disability grades	None	Built environment and infrastructure Demographics Economic factors
Pinto-García [52]	Military Dogs and Their Soldier Companions: The More-than-human Biopolitics of Leishmaniasis in Conflict-torn Colombia	Colombia	Qualitative	Descriptive (Ethnographic)	Soldiers and military vets	Not reported	CL	Military facilities	Soldiers and military vets	More-than-human biopolitics; trans-biopolitics; Affect Theory	To draws on ethnographic field research with human and canine members of the Colombian military	Themes related to war, human–dog affective relationships and biopolitically configured interspecies hierarchies	None - conducted within the context of the Colombian military/MoH’s policies	Land use changes Built environment and infrastructure Demographics Economic factors Conflict and migration
Ramdas [53]	Cruel disease, cruel medicine: Self-treatment of cutaneous leishmaniasis with harmful chemical substances in Suriname	Suriname	Mixed methods	Descriptive (Ethnographic)	Patients	205	CL	Community and health system	Patients	Associative reasoning	To explore why are potentially harmful, non-biomedical chemical substances, such as battery acid, chlorine, herbicides, and insecticides, used in the treatment of cutaneous leishmaniasis (CL) and what drives people to use these products as medicine	Themes related to social and cultural aspects of CL perceptions and treatment	None - conducted within the context of the national control programme for CL	Built environment and infrastructure Education and awareness Cultural practices and beliefs Economic factors
Rauyajin, Kamthornwachara [54]	Socio-cultural and behavioural aspects of mosquito-borne lymphatic filariasis in Thailand: A qualitative analysis	Thailand	Qualitative	Descriptive	Inhabitants (inc persons affected), community leaders	35	LF	Community	Inhabitants (inc persons affected), community leaders	Broadly refers to behavioural frameworks from Mak (1986) and Dunn (1976)	To examine the contribution of socio-cultural and behavioural factors in mosquito-borne lymphatic filariasis transmission in Southern Thailand	Themes related to socio-cultural and behavioural factors in mosquito-borne lymphatic filariasis transmission	None	Education and awareness Cultural practices and beliefs Demographics Economic factors
Stewart and Brieger [55]	Community views on cutaneous leishmaniasis in istalif, afghanistan: Implications for treatment and prevention	Afghanistan	Qualitative	Participatory	Inhabitants	64	CL	Community	Inhabitants	Three-delays model for analyzing treatment-seeking behavior PRECEDE Framework for guiding the educational diagnosis and understanding results. SEED-SCALE model in underlying program structure for participatory community development	To investigate illness recognition and treatment-seeking behavior, beliefs about etiology and transmission of the disease, and views of prevention and protection among the population of Istalif, a district in the Kabul Province.	Themes related to knowledge, attitudes, and preferences regarding CL treatment and prevention	None - conducted within the context of the national control programme for CL	Climate change Built environment and infrastructure Education and awareness Demographics Economic factors Conflict and migration
Tarnas, Abbara [56]	Ecological study measuring the association between conflict, environmental factors, and annual global cutaneous and mucocutaneous leishmaniasis incidence (2005–2022)	52 nations - Africa (n = 11) and the Middle East (n = 10), though the remainder of nations had a wide geographic spread between South America (n = 8), Central America (n = 7), Europe (n = 6), Central Asia (n = 5), South Asia (n = 4), and East Asia (n = 1)	Quantitative	Secondary analysis (modelling)	Persons affected, with focus on IDPs	N/A	CL and ML	Conflict affected settings	Persons affected, with focus on IDPs	None	To measure the associations between annual CL/ML cases, conflict intensity, and environmental factors between 2005 and 2022 globally	Nation-level CL and ML case counts between 2005–2022	None	Seasonality Economic factors Conflict and migration

Only four articles included interventions, with two linked to the same study, namely: a two-year humanitarian OH tungiasis intervention in Uganda [49,50]; a baseline stigma assessment in Nigeria for a multi-country onchocerciasis ivermectin RCT [39] and a situational analysis for the co-design of a community-based leishmaniasis intervention in Afghanistan [55]. More commonly, articles were framed within contexts of national control efforts (n = 5) or wider policies (n = 1).

Thirteen studies used quantitative methods and six used qualitative methods with a wide variety of study designs. Secondary analysis (modelling) (n = 6), descriptive qualitative and cross sectional (n = 4) designs were most common. However, just eight articles prioritised participation and inclusion of persons affected/communities, including through ethnographic approaches (n = 3), engaging key community stakeholders, e.g., leaders and pluralistic providers (n = 2) and using participatory approaches (n = 2).

Where reported (n = 15), sample size ranged from 35 to 35,471, with a median sample size of 205. Most articles incorporated theoretical framework(s) (n = 16), including behavioural/cognitive (n = 5) and systems and ecological/planetary (n = 4) frameworks. Overall, limitations were described in 20 papers and were primarily linked to data and analysis (e.g., inaccurate available data, changes in active case finding, short-term follow-up, small sample size) and generalizability and bias (e.g., sampling bias, social desirability bias, cross sectional design).

### 3.2 Theoretical mechanisms of action

**Fig 2:**
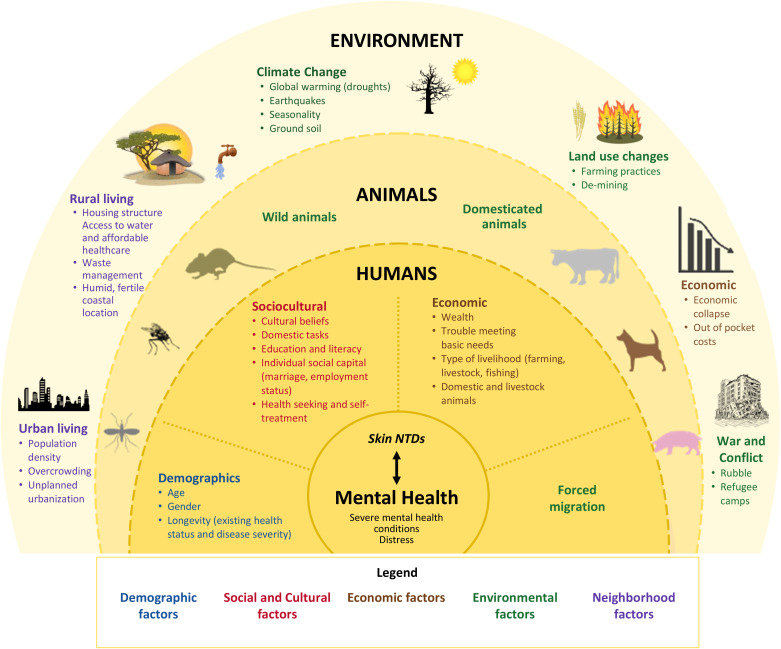
Mechanism of action for the relationship between skin NTDs, mental health and OH, based on the Lund, Brooke-Sumner [6] SDH of mental disorders and SDGs framework (Source: authors own, created on Canva, using images from Openclipart. License: Creative Commons Zero 1.0 (CC0)).

Whilst some articles presented diagrams summarising risk factors for experiencing skin NTDs [37,42,46,48] and poor mental health [57], none of the articles explicitly applied a OH framework in considering the impact of NTDs on human mental health, for example through planetary/environmental factors or the relevance of diseases having zoonotic pathway. Below, we present a summary of the findings of this scoping review using a OH approach (Fig 2). The diagram incorporates the Lund, Brooke-Sumner [6] SDH of mental disorder dimensions with the three OH dimensions of environment, animals and humans.

The figure illustrates the multi-directional and intersectional relationship between mental health, SDH and the OH dimensions of animals, humans and the environment. Within the environment, housing, seasonality, climate change, land use changes, conflict and wider socioeconomic contexts influence the presence and interaction with vectors, as well as access to essential services. Thus, shaping the incidence and management of skin NTDs, including mental health morbidity.

Similarly, the presence and interaction with animal reservoirs, both in the domestic and occupational setting, are shaped by environmental factors, as well as the human dimension, including economic/livelihood and sociocultural determinants.

Populations with certain livelihoods (e.g., farming and fishing) and identity factors (e.g., age and gender) experience disproportionate exposure to skin NTDs and barriers accessing effective treatment. The continuum from distress to mental health conditions is influenced by gender norms, cultural beliefs (e.g., spiritual beliefs), knowledge and awareness, as well as individual social capital and financial means. Together, these factors shape health seeking and self-treatment behaviour, social vulnerability and resilience to the mental impacts of skin NTDs.

### 3.3 Mental health

Below, we summarise the measurement and characterisation of mental ill health in persons affected by skin NTDs within the included articles. Most articles (n = 14) described mental health as a downstream effect of physical morbidity, rather than measuring the construct directly, by referencing emotional suffering and social stigma without assessment [37,38,41–46,48,49,54–56,51]. Where measured (n = 9), approaches included qualitative exploration of psychosocial experiences, such as stigma and stress [52,53,58]. Others used non-structured questionnaires exploring perceptions, worry or self-reported emotional burden [50,53] and use of clinical data to assess the risk of bipolar disorder and scabies [47]. Four articles used validated instruments, including the Lymphatic Filariasis Quality of Life Questionnaire (LFSQQ) [36], adapted Onchocercal Skin Disease (OSD) 13-item stigma scale [39], WHO Quality of Life brief (26-item) questionnaire (WHOQOL-BREF) [40], Revised Illness Perception Questionnaire (IPQ-R) and the Psoriasis Life Stress Inventory [40].

#### 3.3.1 Psychological experiences.

Stigma, including enacted, internalised and anticipated, was the most common construct across skin NTDs (n = 17) and geographical location, and was mediated by gender and physical morbidity (see SDH) [36–44,48–50,52–56]. Psychological including emotional distress (n = 16) was often linked to chronic symptoms, visible disfigurement, impaired mobility and social exclusion [36,37,40–45,48,49,51–55,57]. Quality of life (QoL) was explicitly measured/described in five articles in Iran, Tunisia, Thailand, Ghana and within a 29 country study, and was interlinked with livelihoods (see SDH) [36,37,40,41,54], while depression and anxiety were each referenced in four [37,38,40,41]. Social isolation and disability-linked psychological morbidity (n = 6) across Africa and Asian countries [36,41,49,54,55,59] and stress, including emotional strain and life stressors (n = 3) [36,40,41] were also explored, with one study exploring bipolar disorder [47]. However, interventions rarely addressed MH, with most focusing on detection, prevention, or morbidity management for physical symptoms [39,42,46,49], and were included in two studies in Tunisia and Ghana, mental health was only a secondary outcome [36,40]. No study evaluated interventions specifically targeting psychological distress, stigma reduction, or psychosocial support.

Stigma was pervasive across conditions; CL facial scarring/lesions resulted in low self-esteem of children, marriage exclusion, community rejection and household-level isolation, particularly for young women across Iran, Morocco, Ethiopia, Pakistan and Afghanistan [37,38,44,48,55]. Tungiasis was associated with widespread judgemental attitudes and school-based stigma [50,57]. Similarly, job loss, disrupted schooling and reduced social participation were commonly attributed to BU stigma in Cameroon, Benin, Senegal and Kenya [42,43,57]. LF stigma included physical avoidance, exclusion from meals and marriage, perpetuating social withdrawal and concealment in Thailand [54]. Three quantitative studies linked stigma and disease severity to poorer psychosocial functioning and QoL [36,39,40].

Psychological distress (e.g., depression, anxiety, fear, and emotional exhaustion), was commonly reported across skin NTDs, particularly in CL [37,38] and podoconiosis [41]. The psychological domain was one of the lowest scoring sections for LF-affected populations in two studies in Tunisia and Ghana [36,40], highlighting negative impacts on QoL. Though no cause was postulated and other studies do not replicate this finding, scabies was associated with psychiatric morbidity, including elevated risk of bipolar disorder, in Taiwan [47]. Anxiety was also linked to fear of death and weight loss in those affected by tungiasis, illustrating the “physically and socially existential threat” [50].

### 3.4 One Health

Below, we consider the OH dimensions of skin NTDs in relation to mental illness. Note that throughout, the underlying mechanism of mental illness and OH determinants are scarcely discussed by authors explicitly. More commonly, implications for skin NTD prevalence and incidence and changes in SDH, which we know to have downstream effects on mental health, are considered. Therefore, our interpretation presented below draws on OH synthesis to consider how reported changes in SDH may plausibly relate to mental health outcomes, even when this linkage is not made explicit by authors.

Only three articles explored all three sectors: humans, animals and the environment [40,48,49] and just three used the term ‘One Health’ [43,49,57]. Although OH relates to the intersection of the three sectors, we summarise findings of each separately, before considering the degree of interdependencies and cross-sectoral collaboration inferred from the papers – a core feature of OH approaches [60]. Note that within the ‘environment’ and ‘human’ sections, findings including disease experience are structured by SDH.

#### 3.4.1 Environment.

Thirteen (n = 13) articles explored environmental determinants of disease transmission and livelihoods, including seasonality, land use and climate change, which we infer may have downstream effects on mental health and disability [37,39–44,48,52–56]. Seven articles further linked built environments/infrastructure (the ‘neighbourhood’ SDH) to skin NTDs.

Regarding seasonality (n = 6), studies on CL/ZCL outbreaks revealed ecological cascades, where rainfall promotes vegetation, stagnant water, and proliferation of vectors and rodents, as detailed by authors [40,44,48,56]. In Pakistan, summer transmission (July–September) produced peaks in CL cases (infected persons visiting hospitals) between December–March [48]. A 52-country study demonstrated optimal risk at 17–24˚C and a negative association between CL/ML cases and humidity [56]. Podoconiosis risk was linked to precipitation (1,000–1,800 mm), elevation (1,000–2,500 *m* above sea level), and acidic, clay- or silt-rich soils across 12 African countries, with the authors concluding that 114.5 million people are consequently at risk of podoconiosis, which we infer may lead to mental health impacts [41]. Another study reported that BU risk increased with intense rainfall, low water flow and water oxygenation level, mildly acidic pH, and high temperatures, as well as stagnant water [42].

Land use changes (n = 3) influenced skin NTDs across contexts, as reported by authors. In Colombia, de-mining in rainforests united soldiers, dogs, and sandflies, increasing CL risk, whereby dogs that were central for survival (land-mine detection) acted as potential reservoirs [52]. In Southeastern Iran, drought and agrarian reforms reportedly resulted in a shift to less water-intensive crops such as palm tree gardens which significantly excluded gerbils hosting ZCL, thus reducing ZCL prevalence, but enabling anthroponotic CL re-emergence, subsequently driving mental illness [37]. In Cameroon, the shift away from colonial-era plantation crops of cocoa and coffee toward food crops (e.g., melon seeds) and escalation of swidden agriculture (clearing of flooded forests), alongside climate-driven fires transformed forests, reportedly altered nutrition (disappearance of fish and game), and promoted human exposure to *Mycobacterium ulcerans* [43].

Some environmental changes were attributed to climate change, with Stewart and Brieger [55] noting the role of global warming and another study finding that 66% of affected Tunisian women linked ZCL, which we know is associated with profound mental health impacts, to extreme, human-driven temperature changes [40]. Droughts and earthquakes were also considered key factors in shaping the re-emergence of CL in Iran [37]. Livelihoods and changes therein were also closely linked to land use and environment changes; these are explored in section 3.4.3 below.

*Built environment and infrastructure*

Thirteen (n = 13) papers explored the role of the built environment and infrastructure [36–39,44,48–50,52,53,55,57,61]. Housing materials and the residential setting in largely rural settings was linked to skin NTD transmission by authors, which is likely driving disability and mental illness. CL was associated with rural living [38,44,52] and widespread unplanned urbanization in Iran [37]. Mud or cattle-dung houses and settlements near agricultural or forested areas and refugee camps, increased risk of CL in Pakistan [48], whilst rubble from conflict heightened sandfly exposure in Afghanistan [55], and remote hinterland living in Suriname reportedly constrained access to formal healthcare [53]. Limited use of bed nets also drove sandfly exposure as reported by a few studies [38,48,55].

Similarly, mud or sandy houses, dirty floors and poor waste management, as well as inadequate water access, was reportedly associated with increased tungiasis [49,50,57]. Humid, fertile, coastal areas and proximity to rivers were linked to higher LF and OSD risk [39], whilst inadequate access to water also impeded skin NTD prevention and self-care practices, influencing disease morbidity [36,50]. In Brazil, five- and nine-person households were reportedly associated with more leprosy cases [51].

#### 3.4.2 Animals.

Six (n = 6) studies explored human-animal interactions [36,38,40,48,49,57]. Livelihoods heightened interactions; most LF-infected people in Ghana engaged in agrarian/fishing activities (62.58%) and Tunisian respondents linked animal-related work and associated environments to increased ZCL prevalence, which caused a range of psychological and psychosocial impacts in persons affected [36,40]. Four articles linked domestic animal proximity to infection risk: Kenyan households commonly slept alongside them with little awareness of reservoir roles [57], living with dogs/livestock increased CL risk (AOR: 5.29) in Ethiopia [38], and similar CL associations were found in Pakistan [48]. See Tecklenborg [25] for further associations. In Uganda, an OH intervention reduced animal tungiasis from 14.2% to 2%, with pigs identified as key reservoirs [49]. Environmental measures such as smearing earthen floors with mud and hygiene measures were also promoted to interrupt the off-host cycle in Northeastern Uganda [49].

#### 3.4.3 Humans.

All articles explored the human dimension, with varying focus on interlinked social and cultural (n = 13), demographic (n = 18) and economic (n = 16) factors, as well as conflict and migration (n = 5).

*Social and Cultural Factors*

Sociocultural factors largely focused on education and awareness (n = 12) and cultural practices and beliefs (n = 7), which shaped experiences of stigma and health seeking behaviour, in particular.

Eight articles observed low BU, tungiasis, CL and LF awareness [40,43,49,50,53–55,57]. CL risk was also associated with low education in Ethiopia and Kenya [38,50], with most CL-affected men reportedly having low or no education in Suriname [53]. In Afghanistan [55], few study participants linked sandflies to CL/ZCL which impeded understanding and in Tunisia [40], respondents recognised vectors but seldom linked their removal to prevention. Limited awareness reportedly further influenced health-seeking behaviour. So-called ‘wild west stories’ citing painful treatment deterred formal healthcare-seeking in Suriname [53], and self-medication complicated reportedly ZCL management in Morocco [44]. In Ethiopia, poor CL knowledge increased risk fourfold and correlated with low bed-net use [38].

Consequently, several articles highlighted the value of awareness raising and community sensitisation [38,48–50]. Community health prevention measures reduced the prevalence of tungiasis in Uganda. However, some authors emphasised that knowledge does not necessarily translate to behaviour change, with greater ZCL awareness driving hopelessness in Tunisia [40]. Instead, personal beliefs, social norms and family members’ fears of side-effects reportedly shaped compliance for LF medication [46]. Chahed, Bellali [40] found that higher knowledge of ZCL was correlated with a lower likelihood of women and girls with ZCL scars viewing their quality of social life in a negative manner yet simultaneously linked higher knowledge to stronger emotional reactions and perceptions of consequences.

Regarding cultural practices and beliefs, seven articles highlighted the role of myths and spiritual beliefs in shaping experiences of skin NTDs [39,43,45,49,50,53,54]. Articles commonly highlighted misconceptions of skin NTDs being self-afflicted and caused by “spells” on “nasty” [43], “dirty”, “lazy”, or “irresponsible” people [50], as well as causing fertility issues. One study also found a positive correlation between disgust-related diseases (i.e., leprosy) and crime rates [45]. Ultimately, these beliefs appear to drive social stigma and impede individuals’ social capital, by thwarting marriage, employment and education prospects, as discussed in section 3.1.

Sociocultural beliefs, combined with experiences of stigma, also seem to contribute to harmful self-treatment practices, such as using chemicals, kerosene, tobacco or motor oil [49,50,53], extracting sandflies using sharp tools [49,50] or rubbing ants on inflamed lymph nodes [54], for tungiasis, CL and LF. In Suriname, the use of dangerous self-treatment remedies was reportedly perpetuated by the belief that “cruel diseases need cruel cures” [53]. Additionally, in Thailand, a study found that strong spiritual beliefs and perceptions that biomedicines failed to address the cause or that disease symptoms were normal or self-resolving, resulted in the use of “folk healing methods” or healthcare delay among persons affected by LF [54]. However, in a Uganda study, just 25/1119 people affected by tungiasis reported seeking herbal remedies [50].

*Demographics and health status*

Most studies focused on associations between demographic traits, predominantly age and gender, personal health status and mental health. Only two studies considered the role of existing health status. An Indonesian study found that milder LF symptoms reduced healthcare-seeking behaviour [46]. The other study, a nationwide population-based matched-cohort study in Taiwan, found that the scabies affected cohort had a higher risk of subsequent bipolar disorder than the matched control group, with a crude hazard ratio of 1.86 (95% CI, 1.36–2.54, P < 0.001) [47].

Age was often linked to disease prevalence, rather than mental health outcomes. Six studies reported a higher prevalence of tungiasis [49,50,57], LF [54] and CL in children, with the latter attributed to domestic activities and playing outside [44,48]. One study found scabies was higher in both the young and elderly [47]. Others found a higher CL prevalence among young adults [38,40], and LF prevalence among middle-aged adults [46]. Stigma also reportedly declined with age in Western Nigeria [39].

Gendered dimensions were explored in 16 articles and were generally shaped by disease condition, exposure and social context [36–40,43,44,46,48–50,52–55,61]. Some articles (n = 5) highlighted male vulnerability to skin NTDs; outdoor work/play increased CL exposure, whilst masculine notions perpetuated risky self-treatment practices among persons affected by CL [38,48,53]. In Brazil, de Oliveira, Bezerra Mm Fau - de Almeida [61] found that multi-bacillary leprosy was more prevalent among men. Conversely, others (n = 7) reported higher OSD, LF, tungiasis and CL prevalence among females [39,40,44,46,49,50,55]. Gendered activities, such as washing melon seeds in rivers [39], crop farming [49], staying at home [40,44] were reportedly key in shaping prevalence. Furthermore, women’s domestic responsibilities and male escort/female doctor requirements reportedly limited healthcare-seeking behaviour in some instances [46,55], and facilitated higher skin NTD rates among women. Another article attributed LF among children to women’s inability to recognise symptoms [54].

In studies measuring mental health outcomes (n = 6), across South, Southeast Asia, North Africa and Sub-Saharan Africa, women reportedly experienced severe stigma and psychological distress linked to scarring, social exclusion and failure to complete domestic chores [39,40,44,48,54,55]. Societal rejection, self-isolation and marriageability fears also disproportionately impacted women and girls [54] affected by LF in Thailand, with CL/ZCL said to be altering “women’s beauty” in Pakistan, Tunisia and Morocco [40,44,48]. In Tunisia, women with ZCL highlighted interpersonal conflict, whether family, social or professional, and reported a significant correlation between number of scars and stigma [40]. Whereas in a Nigerian study, self-reported stigma scores were highest among single men with OSD, compared to single women and ever-married women and men though the finding was not statistically significant [39].

*Economic factors*

In total, 16 studies explored economic factors. Poverty was repeatedly linked to disease prevalence by study authors (n = 8), with reliance on daily wages and high treatment costs reportedly impacting healthcare-seeking behaviour [46,48–50,53,55–57]. This included harmful CL self-treatment practices in Suriname to maintain hunting/gold/timber employment [53]. Low socio-economic status was found to increase CL risk in Pakistan [48]. Similarly, tungiasis reportedly clustered in poorer settings, where food was often prioritised over hygiene measures [49,50,57].

Others (n = 8) explored employment and educational detriments, primarily disability related. Podoconiosis was found to cause mobility loss, psychosocial strain and reduced productivity, whilst tungiasis drove school dropouts and work incapacity, limiting daily activities and sleep [41,49,50,57]. Social participation and livelihoods (e.g., fishing/farming) were further hindered by LF-related physical disability, with some traders reportedly unable to sell goods due to stigma in some studies [36,54]. Studies also reported that leprosy-associated neurological impairments disrupted social functioning [59], and CL impeded daily activities [55].

Eleven studies explored how certain occupations increased skin NTD risk. Agricultural activities (e.g., swamp wading) [39,43], livestock rearing [38,48,49] and occupations in jungles (e.g., gold/lumbar) [52,53] were found to increase exposure to vectors. This influenced OSD mobility and stigma dynamics in one study [39]. Additionally, certain livelihoods were identified as driving harmful self-treatment practices (e.g., battery acid in Amazonian rainforests) and regarding CL, there was increased risk in the children of labourers [48,53].

Few studies seemed to explore the role of wider socioeconomic contexts. As noted in section 3.4.1, economic collapse in Cameroon in the 1980s-1990s increased food crop farming and deforestation, which heightened exposure to BU-related bacteria [43]. Soldiers in Colombia were often recruited from poor backgrounds and could only be reimbursed for CL treatment once, which reportedly delayed reassignment requests and worsened health outcomes [52]. Across studies, high out-of-pocket costs were most reportedly prominent where healthcare facilities lacked drugs [48,53] and/or were hard to reach [53–55] and income levels were low [48–50,52,55].

*Conflict and migration*

Five studies explored the impact of conflict and migration; McNeilly, Mutebi [49] linked tungiasis transmission to migratory patterns. Other studies explicitly highlighted the association between war and CL, due to the destruction of infrastructure and displacement, among other factors [37,52,55,56].

#### 3.4.4 Cross-sectoral collaboration.

From reviewing the papers, we inferred that cross-sectoral collaboration was generally limited. 12 involved government or public health authorities, including national control programmes and ministries of health [49]. Nine partnered with international organisations such as WHO, Médecins Sans Frontières, and the Red Cross. The local health workforce participated in five studies, and community actors—schools, religious and local leaders—in another six. Only two collaborated with sectors beyond health (military, geology, meteorological services) [48,52]. Eight reported no cross-sectoral collaboration, working solely with research institutions [36,39,41,42,45,47,56,59].

### 3.5 Recommendations for good practice

Articles commonly called for multi-sector collaboration and integration (n = 12) and further evidence-based multidisciplinary research (n = 11), as well as community engagement and involvement (n = 11). Others emphasised a need for strengthened psychosocial support (n = 6), stronger and more intensive vector and reservoir control efforts (n = 6) and changes to governance and policy (n = 6). Some authors further made recommendations regarding treatment (n = 5), health system strengthening for CL (n = 4) and improving surveillance (n = 4).

## 4 Discussion

Whilst we identified some literature outlining the relationship between skin NTDs and mental health that considered factors aligned with the OH perspective, the nature and strength of associations and underlying biological, social, and ecological mechanisms remained poorly characterised, with the explicit interface between mental health and OH rarely discussed and very few intervention studies. In addition, most studies focused on a single disease and a limited subset of relevant factors, reflecting a lack of integrated thinking about these complex intersections. Even within the scope of OH itself, few articles explicitly addressed all three domains—environment, animals, and humans—and studies rarely employed intersectoral collaboration or study designs capable of meaningfully integrating expertise across human health, animal health, and the environmental discipline.

Regarding the social environment, poverty and economic marginalisation emerged as central themes, illustrating how interactions between livestock management, environmental conditions, and skin NTDs were embedded within broader social and socioeconomic contexts [49,50,62]. There is often an economic cost of contracting skin NTDs, which impacts health-seeking behaviour and/or poor socioeconomic conditions may increase transmission risks [63, 64]. In some cases, behaviours associated with increased skin NTD risk persisted despite awareness of these associations and the known social ramifications of skin NTDs, reflecting the constrained choices and structural limitations faced by people living in poverty [65,66]. In other cases, animals did not in themselves confer higher disease risk; rather, environmental degradation that limited people’s ability to keep livestock, fish, or hunt, which through the heightened risk of poor nutrition, poverty, migration and/or the prevalence of pathogens increased vulnerability to skin NTDs [42, 64, 65, 67]. In both cases, exposure to disease was driven by a lack of agency and choice which largely defined individual behaviour.

By contrast, exploration of the relationship between household economic conditions and skin NTDs as well as mental health conditions, through a OH lens, was largely absent from the studies we identified, despite the likely close, dynamic, and bidirectional associations between these domains [68,69]. Further research is needed to understand the extent to which a) skin NTDs contribute to economic loss (e.g., due to morbidity, stigma and/or health seeking costs); b) economic loss and/or the loss of livestock due to NTD control measures contribute to poor mental health outcomes; and c) mental health influences economic participation and productivity. Such research would contribute to a greater understanding of when, where and how to intervene most effectively, as well as how to assess both the economic and social benefits of these interventions. For example, interventions that address skin NTD related stigma may simultaneously improve mental health and strengthen social capital, support continued educational engagement and enhance economic productivity [70,71]. While enabling earlier care-seeking—including through community-based delivery models can reduce long-term disability [70,71]. These pathways are particularly relevant for the design of effective, multi-dimensional NTD control strategies. Yet, if mental health and livelihood considerations are not explicitly integrated into interventions, they may inadvertently exacerbate economic stressors or psychological distress. An obvious example is recognising the economic imperative and cultural traditions of keeping animals, which cannot be ignored in maintaining mental wellbeing.

Many of the environmental changes mentioned were related to climate change; however, as has been found elsewhere, explicit attribution of these changes to climate change—and thus clear identification of pathways for intervention—was limited [72]. Whereas, integrating the rapidly expanding evidence on climate change and health into OH analyses of mental health and skin NTDs can generate important insights and programmatic efficiencies, including improved translation of knowledge into effective action [72,73].

Stigma is well recognised as the major driver of depression and anxiety among people affected by skin NTDs [2,74]. The presumption that greater knowledge about biological causation inevitably leads to less stigma has been largely discredited [75]. Specifically, research evidence suggests that increasing awareness and providing biomedical explanations, when delivered without attention to social context and lived experience, may entrench stigma and social contact challenging attitudes is more impactful [76]. Nuanced recommendations are therefore needed to inform appropriate health behaviours, alongside the use of stigma-reduction interventions that have demonstrated greater effectiveness, such as community-level social contact interventions [2,77]. Such interventions have proven very effective in the fields of leprosy and LF [78,79], with involving persons with lived experience being effective in this context [68,80–84] Increasing knowledge alone can be helpful for changing help-seeking behaviour; however, when poorly framed or implemented, it can also generate resistance and suspicion. Any messaging should therefore be carefully designed and delivered in ways that are likely to reduce judgement and engender trust.

Another key finding was that few studies employed qualitative methodologies or explicitly prioritised the participation of persons affected by skin NTDs and their communities, including pluralistic providers who play a critical role in skin NTD care [85]. This gap is striking given both the limited existing evidence on the relationship between mental health, skin NTDs, and OH, and the recognition that addressing the syndemic burden of mental illness among persons affected requires context-specific, culturally grounded interventions [86]. As research seeks to further elucidate the relationship between OH, mental health, and skin NTDs, and to support the development of person-centred interventions that embed OH principles, the social sciences have a critical role to play. Further exploratory mixed-methods research is vital, particularly research that combines validated mental health assessment tools with participatory and qualitative approaches that centre the lived experiences, priorities, and explanatory models of persons affected and their communities. Such approaches are essential not only for improving measurement and intervention design, but also for ensuring that OH- and mental health–informed skin NTD responses are ethically grounded, acceptable, and responsive to local realities. Greater engagement with pluralistic providers (e.g., traditional and faith healers) may also offer important insights into help-seeking pathways, local concepts of distress, and opportunities for integrated mental health support within skin NTD care [14,85,87].

Overall, we hypothesise that OH plays a key role in shaping experiences of mental health in persons affected by skin NTDs, largely through SDH, such as changing environment leading to increased risk to traditional livelihoods, but the underlying mechanisms remain poorly characterised. Research that adopts a OH lens and intersectional approach, with an explicit focus on mental health would strengthen this understanding. In most cases, the direct links between OH determinants and mental wellbeing were absent from the papers reviewed. There is a need for further research and for operational and normative programmatic guidelines [11] to facilitate the development of more OH-sensitive interventions within NTD programmes. This is particularly the case for the field of skin NTDs and mental health, where the application of OH approaches are less implicit than, for example, vector control programmes. Using an OH lens to integrate skin NTDs and mental health in health system interventions may be particularly pertinent in reaching the most marginalised communities affected by climate change and humanitarian crises. Furthermore, it is critical that such endeavours build upon identifying synergies with current frameworks, rather than re-inventing the wheel. For example, OH approaches align well to person-centred care advocated by the WHO, including in the recently launched Essential Care Package to address Mental Health and Stigma for persons with NTDs [65]. These approaches are complementary and each share common principles, while adding nuance and being more appropriate for use in specific areas of investigation [88,89].

### 4.1 Strengths and limitations

This review makes a novel contribution by summarising a limited evidence-base and drawing on experience of experts to propose frameworks that can inform strategies that can pave the way for future research and practice in skin NTDs under the OH-approach.

As this paper formed part of a series of reviews, we sought to align methodological approaches across studies, including the selective use of AI tools to improve efficiency and consistency. Specifically, we used AI to support title and abstract screening (ASReview) and data extraction (NotebookLM). We implemented multiple safeguards to ensure accuracy and to mitigate recognised risks associated with AI use, such as overlooking key information or hallucination. We familiarised ourselves with each article prior to extraction using NotebookLM and ensured that the tool returned relevant verbatim quotations and generated summaries that referenced study results, enabling systematic cross-checking throughout the process. Additionally, once results were drafted, all included papers were re-read and cross-referenced (by IH) to ensure that no errors or omissions remained. ASReview was of limited utility; while it provided a structured platform for screening and labelling abstracts, the prioritisation algorithm performed poorly and did not meaningfully assist in ordering abstracts for review. Conversely, NotebookLM substantially increased the speed and efficiency of data extraction and, used in combination with manually checking information and iteratively amending the prompts, only resulted in a small number of omissions identified during quality checks. This suggests that when a cautious human-in-the-loop approach is applied, NotebookLM provides a promising tool for extracting literature. Despite these strengths, important concerns remain regarding the transparency, reproducibility, and potential bias of AI-assisted evidence synthesis, including risks of extraction bias, consistency of interpretations and AI sycophancy, underscoring the importance the mitigating strategies used. We chose to exclude grey literature to maintain methodological consistency with other reviews in the series and to prioritise peer-reviewed evidence. This decision may have resulted in the omission of programmatic, policy-focused, or implementation-level insights, particularly from low-resource settings. Another key limitation is that whilst our OH component was quite broad and did not require the term ‘One Health’ to be used, we may have been overly restrictive in our requirement for papers to address all four domains. This resulted in a small sample size, impeding the generalizability of our results. The criteria also led to us excluding relevant work on mental health, social and economic outcomes in skin NTDs that did not explicitly embed OH, introducing selection bias. Consequently, it is important to highlight that the findings reflect a subset of evidence which pertains to OH, rather than the fuller literature on NTDs and mental health. Finally, many studies did not use validated tools to directly measure mental health outcomes, restricting our ability to evaluate direct associations with OH determinants.

## 5 Conclusions

We found a range of studies highlighting important links between mental health, skin NTDs, and the OH dimensions; however, few studies explored the full range of relevant factors in an integrated manner. The literature consistently recognised mental health as important, but limitations in quantification and understanding mechanisms were acknowledged. Multi-sectoral collaboration and integration was seen as a key mechanism for ensuring mental health is addressed, and multidisciplinary research can provide guidance on how to do this. Such integration is fundamental to the OH approach, and it adds value to other comprehensive and integrated approaches like person-centred care, so should be utilised within service delivery and research. Applying methodological approaches used in related fields, such as intersectionality and syndemics, could add substantial value when addressing these overlapping domains. Ultimately, whilst this review was able to contribute to the evidence base for understanding NTDs framed around OH, more empirical studies directly measuring mental health outcomes in relation to OH determinants are required to support and validate the broader conceptual interpretations proposed. Nevertheless, the growing recognition of mental health within both the OH and skin NTD fields provides a strong foundation on which to build more integrated understanding and action.

## Supporting information

S1 Checklist PRISMA-ScR checklist. (DOCX)

S1 File Pubmed search strategy. (DOCX)

S2 File Data extraction questions. (DOCX)

S3 File Extraction guide. (DOCX)

S4 File Screening and extraction table. (XLSX)
