# Supplementary material for: Mental health, social determinants and One Health in skin neglected tropical diseases (NTDs): A scoping review
Source: PLoS Negl Trop Dis. 2026 Aug 6;20(8):e0014034. doi: 10.1371/journal.pntd.0014034 (PMC13485127; doi:10.1371/journal.pntd.0014034)
Supplement: Supplementary file 2 [file pntd.0014034.s002.docx]

**CONCEPT 1: SKIN NTDS**

"Buruli Ulcer"[Mesh] OR "Mycobacterium ulcerans"[Mesh] OR

"Leishmaniasis, Cutaneous"[Mesh] OR "Leishmaniasis, Mucocutaneous"[Mesh] OR "Leishmaniasis, Diffuse Cutaneous"[Mesh] OR

"Leprosy"[Mesh] OR "Mycobacterium leprae"[Mesh] OR

"Filariasis, Lymphatic"[Mesh] OR "Wuchereria bancrofti"[Mesh] OR "Brugia malayi"[Mesh] OR

"Chromoblastomycosis"[Mesh] OR

"Noma"[Mesh] OR

"Mycetoma"[Mesh] OR "Actinomycetoma"[Mesh] OR "Eumycetoma"[Mesh] OR

"Sporotrichosis"[Mesh] OR

"Onchocerciasis"[Mesh] OR "Onchocerca volvulus"[Mesh] OR

"Scabies"[Mesh] OR

"Tungiasis"[Mesh] OR

"Yaws"[Mesh] OR "Treponema pallidum"[Mesh]

OR

"Buruli ulcer"[tiab] OR "Mycobacterium ulcerans"[tiab] OR "buruli ulcers"[tiab]

OR "cutaneous leishmaniasis"[tiab] OR "American Tegumentary Leishmaniasis"[tiab] OR "post-kala-azar dermal leishmaniasis"[tiab] OR "PKDL"[tiab] OR "dermal leishmaniasis"[tiab] OR "localized cutaneous leishmaniasis"[tiab] OR "mucocutaneous leishmaniasis"[tiab] OR "mucosal leishmaniasis"[tiab] OR "diffuse cutaneous leishmaniasis"[tiab] OR "leishmaniasis recidivans"[tiab]

OR "Leprosy"[tiab] OR "Mycobacterium leprae"[tiab] OR "multibacillary leprosy"[tiab] OR "lepromatous leprosy"[tiab] OR "paucibacillary leprosy"[tiab] OR "borderline leprosy"[tiab] OR "tuberculoid leprosy"[tiab] OR "mycobacterium lepromatosis"[tiab]

OR "lymphatic filariasis"[tiab] OR "elephantiasis"[tiab] OR "Wuchereria bancrofti"[tiab] OR "Brugia malayi"[tiab] OR "Brugia timori"[tiab] OR "Brugia pahangi"[tiab] OR "bancrofti microfilaria"[tiab] OR "bancroftian filariasis"[tiab] OR "brugian filariasis"[tiab]

OR "chromoblastomycosis"[tiab] OR "chromoblastomycosis infection"[tiab] OR "dematiaceous fungi"[tiab] OR "Fonsecaea"[tiab] OR "Cladophialophora"[tiab] OR "Rhinocladiella"[tiab] OR "Phialophora"[tiab] OR "Exophiala"[tiab] OR "exophiala species"[tiab] OR "exophiala dermatitidis"[tiab]

OR "Noma"[tiab]

OR "Mycetoma"[tiab] OR "Maduromycosis"[tiab] OR "Madura Foot"[tiab] OR "Actinomycetoma"[tiab] OR "Eumycetoma"[tiab] OR "Madurella"[tiab] OR "madurella mycetomatis"[tiab]

OR "sporotrichosis"[tiab] OR "Sporothrix schenckii"[tiab] OR "Sporothrix"[tiab] OR "Sporothrix brasiliensis"[tiab] OR "Sporothrix globosa"[tiab]

OR "River blindness"[tiab] OR "onchocerciasis"[tiab] OR "Onchocerca volvulus"[tiab] OR "Onchocerca cervicalis"[tiab] OR "Onchocerca reticulata"[tiab] OR "Onchocerca gibsoni"[tiab] OR "onchocerca ochengi"[tiab]

OR "Scabies"[tiab] OR "Sarcoptes scabiei"[tiab] OR "Sarcoptes scabiei var hominis"[tiab] OR "sarcoptes mite"[tiab] OR "sarcoptic itch"[tiab] OR "sarcoptic acariasis"[tiab] OR "mange"[tiab] OR "crusted scabies"[tiab] OR "scabiei mites"[tiab]

OR "Tungiasis"[tiab] OR "Tunga penetrans"[tiab]

OR "yaws"[tiab] OR "Treponema pallidum pertenue"[tiab] OR "Treponema pallidum subsp. pertenue"[tiab] OR "Treponema pallidum subspecies pertenue"[tiab] OR "endemic treponematosis"[tiab] OR "endemic treponematoses"[tiab] OR "treponematos*"[tiab] OR "framboesia"[tiab] OR "Patek"[tiab] OR "Parangi"[tiab] OR "Bouba"[tiab] OR "Coco yaws"[tiab] OR "Goundou"[tiab]

**AND**

**Concept 2: One Health**

"One Health"[MeSH Terms] OR "Zoonoses"[MeSH Terms] OR "Zoonotic Diseases"[MeSH Terms] OR "Animals, Domestic"[MeSH Terms] OR "Animals, Wild"[MeSH Terms] OR "Human-Animal Bond"[MeSH Terms] OR "Pets"[MeSH Terms] OR "Rodentia"[MeSH Terms] OR "Bats"[MeSH Terms] OR "Chiroptera"[MeSH Terms] OR "Livestock"[MeSH Terms] OR "Agriculture"[MeSH Terms] OR "Organic Agriculture"[MeSH Terms] OR "Gardening"[MeSH Terms] OR

("one"[All Fields] AND "health"[All Fields]) OR "one health"[All Fields] OR "one health"[tiab] OR "Human-Animal Interaction"[MeSH Terms] OR "animals, wild"[MeSH Terms] OR "animals, domestic"[MeSH Terms] OR "Organic Agriculture"[MeSH Terms] OR "Gardening"[MeSH Terms] OR "Agriculture"[MeSH Terms]

"One Health"[tiab] OR "Zoonotic Disease*"[tiab] OR "Bats"[tiab] OR "Chiroptera"[tiab] OR "Organic Agriculture"[tiab] OR ("human*"[tiab] AND "animal*"[tiab]) OR ("human*"[tiab] AND "environment*"[tiab]) OR ("anima*"[tiab] AND "environment*"[tiab]) OR "human-animal interaction*"[tiab] OR "biophilia"[tiab] OR "nature connection*"[tiab] OR "ecotherapy*"[tiab] OR

"human–environment interaction*"[tiab] OR "environmental exposure*"[tiab] OR

"pet ownership*"[tiab] OR "companion animal*"[tiab] OR "service animal*"[tiab] OR

"animal companionship*"[tiab] OR "wildlife interaction*"[tiab] OR "gardening"[tiab] OR

"horticultural"[tiab] OR "food source"[tiab] OR "food preparation"[tiab] OR

"reservoir"[tiab] OR "intermediate host"[tiab] OR "zoono*"[tiab] OR

"anthropozoonosis"[tiab] OR "domestic animal*"[tiab] OR "peridomestic"[tiab] OR

"sylvatic"[tiab] OR "livestock"[tiab] OR "subsistence farming"[tiab] OR

"bats"[tiab] OR "rodents"[tiab] OR "rock hyrax"[tiab] OR "chicken*"[tiab] OR

"cow"[tiab] OR "cows"[tiab] OR "goat*"[tiab] OR "crop*"[tiab] OR

"une seule santé"[tiab] OR "une santé seule"[tiab] OR "santé unique"[tiab] OR "santé humaine et animale"[tiab] OR "interaction homme-animal"[tiab] OR "réservoir"[tiab] OR "hôte intermédiaire"[tiab] OR "zoonose"[tiab] OR "zoonose anthropozoonotique"[tiab] OR "animal domestique"[tiab] OR "péridomestique"[tiab] OR "sylvatique"[tiab] OR "élevage"[tiab] OR

"agriculture de subsistance"[tiab] OR "chauves-souris"[tiab] OR "rongeurs"[tiab] OR "daman des rochers"[tiab]

OR "una sola salud"[tiab] OR "salud única"[tiab] OR "interacción humano-animal"[tiab] OR "reservorio"[tiab] OR "huésped intermediario"[tiab] OR "zoonosis"[tiab] OR "antropozoonosis"[tiab] OR "animal doméstico"[tiab] OR "peridoméstico"[tiab] OR "silvático"[tiab] OR "ganado"[tiab] OR "agricultura de subsistencia"[tiab] OR "murciélagos"[tiab] OR "roedores"[tiab] OR "daman de las rocas"[tiab]

OR "eine gesundheit"[tiab] OR "mensch-tier-interaktion"[tiab] OR "tier-mensch-interaktion"[tiab] OR "wirtstier"[tiab] OR "zwischenwirt"[tiab] OR "zoonose"[tiab] OR "anthropozoonose"[tiab] OR "haustier"[tiab] OR "peridomestisch"[tiab] OR "sylvatisch"[tiab] OR "vieh"[tiab] OR "subsistenzlandwirtschaft"[tiab] OR "fledermäuse"[tiab] OR "nagetiere"[tiab] OR "klippschliefer"[tiab]

OR "uma só saúde"[tiab] OR "saúde única"[tiab] OR "interação humano-animal"[tiab] OR "reservatório"[tiab] OR "hospedeiro intermediário"[tiab] OR "zoonose"[tiab] OR "antropozoonose"[tiab] OR "animal doméstico"[tiab] OR "peridoméstico"[tiab] OR "silvático"[tiab] OR "gado"[tiab] OR "agricultura de subsistência"[tiab] OR "morcegos"[tiab] OR "roedores"[tiab] OR "procávia-das-rochas"[tiab]

**AND**

**CONCEPT 3: MENTAL HEALTH**

"mental health"[MeSH Terms] OR "Psychological Well-Being"[MeSH Terms] OR "Emotions"[MeSH Terms] OR "Quality of Life"[MeSH Terms]

OR "mental health"[tiab] OR "psychological well-being"[tiab] OR “wellbeing”[tiab] OR “well-being”[tiab] OR “emotion*”[tiab] OR “affect*”[tiab]

OR "emotional regulation*"[tiab] OR "mental wellness"[tiab] OR psych*[tiab] OR "quality of life"[tiab]

OR "santé mentale"[tiab] OR "bien-être psychologique"[tiab] OR "bien-être"[tiab] OR "émotions"[tiab] OR "régulation émotionnelle"[tiab]

OR "bien-être mental"[tiab] OR "qualité de vie"[tiab]

OR "salud mental"[tiab] OR "bienestar psicológico"[tiab] OR "bienestar"[tiab] OR "emociones"[tiab] OR "regulación emocional"[tiab]

OR "bienestar mental"[tiab] OR "calidad de vida"[tiab]

OR "psychische Gesundheit"[tiab] OR "psychisches Wohlbefinden"[tiab] OR "Wohlbefinden"[tiab] OR "Emotionen"[tiab]

OR "emotionale Regulierung"[tiab] OR "psychisches Wohlsein"[tiab] OR "Lebensqualität"[tiab]

OR "saúde mental"[tiab] OR "bem-estar psicológico"[tiab] OR "bem-estar"[tiab] OR "emoções"[tiab]

OR "regulação emocional"[tiab] OR "bem-estar mental"[tiab] OR "qualidade de vida"[tiab] **OR**

"Mental Disorders"[MeSH] OR "Psychopathology"[MeSH]

OR “psychopatholog*”[tiab] OR "mental illness*"[tiab] OR "psychiatric disorder*"[tiab] OR "psychological disorder*"[tiab]

OR "emotional disorder*"[tiab] OR "behavioral disorder*"[tiab] OR "mental disorder*"[tiab] OR "internalizing disorder*"[tiab]

OR "externalizing disorder*"[tiab] OR "psychosis"[tiab] OR "neurosis"[tiab] OR "depression"[tiab] OR "anxiety"[tiab]

OR "bipolar disorder"[tiab] OR "schizophrenia"[tiab] OR "PTSD"[tiab] OR "OCD"[tiab]

OR "psychopathologie"[tiab] OR "trouble mental"[tiab] OR "maladie mentale"[tiab] OR "trouble psychiatrique"[tiab]

OR "trouble émotionnel"[tiab] OR "psychose"[tiab] OR "névrose"[tiab] OR "dépression"[tiab] OR "anxiété"[tiab]

OR "schizophrénie"[tiab]

OR "psicopatología"[tiab] OR "enfermedad mental"[tiab] OR "trastorno psiquiátrico"[tiab] OR "trastorno psicológico"[tiab]

OR "psicosis"[tiab] OR "neurosis"[tiab] OR "depresión"[tiab] OR "ansiedad"[tiab] OR "esquizofrenia"[tiab]

OR "Psychopathologie"[tiab] OR "psychische Störung"[tiab] OR "Geisteskrankheit"[tiab] OR "psychotische Störung"[tiab]

OR "Depression"[tiab] OR "Angst"[tiab] OR "Schizophrenie"[tiab]

OR "psicopatologia"[tiab] OR "transtorno mental"[tiab] OR "doença mental"[tiab] OR "transtorno psiquiátrico"[tiab]

OR "psicose"[tiab] OR "neurose"[tiab] OR "depressão"[tiab] OR "ansiedade"[tiab] OR "esquizofrenia"[tiab] **OR** "Social Stigma"[Mesh] OR "Social Discrimination"[Mesh] OR "Shame"[Mesh] OR "Prejudice"[Mesh]

OR "stigma"[tiab] OR "stigmatization"[tiab] OR "discrimination"[tiab] OR "prejudice"[tiab] OR "shame"[tiab]

OR "self stigma"[tiab] OR "public stigma"[tiab] OR "perceived stigma"[tiab] OR "enacted stigma"[tiab]

OR "stigmatisation"[tiab] OR "discrimination sociale"[tiab] OR "préjugé"[tiab] OR "honte"[tiab]

OR "estigmatización"[tiab] OR "discriminación social"[tiab] OR "prejuicio"[tiab] OR "vergüenza"[tiab]

OR "Stigmatisierung"[tiab] OR "soziale Diskriminierung"[tiab] OR "Vorurteil"[tiab] OR "Scham"[tiab]

OR "estigmatização"[tiab] OR "discriminação social"[tiab] OR "preconceito"[tiab] OR "vergonha"[tiab] **OR** "Disabled Persons"[Mesh] OR "Disability Evaluation"[Mesh] OR "Developmental Disabilities"[Mesh] OR "Intellectual Disability"[Mesh]

OR "Disability Discrimination"[Mesh] OR "Disability Studies"[Mesh] OR "Persons with Disabilities"[Mesh] OR "Persons with Intellectual Disabilities"[Mesh]

OR "disabilit*"[tiab] OR "impairment*"[tiab] OR "functional limitation*"[tiab] OR "developmental delay*"[tiab]

OR "cognitive impairment*"[tiab] OR "mobility limitation*"[tiab] OR "learning disabilit*"[tiab]

OR "handicap"[tiab] OR "incapacité"[tiab] OR "déficience*"[tiab] OR "limitation fonctionnelle"[tiab]

OR "retard de développement"[tiab] OR "trouble cognitif"[tiab] OR "limitation motrice"[tiab] OR "trouble de l’apprentissage"[tiab]

OR "discapacidad"[tiab] OR "impedimento"[tiab] OR "limitación funcional"[tiab] OR "retraso del desarrollo"[tiab]

OR "deterioro cognitivo"[tiab] OR "limitación motora"[tiab] OR "dificultad de aprendizaje"[tiab]

OR "Behinderung"[tiab] OR "Beeinträchtigung"[tiab] OR "funktionale Einschränkung"[tiab]

OR "Entwicklungsverzögerung"[tiab] OR "kognitive Beeinträchtigung"[tiab] OR "Mobilitätseinschränkung"[tiab]

OR "Lernbehinderung"[tiab]

OR "deficiência"[tiab] OR "incapacidade"[tiab] OR "limitação funcional"[tiab] OR "atraso no desenvolvimento"[tiab]

OR "comprometimento cognitivo"[tiab] OR "limitação de mobilidade"[tiab] OR "dificuldade de aprendizagem"[tiab]

**AND**

**CONCEPT 4 – SOCIAL DETERMINANTS OF HEALTH**

"Sex Factors"[MeSH] OR "Gender Identity"[MeSH] OR "Sex Characteristics"[MeSH] OR "Age Factors"[MeSH] OR "Ethnicity"[MeSH] OR "Population Density"[MeSH] OR "Cultural Diversity"[MeSH] OR "Longevity"[MeSH] OR "Life Expectancy"[MeSH] OR "Survival Rate"[MeSH] OR "Survival Analysis"[MeSH] OR "gender*"[tiab] OR "sex"[tiab] OR "sex factor*"[tiab] OR "gender identit*"[tiab] OR "sex characteristic*"[tiab] OR "sex difference*"[tiab] OR "gender-based"[tiab] OR "Age Factors"[tiab] OR "age"[tiab] OR "adolescent"[tiab] OR "adult"[tiab] OR "aged"[tiab] OR "geriatric"[tiab] OR "Ethnicity"[tiab] OR "Ethnic Group*"[tiab] OR "ethnicity"[tiab] OR "race"[tiab] OR "Population Density"[tiab] OR "Multiculturalism"[tiab] OR "diversity"[tiab] OR "community diversity"[tiab] OR "Longevity"[tiab] OR "longevity"[tiab] OR "Life Expectancy"[tiab] OR "life expectancy"[tiab] OR "Survival Rate"[tiab] OR "survival"[tiab] OR "Survival Analysis"[tiab] OR "abstinence"[tiab] OR "marriageability"[tiab] OR "sexual interest"[tiab] OR "sexuality"[tiab] OR "autochthonous population"[tiab] OR "indigenous population"[tiab] OR "sexe"[tiab] OR "genre"[tiab] OR "identité de genre"[tiab] OR "caractéristiques sexuelles"[tiab] OR "différences sexuelles"[tiab] OR "basé sur le genre"[tiab] OR "facteurs liés à l'âge"[tiab] OR "âge"[tiab] OR "adolescent"[tiab] OR "adulte"[tiab] OR "âgé"[tiab] OR "gériatrique"[tiab] OR "ethnicité"[tiab] OR "groupe ethnique"[tiab] OR "densité de population"[tiab] OR "multiculturalisme"[tiab] OR "diversité"[tiab] OR "diversité communautaire"[tiab] OR "longévité"[tiab] OR "espérance de vie"[tiab] OR "taux de survie"[tiab] OR "analyse de survie"[tiab] OR "abstinence"[tiab] OR "aptitude au mariage"[tiab] OR "intérêt sexuel"[tiab] OR "sexualité"[tiab] OR "population autochtone"[tiab] OR "population indigène"[tiab] OR "sexo"[tiab] OR "género"[tiab] OR "identidad de género"[tiab] OR "características sexuales"[tiab] OR "diferencias sexuales"[tiab] OR "basado en género"[tiab] OR "factores de edad"[tiab] OR "edad"[tiab] OR "adolescente"[tiab] OR "adulto"[tiab] OR "anciano"[tiab] OR "geriátrico"[tiab] OR "etnicidad"[tiab] OR "grupo étnico"[tiab] OR "densidad de población"[tiab] OR "multiculturalismo"[tiab] OR "diversidad"[tiab] OR "diversidad comunitaria"[tiab] OR "longevidad"[tiab] OR "esperanza de vida"[tiab] OR "tasa de supervivencia"[tiab] OR "análisis de supervivencia"[tiab] OR "abstinencia"[tiab] OR "aptitud matrimonial"[tiab] OR "interés sexual"[tiab] OR "sexualidad"[tiab] OR "población autóctona"[tiab] OR "población indígena"[tiab] OR "Geschlecht"[tiab] OR "Gender"[tiab] OR "Geschlechtsidentität"[tiab] OR "geschlechtliche Merkmale"[tiab] OR "Geschlechtsunterschiede"[tiab] OR "geschlechtsbasiert"[tiab] OR "Altersfaktoren"[tiab] OR "Alter"[tiab] OR "Jugendlicher"[tiab] OR "Erwachsener"[tiab] OR "älter"[tiab] OR "geriatrisch"[tiab] OR "Ethnizität"[tiab] OR "ethnische Gruppe"[tiab] OR "Bevölkerungsdichte"[tiab] OR "Multikulturalismus"[tiab] OR "Vielfalt"[tiab] OR "gemeinschaftliche Vielfalt"[tiab] OR "Langlebigkeit"[tiab] OR "Lebenserwartung"[tiab] OR "Überlebensrate"[tiab] OR "Überlebensanalyse"[tiab] OR "Abstinenz"[tiab] OR "Heiratsfähigkeit"[tiab] OR "sexuelles Interesse"[tiab] OR "Sexualität"[tiab] OR "autochthone Bevölkerung"[tiab] OR "indigene Bevölkerung"[tiab] OR "sexo"[tiab] OR "gênero"[tiab] OR "identidade de gênero"[tiab] OR "características sexuais"[tiab] OR "diferenças sexuais"[tiab] OR "baseado no gênero"[tiab] OR "fatores etários"[tiab] OR "idade"[tiab] OR "adolescente"[tiab] OR "adulto"[tiab] OR "idoso"[tiab] OR "geriátrico"[tiab] OR "etnicidade"[tiab] OR "grupo étnico"[tiab] OR "densidade populacional"[tiab] OR "multiculturalismo"[tiab] OR "diversidade"[tiab] OR "diversidade comunitária"[tiab] OR "longevidade"[tiab] OR "expectativa de vida"[tiab] OR "taxa de sobrevivência"[tiab] OR "análise de sobrevivência"[tiab] OR "abstinência"[tiab] OR "aptidão para o casamento"[tiab] OR "interesse sexual"[tiab] OR "sexualidade"[tiab] OR "população autóctone"[tiab] OR "população indígena"[tiab] **OR**  "economics"[MeSH Subheading] OR "income"[MeSH Terms] OR "financial stress"[MeSH Terms] OR "Medically Uninsured"[Mesh] OR "Malnutrition"[Mesh] OR "Food Security"[Mesh] OR "Poverty"[MeSH] OR "Socioeconomic Factors"[MeSH] OR "economic*"[tiab] OR "cost*"[tiab] OR "financial*"[tiab] OR "affordability*"[tiab] OR "income*"[tiab] OR "socioeconomic*"[tiab] OR "economic barrier*"[tiab] OR "cost-benefit"[tiab] OR "economic burden*"[tiab] OR "financial burden"[tiab] OR "economic impact"[tiab] OR "cost* of illness"[tiab] OR "economic strain"[tiab] OR "financial hardship"[tiab] OR "povert*"[tiab] OR "low-income"[tiab] OR "low income"[tiab] OR "economic hardship*"[tiab] OR "financial hardship*"[tiab] OR "economic disadvantag*"[tiab] OR "socioeconomic status"[tiab] OR "socioeconomic deprivation"[tiab] OR "income inequalit*"[tiab] OR "social class"[tiab] OR "education"[tiab] OR "educational attainment"[tiab] OR "literacy"[tiab] OR "food security"[tiab] OR "food insecurity"[tiab] OR "nutritional deprivation"[tiab] OR "deprivation"[tiab] OR "housing instability"[tiab] OR "basic needs"[tiab] OR "inequities"[tiab] OR "debt*"[tiab] OR "asset*"[tiab] OR "cash*"[tiab] OR "financial strain"[tiab] OR "economic recession*"[tiab] OR "macroeconomic"[tiab] OR "livelihood*"[tiab] OR "unemploy*"[tiab] OR "employ*"[tiab] OR "financial stress*"[tiab] OR "uninsured"[tiab] OR "insur*"[tiab] OR "malnutrition"[tiab] OR "food insecurity"[tiab] OR "food security"[tiab] OR "Transportation cost*"[tiab] OR "economic disparity*"[tiab] OR "économie"[tiab] OR "revenu"[tiab] OR "stress financier"[tiab] OR "coût"[tiab] OR "financier"[tiab] OR "accessibilité financière"[tiab] OR "revenus faibles"[tiab] OR "socioéconomique"[tiab] OR "barrière économique"[tiab] OR "fardeau économique"[tiab] OR "fardeau financier"[tiab] OR "impact économique"[tiab] OR "coût de la maladie"[tiab] OR "pression économique"[tiab] OR "privation économique"[tiab] OR "inégalités de revenus"[tiab] OR "classe sociale"[tiab] OR "éducation"[tiab] OR "niveau d'instruction"[tiab] OR "alphabétisation"[tiab] OR "sécurité alimentaire"[tiab] OR "insécurité alimentaire"[tiab] OR "privation nutritionnelle"[tiab] OR "privation"[tiab] OR "instabilité du logement"[tiab] OR "besoins de base"[tiab] OR "inégalités"[tiab] OR "economía"[tiab] OR "ingresos"[tiab] OR "estrés financiero"[tiab] OR "costo"[tiab] OR "financiero"[tiab] OR "asequibilidad"[tiab] OR "bajos ingresos"[tiab] OR "socioeconómico"[tiab] OR "barrera económica"[tiab] OR "carga económica"[tiab] OR "carga financiera"[tiab] OR "impacto económico"[tiab] OR "costo de la enfermedad"[tiab] OR "tensión económica"[tiab] OR "privación económica"[tiab] OR "desigualdad de ingresos"[tiab] OR "clase social"[tiab] OR "educación"[tiab] OR "nivel educativo"[tiab] OR "alfabetización"[tiab] OR "seguridad alimentaria"[tiab] OR "inseguridad alimentaria"[tiab] OR "privación nutricional"[tiab] OR "privación"[tiab] OR "inestabilidad habitacional"[tiab] OR "necesidades básicas"[tiab] OR "desigualdades"[tiab] OR "Wirtschaft"[tiab] OR "Einkommen"[tiab] OR "finanzieller Stress"[tiab] OR "Kosten"[tiab] OR "finanziell"[tiab] OR "Bezahlbarkeit"[tiab] OR "niedriges Einkommen"[tiab] OR "sozioökonomisch"[tiab] OR "wirtschaftliche Barriere"[tiab] OR "wirtschaftliche Belastung"[tiab] OR "finanzielle Belastung"[tiab] OR "wirtschaftlicher Einfluss"[tiab] OR "Krankheitskosten"[tiab] OR "wirtschaftlicher Druck"[tiab] OR "wirtschaftliche Notlage"[tiab] OR "Einkommensungleichheit"[tiab] OR "soziale Klasse"[tiab] OR "Bildung"[tiab] OR "Bildungsstand"[tiab] OR "Alphabetisierung"[tiab] OR "Ernährungssicherheit"[tiab] OR "Ernährungsunsicherheit"[tiab] OR "ernährungsbedingte Notlage"[tiab] OR "Entbehrung"[tiab] OR "Wohnungsinstabilität"[tiab] OR "Grundbedürfnisse"[tiab] OR "Ungleichheiten"[tiab] OR "economia"[tiab] OR "renda"[tiab] OR "estresse financeiro"[tiab] OR "custo"[tiab] OR "financeiro"[tiab] OR "acessibilidade financeira"[tiab] OR "baixa renda"[tiab] OR "socioeconômico"[tiab] OR "barreira econômica"[tiab] OR "fardo econômico"[tiab] OR "fardo financeiro"[tiab] OR "impacto econômico"[tiab] OR "custo da doença"[tiab] OR "pressão econômica"[tiab] OR "privação econômica"[tiab] OR "desigualdade de renda"[tiab] OR "classe social"[tiab] OR "educação"[tiab] OR "escolaridade"[tiab] OR "alfabetização"[tiab] OR "segurança alimentar"[tiab] OR "insegurança alimentar"[tiab] OR "privação nutricional"[tiab] OR "privação"[tiab] OR "instabilidade habitacional"[tiab] OR "necessidades básicas"[tiab] OR "desigualdades"[tiab] **OR** "Residence Characteristics"[Mesh] OR "Neighborhood Characteristics"[Mesh] OR "Community Health Centers"[Mesh] OR "Housing Quality"[Mesh] OR "Housing"[Mesh] OR "Accident Prevention"[Mesh] OR "Social Deprivation"[Mesh] OR "neighborhood*"[tiab] OR "local area*"[tiab] OR "setting*"[tiab] OR "housing quality*"[tiab] OR "overcrowd*"[tiab] OR "disinvest*"[tiab] OR "security*"[tiab] OR "safety*"[tiab] OR "public facility*"[tiab] OR "recreation*"[tiab] OR "park*"[tiab] OR "urban"[tiab] OR "rural"[tiab] OR "peri urban"[tiab] OR "quartier"[tiab] OR "zone locale"[tiab] OR "environnement"[tiab] OR "qualité du logement"[tiab] OR "surpopulation"[tiab] OR "désinvestissement"[tiab] OR "sécurité"[tiab] OR "sûreté"[tiab] OR "infrastructure publique"[tiab] OR "loisirs"[tiab] OR "espace vert"[tiab] OR "parc"[tiab] OR "urbain"[tiab] OR "rural"[tiab] OR "périurbain"[tiab] OR "vecindario"[tiab] OR "zona local"[tiab] OR "entorno"[tiab] OR "calidad de la vivienda"[tiab] OR "hacinamiento"[tiab] OR "desinversión"[tiab] OR "seguridad"[tiab] OR "protección"[tiab] OR "instalaciones públicas"[tiab] OR "recreación"[tiab] OR "espacio verde"[tiab] OR "parque"[tiab] OR "urbano"[tiab] OR "rural"[tiab] OR "periurbano"[tiab] OR "Nachbarschaft"[tiab] OR "lokales Gebiet"[tiab] OR "Umgebung"[tiab] OR "Wohnqualität"[tiab] OR "Überbelegung"[tiab] OR "Desinvestition"[tiab] OR "Sicherheit"[tiab] OR "Schutz"[tiab] OR "öffentliche Einrichtung"[tiab] OR "Freizeit"[tiab] OR "Grünfläche"[tiab] OR "Park"[tiab] OR "städtisch"[tiab] OR "ländlich"[tiab] OR "periurban"[tiab] OR "vizinhança"[tiab] OR "área local"[tiab] OR "ambiente"[tiab] OR "qualidade da habitação"[tiab] OR "superlotação"[tiab] OR "desinvestimento"[tiab] OR "segurança"[tiab] OR "proteção"[tiab] OR "instalações públicas"[tiab] OR "recreação"[tiab] OR "espaço verde"[tiab] OR "parque"[tiab] OR "urbano"[tiab] OR "rural"[tiab] OR "periurbano"[tiab] **OR** "Natural Disasters"[Mesh] OR "Climate Change"[Mesh] OR "Environmental Pollution"[Mesh] OR "Armed Conflicts"[Mesh] OR "Human Migration"[Mesh] OR "Global Warming"[MeSH] OR "Extreme Weather"[MeSH] OR "Extreme Cold"[MeSH] OR "Extreme Heat"[MeSH] OR "Environmental Exposure"[MeSH] OR "Climate"[MeSH] OR "Weather"[MeSH] OR "Droughts"[MeSH] OR "Floods"[MeSH] OR "Temperature"[MeSH] OR "Greenhouse Effect"[MeSH] OR "Air Pollution"[MeSH] OR "Environmental Health"[MeSH] OR "Fires"[MeSH] OR "Wildfires"[MeSH] OR "Accidents, Occupational"[Mesh] OR "industrial disaster*"[tiab] OR "chemical spill*"[tiab] OR "oil spill*"[tiab] OR "natural disaster*"[tiab] OR "earthquake*"[tiab] OR "flood*"[tiab] OR "hurricane*"[tiab] OR "cyclone*"[tiab] OR "wildfire*"[tiab] OR "drought*"[tiab] OR "landslide*"[tiab] OR "climate change*"[tiab] OR "global warming*"[tiab] OR "environmental hazard*"[tiab] OR "environmental degradation*"[tiab] OR "war"[tiab] OR "conflict*"[tiab] OR "forced migration*"[tiab] OR "refugee*"[tiab] OR "internally displaced person*"[tiab] OR "informal settlement*"[tiab] OR "environmental cris*"[tiab] OR "displacement*"[tiab] OR "climate variabilit*"[tiab] OR "climate crisis"[tiab] OR "climate-related"[tiab] OR "changing climate"[tiab] OR "climate emergency"[tiab] OR "global heating"[tiab] OR "climate shock*"[tiab] OR "weather variabilit*"[tiab] OR "extreme weather"[tiab] OR "extreme heat"[tiab] OR "extreme cold"[tiab] OR "temperature rise"[tiab] OR "temperature increase"[tiab] OR "heatwave*"[tiab] OR "storm*"[tiab] OR "bushfire*"[tiab] OR "fire season"[tiab] OR "climate-induced displacement"[tiab] OR "climate migration"[tiab] OR "environmental migration"[tiab] OR "ecosystem degradation"[tiab] OR "ecological cris*"[tiab] OR "climate hazard*"[tiab] OR "environmental stress*"[tiab] OR "climate stress*"[tiab] OR "sea level rise"[tiab] OR "coastal erosion"[tiab] OR "desertification"[tiab] OR "glacial melt"[tiab] OR "ice loss"[tiab] OR "pollution"[tiab] OR "catastrophe naturelle"[tiab] OR "tremblement de terre"[tiab] OR "inondation"[tiab] OR "ouragan"[tiab] OR "cyclone"[tiab] OR "feu de forêt"[tiab] OR "sécheresse"[tiab] OR "glissement de terrain"[tiab] OR "changement climatique"[tiab] OR "réchauffement climatique"[tiab] OR "risque environnemental"[tiab] OR "dégradation environnementale"[tiab] OR "guerre"[tiab] OR "conflit"[tiab] OR "migration forcée"[tiab] OR "réfugié"[tiab] OR "personne déplacée intérieure"[tiab] OR "quartier informel"[tiab] OR "crise environnementale"[tiab] OR "déplacement"[tiab] OR "variabilité climatique"[tiab] OR "crise climatique"[tiab] OR "lié au climat"[tiab] OR "climat changeant"[tiab] OR "urgence climatique"[tiab] OR "choc climatique"[tiab] OR "variabilité météorologique"[tiab] OR "chaleur extrême"[tiab] OR "froid extrême"[tiab] OR "hausse de température"[tiab] OR "augmentation de température"[tiab] OR "vague de chaleur"[tiab] OR "tempête"[tiab] OR "feu de brousse"[tiab] OR "saison des incendies"[tiab] OR "déplacement climatique"[tiab] OR "migration climatique"[tiab] OR "migration environnementale"[tiab] OR "dégradation de l'écosystème"[tiab] OR "crise écologique"[tiab] OR "stress environnemental"[tiab] OR "stress climatique"[tiab] OR "élévation du niveau de la mer"[tiab] OR "érosion côtière"[tiab] OR "désertification"[tiab] OR "fonte des glaciers"[tiab] OR "perte de glace"[tiab] OR "desastre natural"[tiab] OR "terremoto"[tiab] OR "inundación"[tiab] OR "huracán"[tiab] OR "ciclón"[tiab] OR "incendio forestal"[tiab] OR "sequía"[tiab] OR "deslizamiento de tierra"[tiab] OR "cambio climático"[tiab] OR "calentamiento global"[tiab] OR "peligro ambiental"[tiab] OR "degradación ambiental"[tiab] OR "guerra"[tiab] OR "conflicto"[tiab] OR "migración forzada"[tiab] OR "refugiado"[tiab] OR "desplazado interno"[tiab] OR "asentamiento informal"[tiab] OR "crisis ambiental"[tiab] OR "desplazamiento"[tiab] OR "variabilidad climática"[tiab] OR "crisis climática"[tiab] OR "relacionado con el clima"[tiab] OR "clima cambiante"[tiab] OR "emergencia climática"[tiab] OR "choque climático"[tiab] OR "variabilidad del clima"[tiab] OR "clima extremo"[tiab] OR "calor extremo"[tiab] OR "frío extremo"[tiab] OR "aumento de temperatura"[tiab] OR "ola de calor"[tiab] OR "tormenta"[tiab] OR "incendio forestal"[tiab] OR "temporada de incendios"[tiab] OR "desplazamiento climático"[tiab] OR "migración climática"[tiab] OR "migración ambiental"[tiab] OR "degradación del ecosistema"[tiab] OR "crisis ecológica"[tiab] OR "estrés ambiental"[tiab] OR "estrés climático"[tiab] OR "aumento del nivel del mar"[tiab] OR "erosión costera"[tiab] OR "desertificación"[tiab] OR "derretimiento de glaciares"[tiab] OR "pérdida de hielo"[tiab] OR "contaminación"[tiab] OR "Naturkatastrophe"[tiab] OR "Erdbeben"[tiab] OR "Überschwemmung"[tiab] OR "Hurrikan"[tiab] OR "Zyklon"[tiab] OR "Waldbrand"[tiab] OR "Dürre"[tiab] OR "Erdrutsch"[tiab] OR "Klimawandel"[tiab] OR "globale Erwärmung"[tiab] OR "Umweltrisiko"[tiab] OR "Umweltverschlechterung"[tiab] OR "Krieg"[tiab] OR "Konflikt"[tiab] OR "erzwungene Migration"[tiab] OR "Flüchtling"[tiab] OR "Binnenvertriebene"[tiab] OR "informelle Siedlung"[tiab] OR "Umweltkrise"[tiab] OR "Vertreibung"[tiab] OR "klimatische Variabilität"[tiab] OR "Klimakrise"[tiab] OR "klimabedingt"[tiab] OR "sich veränderndes Klima"[tiab] OR "Klima-Notfall"[tiab] OR "Klimaschock"[tiab] OR "Wettervariabilität"[tiab] OR "extremes Wetter"[tiab] OR "extreme Hitze"[tiab] OR "extreme Kälte"[tiab] OR "Temperaturanstieg"[tiab] OR "Hitzewelle"[tiab] OR "Sturm"[tiab] OR "Buschfeuer"[tiab] OR "Feuersaison"[tiab] OR "klimabedingte Vertreibung"[tiab] OR "Klimamigration"[tiab] OR "Umweltmigration"[tiab] OR "Ökosystemdegradation"[tiab] OR "ökologische Krise"[tiab] OR "Umweltstress"[tiab] OR "Klimastress"[tiab] OR "Anstieg des Meeresspiegels"[tiab] OR "Küstenerosion"[tiab] OR "Desertifikation"[tiab] OR "Gletscherschmelze"[tiab] OR "Eisverlust"[tiab] OR "Verschmutzung"[tiab] OR "desastre natural"[tiab] OR "terremoto"[tiab] OR "inundação"[tiab] OR "furacão"[tiab] OR "ciclone"[tiab] OR "incêndio florestal"[tiab] OR "seca"[tiab] OR "deslizamento de terra"[tiab] OR "mudança climática"[tiab] OR "aquecimento global"[tiab] OR "risco ambiental"[tiab] OR "degradação ambiental"[tiab] OR "guerra"[tiab] OR "conflito"[tiab] OR "migração forçada"[tiab] OR "refugiado"[tiab] OR "deslocado interno"[tiab] OR "assentamento informal"[tiab] OR "crise ambiental"[tiab] OR "deslocamento"[tiab] OR "variabilidade climática"[tiab] OR "crise climática"[tiab] OR "relacionado ao clima"[tiab] OR "clima em mudança"[tiab] OR "emergência climática"[tiab] OR "choque climático"[tiab] OR "variabilidade do clima"[tiab] OR "tempo extremo"[tiab] OR "calor extremo"[tiab] OR "frio extremo"[tiab] OR "aumento da temperatura"[tiab] OR "onda de calor"[tiab] OR "tempestade"[tiab] OR "incêndio florestal"[tiab] OR "temporada de incêndios"[tiab] OR "deslocamento climático"[tiab] OR "migração climática"[tiab] OR "migração ambiental"[tiab] OR "degradação do ecossistema"[tiab] OR "crise ecológica"[tiab] OR "estresse ambiental"[tiab] OR "estresse climático"[tiab] OR "elevação do nível do mar"[tiab] OR "erosão costeira"[tiab] OR "desertificação"[tiab] OR "degelo glacial"[tiab] OR "perda de gelo"[tiab] OR "poluição"[tiab] **OR** "culture"[MeSH Terms] OR "traditional medicine practitioners"[MeSH Terms] OR "medicine, traditional"[MeSH Terms] OR "Faith Healing"[MeSH Terms] OR "Awareness"[MeSH Terms] OR "Knowledge"[MeSH Terms] OR "Attitude"[MeSH Terms] OR "Attitude to Health"[MeSH Terms] OR "Perception"[MeSH Terms] OR "Health Literacy"[MeSH Terms] OR "Social Isolation"[MeSH Terms] OR "Ostracism"[MeSH Terms] OR "Social Marginalization"[MeSH Terms] OR "Social Determinants of Health"[MeSH Terms] OR culture*[tiab] OR "cultural practices"[tiab] OR tradition*[tiab] OR ritual*[tiab] OR ceremony*[tiab] OR "social custom*"[tiab] OR heritage[tiab] OR "indigenous practice*"[tiab] OR folk*[tiab] OR folklore*[tiab] OR "cultural norm*"[tiab] OR value*[tiab] OR worldview*[tiab] OR "cultural identit*"[tiab] OR acculturation[tiab] OR enculturation[tiab] OR "cultural adaptation*"[tiab] OR ethnicit*[tiab] OR "social tradition*"[tiab] OR "traditional healer"[tiab] OR faith*[tiab] OR religion*[tiab] OR spirituality[tiab] OR "spiritual belief*"[tiab] OR worship*[tiab] OR theolog*[tiab] OR denomination*[tiab] OR "religious practice*"[tiab] OR "faith-based"[tiab] OR "divine belief*"[tiab] OR "religious identit*"[tiab] OR pilgrimage[tiab] OR sect*[tiab] OR healer*[tiab] OR knowledge*[tiab] OR attitude*[tiab] OR belief*[tiab] OR perception*[tiab] OR awareness[tiab] OR opinion*[tiab] OR practice*[tiab] OR KAP[tiab] OR literacy[tiab] OR "health literacy"[tiab] OR "information uptake"[tiab] OR understanding[tiab] OR "knowledge transfer"[tiab] OR "social exclusion"[tiab] OR "social marginalization"[tiab] OR "social isolation"[tiab] OR "marginalized communit*"[tiab] OR "social disadvantage*"[tiab] OR "excluded communit*"[tiab] OR "structural exclusion"[tiab] OR "systemic exclusion"[tiab] OR "social inequalit*"[tiab] OR discrimination[tiab] OR "social support network*"[tiab] OR "community connection*"[tiab] OR "social capital"[tiab] OR "access to technology"[tiab] OR "access to information"[tiab] OR "digital access"[tiab] OR "technology access"[tiab] OR "culture"[tiab] OR "pratiques culturelles"[tiab] OR "tradition"[tiab] OR "rituel"[tiab] OR "cérémonie"[tiab] OR "coutumes sociales"[tiab] OR "patrimoine"[tiab] OR "pratique autochtone"[tiab] OR "folklore"[tiab] OR "normes culturelles"[tiab] OR "valeurs"[tiab] OR "vision du monde"[tiab] OR "identité culturelle"[tiab] OR "acculturation"[tiab] OR "enculturation"[tiab] OR "adaptation culturelle"[tiab] OR "ethnicité"[tiab] OR "tradition sociale"[tiab] OR "guérisseur traditionnel"[tiab] OR "foi"[tiab] OR "religion"[tiab] OR "spiritualité"[tiab] OR "croyance spirituelle"[tiab] OR "culte"[tiab] OR "théologie"[tiab] OR "dénomination"[tiab] OR "pratique religieuse"[tiab] OR "fondé sur la foi"[tiab] OR "croyance divine"[tiab] OR "identité religieuse"[tiab] OR "pèlerinage"[tiab] OR "secte"[tiab] OR "guérisseur"[tiab] OR "connaissance"[tiab] OR "attitude"[tiab] OR "croyance"[tiab] OR "perception"[tiab] OR "sensibilisation"[tiab] OR "opinion"[tiab] OR "pratique"[tiab] OR "littératie en santé"[tiab] OR "acquisition d'information"[tiab] OR "compréhension"[tiab] OR "transfert de connaissances"[tiab] OR "exclusion sociale"[tiab] OR "marginalisation sociale"[tiab] OR "isolement social"[tiab] OR "communauté marginalisée"[tiab] OR "désavantage social"[tiab] OR "communauté exclue"[tiab] OR "exclusion structurelle"[tiab] OR "exclusion systémique"[tiab] OR "inégalités sociales"[tiab] OR "discrimination"[tiab] OR "réseau de soutien social"[tiab] OR "lien communautaire"[tiab] OR "capital social"[tiab] OR "accès à la technologie"[tiab] OR "accès à l'information"[tiab] OR "accès numérique"[tiab] OR "accès technologique"[tiab]
OR "cultura"[tiab] OR "prácticas culturales"[tiab] OR "tradición"[tiab] OR "ritual"[tiab] OR "ceremonia"[tiab] OR "costumbres sociales"[tiab] OR "patrimonio"[tiab] OR "práctica indígena"[tiab] OR "folclore"[tiab] OR "normas culturales"[tiab] OR "valores"[tiab] OR "cosmovisión"[tiab] OR "identidad cultural"[tiab] OR "aculturación"[tiab] OR "enculturación"[tiab] OR "adaptación cultural"[tiab] OR "etnicidad"[tiab] OR "tradición social"[tiab] OR "curandero tradicional"[tiab] OR "fe"[tiab] OR "religión"[tiab] OR "espiritualidad"[tiab] OR "creencia espiritual"[tiab] OR "adoración"[tiab] OR "teología"[tiab] OR "denominación"[tiab] OR "práctica religiosa"[tiab] OR "basado en la fe"[tiab] OR "creencia divina"[tiab] OR "identidad religiosa"[tiab] OR "peregrinación"[tiab] OR "secta"[tiab] OR "curandero"[tiab] OR "conocimiento"[tiab] OR "actitud"[tiab] OR "creencia"[tiab] OR "percepción"[tiab] OR "conciencia"[tiab] OR "opinión"[tiab] OR "práctica"[tiab] OR "alfabetización en salud"[tiab] OR "adquisición de información"[tiab] OR "comprensión"[tiab] OR "transferencia de conocimiento"[tiab] OR "exclusión social"[tiab] OR "marginalización social"[tiab] OR "aislamiento social"[tiab] OR "comunidad marginada"[tiab] OR "desventaja social"[tiab] OR "comunidad excluida"[tiab] OR "exclusión estructural"[tiab] OR "exclusión sistémica"[tiab] OR "desigualdades sociales"[tiab] OR "discriminación"[tiab] OR "red de apoyo social"[tiab] OR "conexión comunitaria"[tiab] OR "capital social"[tiab] OR "acceso a la tecnología"[tiab] OR "acceso a la información"[tiab] OR "acceso digital"[tiab] OR "acceso tecnológico"[tiab] OR "Kultur"[tiab] OR "kulturelle Praktiken"[tiab] OR "Tradition"[tiab] OR "Ritual"[tiab] OR "Zeremonie"[tiab] OR "soziale Bräuche"[tiab] OR "kulturelles Erbe"[tiab] OR "indigene Praxis"[tiab] OR "Volkskultur"[tiab] OR "kulturelle Normen"[tiab] OR "Werte"[tiab] OR "Weltanschauung"[tiab] OR "kulturelle Identität"[tiab] OR "Akkulturation"[tiab] OR "Enkulturation"[tiab] OR "kulturelle Anpassung"[tiab] OR "Ethnizität"[tiab] OR "soziale Tradition"[tiab] OR "traditioneller Heiler"[tiab] OR "Glaube"[tiab] OR "Religion"[tiab] OR "Spiritualität"[tiab] OR "spiritueller Glaube"[tiab] OR "Anbetung"[tiab] OR "Theologie"[tiab] OR "Konfession"[tiab] OR "religiöse Praxis"[tiab] OR "glaubensbasiert"[tiab] OR "göttlicher Glaube"[tiab] OR "religiöse Identität"[tiab] OR "Pilgerreise"[tiab] OR "Sekte"[tiab] OR "Heiler"[tiab] OR "Wissen"[tiab] OR "Einstellung"[tiab] OR "Glauben"[tiab] OR "Wahrnehmung"[tiab] OR "Bewusstsein"[tiab] OR "Meinung"[tiab] OR "Praxis"[tiab] OR "Gesundheitskompetenz"[tiab] OR "Informationsaufnahme"[tiab] OR "Verständnis"[tiab] OR "Wissensweitergabe"[tiab] OR "soziale Ausgrenzung"[tiab] OR "soziale Marginalisierung"[tiab] OR "soziale Isolation"[tiab] OR "marginalisierte Gemeinschaft"[tiab] OR "soziale Benachteiligung"[tiab] OR "ausgeschlossene Gemeinschaft"[tiab] OR "strukturelle Ausgrenzung"[tiab] OR "systemische Ausgrenzung"[tiab] OR "soziale Ungleichheiten"[tiab] OR "Diskriminierung"[tiab] OR "soziales Unterstützungsnetzwerk"[tiab] OR "gemeinschaftliche Verbindung"[tiab] OR "soziales Kapital"[tiab] OR "Zugang zu Technologie"[tiab] OR "Zugang zu Informationen"[tiab] OR "digitaler Zugang"[tiab] OR "technologischer Zugang"[tiab]
OR "cultura"[tiab] OR "práticas culturais"[tiab] OR "tradição"[tiab] OR "ritual"[tiab] OR "cerimônia"[tiab] OR "costumes sociais"[tiab] OR "patrimônio"[tiab] OR "prática indígena"[tiab] OR "folclore"[tiab] OR "normas culturais"[tiab] OR "valores"[tiab] OR "visão de mundo"[tiab] OR "identidade cultural"[tiab] OR "aculturação"[tiab] OR "enculturação"[tiab] OR "adaptação cultural"[tiab] OR "etnicidade"[tiab] OR "tradição social"[tiab] OR "curandeiro tradicional"[tiab] OR "fé"[tiab] OR "religião"[tiab] OR "espiritualidade"[tiab] OR "crença espiritual"[tiab] OR "adoração"[tiab] OR "teologia"[tiab] OR "denominação"[tiab] OR "prática religiosa"[tiab] OR "baseado na fé"[tiab] OR "crença divina"[tiab] OR "identidade religiosa"[tiab] OR "peregrinação"[tiab] OR "seita"[tiab] OR "curandeiro"[tiab] OR "conhecimento"[tiab] OR "atitude"[tiab] OR "crença"[tiab] OR "percepção"[tiab] OR "consciência"[tiab] OR "opinião"[tiab] OR "prática"[tiab] OR "alfabetização em saúde"[tiab] OR "obtenção de informação"[tiab] OR "compreensão"[tiab] OR "transferência de conhecimento"[tiab] OR "exclusão social"[tiab] OR "marginalização social"[tiab] OR "isolamento social"[tiab] OR "comunidade marginalizada"[tiab] OR "desvantagem social"[tiab] OR "comunidade excluída"[tiab] OR "exclusão estrutural"[tiab] OR "exclusão sistêmica"[tiab] OR "desigualdades sociais"[tiab] OR "discriminação"[tiab] OR "rede de apoio social"[tiab] OR "conexão comunitária"[tiab] OR "capital social"[tiab] OR "acesso à tecnologia"[tiab] OR "acesso à informação"[tiab] OR "acesso digital"[tiab] OR "acesso tecnológico"[tiab]

SEARCH STRATEGY IN PUBMED STYLE

((("Buruli Ulcer"[Mesh] OR "Mycobacterium ulcerans"[Mesh] OR "Leishmaniasis, Cutaneous"[Mesh] OR "Leishmaniasis, Mucocutaneous"[Mesh] OR "Leishmaniasis, Diffuse Cutaneous"[Mesh] OR "Leprosy"[Mesh] OR "Mycobacterium leprae"[Mesh] OR "Filariasis, Lymphatic"[Mesh] OR "Wuchereria bancrofti"[Mesh] OR "Brugia malayi"[Mesh] OR "Chromoblastomycosis"[Mesh] OR "Noma"[Mesh] OR "Mycetoma"[Mesh] OR "Actinomycetoma"[Mesh] OR "Eumycetoma"[Mesh] OR "Sporotrichosis"[Mesh] OR "Onchocerciasis"[Mesh] OR "Onchocerca volvulus"[Mesh] OR "Scabies"[Mesh] OR "Tungiasis"[Mesh] OR "Yaws"[Mesh] OR "Treponema pallidum"[Mesh] OR "Buruli ulcer"[tiab] OR "Mycobacterium ulcerans"[tiab] OR "buruli ulcers"[tiab] OR "cutaneous leishmaniasis"[tiab] OR "American Tegumentary Leishmaniasis"[tiab] OR "post-kala-azar dermal leishmaniasis"[tiab] OR "PKDL"[tiab] OR "dermal leishmaniasis"[tiab] OR "localized cutaneous leishmaniasis"[tiab] OR "mucocutaneous leishmaniasis"[tiab] OR "mucosal leishmaniasis"[tiab] OR "diffuse cutaneous leishmaniasis"[tiab] OR "leishmaniasis recidivans"[tiab] OR "Leprosy"[tiab] OR "Mycobacterium leprae"[tiab] OR "multibacillary leprosy"[tiab] OR "lepromatous leprosy"[tiab] OR "paucibacillary leprosy"[tiab] OR "borderline leprosy"[tiab] OR "tuberculoid leprosy"[tiab] OR "mycobacterium lepromatosis"[tiab] OR "lymphatic filariasis"[tiab] OR "elephantiasis"[tiab] OR "Wuchereria bancrofti"[tiab] OR "Brugia malayi"[tiab] OR "Brugia timori"[tiab] OR "Brugia pahangi"[tiab] OR "bancrofti microfilaria"[tiab] OR "bancroftian filariasis"[tiab] OR "brugian filariasis"[tiab] OR "chromoblastomycosis"[tiab] OR "chromoblastomycosis infection"[tiab] OR "dematiaceous fungi"[tiab] OR "Fonsecaea"[tiab] OR "Cladophialophora"[tiab] OR "Rhinocladiella"[tiab] OR "Phialophora"[tiab] OR "Exophiala"[tiab] OR "exophiala species"[tiab] OR "exophiala dermatitidis"[tiab] OR "Noma"[tiab] OR "Mycetoma"[tiab] OR "Maduromycosis"[tiab] OR "Madura Foot"[tiab] OR "Actinomycetoma"[tiab] OR "Eumycetoma"[tiab] OR "Madurella"[tiab] OR "madurella mycetomatis"[tiab] OR "sporotrichosis"[tiab] OR "Sporothrix schenckii"[tiab] OR "Sporothrix"[tiab] OR "Sporothrix brasiliensis"[tiab] OR "Sporothrix globosa"[tiab] OR "River blindness"[tiab] OR "onchocerciasis"[tiab] OR "Onchocerca volvulus"[tiab] OR "Onchocerca cervicalis"[tiab] OR "Onchocerca reticulata"[tiab] OR "Onchocerca gibsoni"[tiab] OR "onchocerca ochengi"[tiab] OR "Scabies"[tiab] OR "Sarcoptes scabiei"[tiab] OR "Sarcoptes scabiei var hominis"[tiab] OR "sarcoptes mite"[tiab] OR "sarcoptic itch"[tiab] OR "sarcoptic acariasis"[tiab] OR "mange"[tiab] OR "crusted scabies"[tiab] OR "scabiei mites"[tiab] OR "Tungiasis"[tiab] OR "Tunga penetrans"[tiab] OR "yaws"[tiab] OR "Treponema pallidum pertenue"[tiab] OR "Treponema pallidum subsp. pertenue"[tiab] OR "Treponema pallidum subspecies pertenue"[tiab] OR "endemic treponematosis"[tiab] OR "endemic treponematoses"[tiab] OR "treponematos*"[tiab] OR "framboesia"[tiab] OR "Patek"[tiab] OR "Parangi"[tiab] OR "Bouba"[tiab] OR "Coco yaws"[tiab] OR "Goundou"[tiab]) AND ("One Health"[MeSH Terms] OR "Zoonoses"[MeSH Terms] OR "Zoonotic Diseases"[MeSH Terms] OR "Animals, Domestic"[MeSH Terms] OR "Animals, Wild"[MeSH Terms] OR "Human-Animal Bond"[MeSH Terms] OR "Pets"[MeSH Terms] OR "Rodentia"[MeSH Terms] OR "Bats"[MeSH Terms] OR "Chiroptera"[MeSH Terms] OR "Livestock"[MeSH Terms] OR "Agriculture"[MeSH Terms] OR "Organic Agriculture"[MeSH Terms] OR "Gardening"[MeSH Terms] OR ("one"[All Fields] AND "health"[All Fields]) OR "one health"[All Fields] OR "one health"[tiab] OR "Human-Animal Interaction"[MeSH Terms] OR "animals, wild"[MeSH Terms] OR "animals, domestic"[MeSH Terms] OR "Organic Agriculture"[MeSH Terms] OR "Gardening"[MeSH Terms] OR "Agriculture"[MeSH Terms] "One Health"[tiab] OR "Zoonotic Disease*"[tiab] OR "Bats"[tiab] OR "Chiroptera"[tiab] OR "Organic Agriculture"[tiab] OR ("human*"[tiab] AND "animal*"[tiab]) OR ("human*"[tiab] AND "environment*"[tiab]) OR ("anima*"[tiab] AND "environment*"[tiab]) OR "human-animal interaction*"[tiab] OR "biophilia"[tiab] OR "nature connection*"[tiab] OR "ecotherapy*"[tiab] OR "human–environment interaction*"[tiab] OR "environmental exposure*"[tiab] OR "pet ownership*"[tiab] OR "companion animal*"[tiab] OR "service animal*"[tiab] OR "animal companionship*"[tiab] OR "wildlife interaction*"[tiab] OR "gardening"[tiab] OR "horticultural"[tiab] OR "food source"[tiab] OR "food preparation"[tiab] OR "reservoir"[tiab] OR "intermediate host"[tiab] OR "zoono*"[tiab] OR "anthropozoonosis"[tiab] OR "domestic animal*"[tiab] OR "peridomestic"[tiab] OR "sylvatic"[tiab] OR "livestock"[tiab] OR "subsistence farming"[tiab] OR "bats"[tiab] OR "rodents"[tiab] OR "rock hyrax"[tiab] OR "chicken*"[tiab] OR "cow"[tiab] OR "cows"[tiab] OR "goat*"[tiab] OR "crop*"[tiab] OR "une seule santé"[tiab] OR "une santé seule"[tiab] OR "santé unique"[tiab] OR "santé humaine et animale"[tiab] OR "interaction homme-animal"[tiab] OR "réservoir"[tiab] OR "hôte intermédiaire"[tiab] OR "zoonose"[tiab] OR "zoonose anthropozoonotique"[tiab] OR "animal domestique"[tiab] OR "péridomestique"[tiab] OR "sylvatique"[tiab] OR "élevage"[tiab] OR "agriculture de subsistance"[tiab] OR "chauves-souris"[tiab] OR "rongeurs"[tiab] OR "daman des rochers"[tiab] OR "una sola salud"[tiab] OR "salud única"[tiab] OR "interacción humano-animal"[tiab] OR "reservorio"[tiab] OR "huésped intermediario"[tiab] OR "zoonosis"[tiab] OR "antropozoonosis"[tiab] OR "animal doméstico"[tiab] OR "peridoméstico"[tiab] OR "silvático"[tiab] OR "ganado"[tiab] OR "agricultura de subsistencia"[tiab] OR "murciélagos"[tiab] OR "roedores"[tiab] OR "daman de las rocas"[tiab] OR "eine gesundheit"[tiab] OR "mensch-tier-interaktion"[tiab] OR "tier-mensch-interaktion"[tiab] OR "wirtstier"[tiab] OR "zwischenwirt"[tiab] OR "zoonose"[tiab] OR "anthropozoonose"[tiab] OR "haustier"[tiab] OR "peridomestisch"[tiab] OR "sylvatisch"[tiab] OR "vieh"[tiab] OR "subsistenzlandwirtschaft"[tiab] OR "fledermäuse"[tiab] OR "nagetiere"[tiab] OR "klippschliefer"[tiab] OR "uma só saúde"[tiab] OR "saúde única"[tiab] OR "interação humano-animal"[tiab] OR "reservatório"[tiab] OR "hospedeiro intermediário"[tiab] OR "zoonose"[tiab] OR "antropozoonose"[tiab] OR "animal doméstico"[tiab] OR "peridoméstico"[tiab] OR "silvático"[tiab] OR "gado"[tiab] OR "agricultura de subsistência"[tiab] OR "morcegos"[tiab] OR "roedores"[tiab] OR "procávia-das-rochas"[tiab])) AND ("mental health"[MeSH Terms] OR "Psychological Well-Being"[MeSH Terms] OR "Emotions"[MeSH Terms] OR "Quality of Life"[MeSH Terms] OR "mental health"[tiab] OR "psychological well-being"[tiab] OR "wellbeing"[tiab] OR "well-being"[tiab] OR "emotion*"[tiab] OR "affect*"[tiab] OR "emotional regulation*"[tiab] OR "mental wellness"[tiab] OR psych*[tiab] OR "quality of life"[tiab] OR "santé mentale"[tiab] OR "bien-être psychologique"[tiab] OR "bien-être"[tiab] OR "émotions"[tiab] OR "régulation émotionnelle"[tiab] OR "bien-être mental"[tiab] OR "qualité de vie"[tiab] OR "salud mental"[tiab] OR "bienestar psicológico"[tiab] OR "bienestar"[tiab] OR "emociones"[tiab] OR "regulación emocional"[tiab] OR "bienestar mental"[tiab] OR "calidad de vida"[tiab] OR "psychische Gesundheit"[tiab] OR "psychisches Wohlbefinden"[tiab] OR "Wohlbefinden"[tiab] OR "Emotionen"[tiab] OR "emotionale Regulierung"[tiab] OR "psychisches Wohlsein"[tiab] OR "Lebensqualität"[tiab] OR "saúde mental"[tiab] OR "bem-estar psicológico"[tiab] OR "bem-estar"[tiab] OR "emoções"[tiab] OR "regulação emocional"[tiab] OR "bem-estar mental"[tiab] OR "qualidade de vida"[tiab] OR "Mental Disorders"[MeSH] OR "Psychopathology"[MeSH] OR "psychopatholog*"[tiab] OR "mental illness*"[tiab] OR "psychiatric disorder*"[tiab] OR "psychological disorder*"[tiab] OR "emotional disorder*"[tiab] OR "behavioral disorder*"[tiab] OR "mental disorder*"[tiab] OR "internalizing disorder*"[tiab] OR "externalizing disorder*"[tiab] OR "psychosis"[tiab] OR "neurosis"[tiab] OR "depression"[tiab] OR "anxiety"[tiab] OR "bipolar disorder"[tiab] OR "schizophrenia"[tiab] OR "PTSD"[tiab] OR "OCD"[tiab] OR "psychopathologie"[tiab] OR "trouble mental"[tiab] OR "maladie mentale"[tiab] OR "trouble psychiatrique"[tiab] OR "trouble émotionnel"[tiab] OR "psychose"[tiab] OR "névrose"[tiab] OR "dépression"[tiab] OR "anxiété"[tiab] OR "schizophrénie"[tiab] OR "psicopatología"[tiab] OR "enfermedad mental"[tiab] OR "trastorno psiquiátrico"[tiab] OR "trastorno psicológico"[tiab] OR "psicosis"[tiab] OR "neurosis"[tiab] OR "depresión"[tiab] OR "ansiedad"[tiab] OR "esquizofrenia"[tiab] OR "Psychopathologie"[tiab] OR "psychische Störung"[tiab] OR "Geisteskrankheit"[tiab] OR "psychotische Störung"[tiab] OR "Depression"[tiab] OR "Angst"[tiab] OR "Schizophrenie"[tiab] OR "psicopatologia"[tiab] OR "transtorno mental"[tiab] OR "doença mental"[tiab] OR "transtorno psiquiátrico"[tiab] OR "psicose"[tiab] OR "neurose"[tiab] OR "depressão"[tiab] OR "ansiedade"[tiab] OR "esquizofrenia"[tiab] OR "Social Stigma"[Mesh] OR "Social Discrimination"[Mesh] OR "Shame"[Mesh] OR "Prejudice"[Mesh] OR "stigma"[tiab] OR "stigmatization"[tiab] OR "discrimination"[tiab] OR "prejudice"[tiab] OR "shame"[tiab] OR "self stigma"[tiab] OR "public stigma"[tiab] OR "perceived stigma"[tiab] OR "enacted stigma"[tiab] OR "stigmatisation"[tiab] OR "discrimination sociale"[tiab] OR "préjugé"[tiab] OR "honte"[tiab] OR "estigmatización"[tiab] OR "discriminación social"[tiab] OR "prejuicio"[tiab] OR "vergüenza"[tiab] OR "Stigmatisierung"[tiab] OR "soziale Diskriminierung"[tiab] OR "Vorurteil"[tiab] OR "Scham"[tiab] OR "estigmatização"[tiab] OR "discriminação social"[tiab] OR "preconceito"[tiab] OR "vergonha"[tiab] OR "Disabled Persons"[Mesh] OR "Disability Evaluation"[Mesh] OR "Developmental Disabilities"[Mesh] OR "Intellectual Disability"[Mesh] OR "Disability Discrimination"[Mesh] OR "Disability Studies"[Mesh] OR "Persons with Disabilities"[Mesh] OR "Persons with Intellectual Disabilities"[Mesh] OR "disabilit*"[tiab] OR "impairment*"[tiab] OR "functional limitation*"[tiab] OR "developmental delay*"[tiab] OR "cognitive impairment*"[tiab] OR "mobility limitation*"[tiab] OR "learning disabilit*"[tiab] OR "handicap"[tiab] OR "incapacité"[tiab] OR "déficience*"[tiab] OR "limitation fonctionnelle"[tiab] OR "retard de développement"[tiab] OR "trouble cognitif"[tiab] OR "limitation motrice"[tiab] OR "trouble de l’apprentissage"[tiab] OR "discapacidad"[tiab] OR "impedimento"[tiab] OR "limitación funcional"[tiab] OR "retraso del desarrollo"[tiab] OR "deterioro cognitivo"[tiab] OR "limitación motora"[tiab] OR "dificultad de aprendizaje"[tiab] OR "Behinderung"[tiab] OR "Beeinträchtigung"[tiab] OR "funktionale Einschränkung"[tiab] OR "Entwicklungsverzögerung"[tiab] OR "kognitive Beeinträchtigung"[tiab] OR "Mobilitätseinschränkung"[tiab] OR "Lernbehinderung"[tiab] OR "deficiência"[tiab] OR "incapacidade"[tiab] OR "limitação funcional"[tiab] OR "atraso no desenvolvimento"[tiab] OR "comprometimento cognitivo"[tiab] OR "limitação de mobilidade"[tiab] OR "dificuldade de aprendizagem"[tiab])) AND ("Sex Factors"[MeSH] OR "Gender Identity"[MeSH] OR "Sex Characteristics"[MeSH] OR "Age Factors"[MeSH] OR "Ethnicity"[MeSH] OR "Population Density"[MeSH] OR "Cultural Diversity"[MeSH] OR "Longevity"[MeSH] OR "Life Expectancy"[MeSH] OR "Survival Rate"[MeSH] OR "Survival Analysis"[MeSH] OR "gender*"[tiab] OR "sex"[tiab] OR "sex factor*"[tiab] OR "gender identit*"[tiab] OR "sex characteristic*"[tiab] OR "sex difference*"[tiab] OR "gender-based"[tiab] OR "Age Factors"[tiab] OR "age"[tiab] OR "adolescent"[tiab] OR "adult"[tiab] OR "aged"[tiab] OR "geriatric"[tiab] OR "Ethnicity"[tiab] OR "Ethnic Group*"[tiab] OR "ethnicity"[tiab] OR "race"[tiab] OR "Population Density"[tiab] OR "Multiculturalism"[tiab] OR "diversity"[tiab] OR "community diversity"[tiab] OR "Longevity"[tiab] OR "longevity"[tiab] OR "Life Expectancy"[tiab] OR "life expectancy"[tiab] OR "Survival Rate"[tiab] OR "survival"[tiab] OR "Survival Analysis"[tiab] OR "abstinence"[tiab] OR "marriageability"[tiab] OR "sexual interest"[tiab] OR "sexuality"[tiab] OR "autochthonous population"[tiab] OR "indigenous population"[tiab] OR "sexe"[tiab] OR "genre"[tiab] OR "identité de genre"[tiab] OR "caractéristiques sexuelles"[tiab] OR "différences sexuelles"[tiab] OR "basé sur le genre"[tiab] OR "facteurs liés à l'âge"[tiab] OR "âge"[tiab] OR "adolescent"[tiab] OR "adulte"[tiab] OR "âgé"[tiab] OR "gériatrique"[tiab] OR "ethnicité"[tiab] OR "groupe ethnique"[tiab] OR "densité de population"[tiab] OR "multiculturalisme"[tiab] OR "diversité"[tiab] OR "diversité communautaire"[tiab] OR "longévité"[tiab] OR "espérance de vie"[tiab] OR "taux de survie"[tiab] OR "analyse de survie"[tiab] OR "abstinence"[tiab] OR "aptitude au mariage"[tiab] OR "intérêt sexuel"[tiab] OR "sexualité"[tiab] OR "population autochtone"[tiab] OR "population indigène"[tiab] OR "sexo"[tiab] OR "género"[tiab] OR "identidad de género"[tiab] OR "características sexuales"[tiab] OR "diferencias sexuales"[tiab] OR "basado en género"[tiab] OR "factores de edad"[tiab] OR "edad"[tiab] OR "adolescente"[tiab] OR "adulto"[tiab] OR "anciano"[tiab] OR "geriátrico"[tiab] OR "etnicidad"[tiab] OR "grupo étnico"[tiab] OR "densidad de población"[tiab] OR "multiculturalismo"[tiab] OR "diversidad"[tiab] OR "diversidad comunitaria"[tiab] OR "longevidad"[tiab] OR "esperanza de vida"[tiab] OR "tasa de supervivencia"[tiab] OR "análisis de supervivencia"[tiab] OR "abstinencia"[tiab] OR "aptitud matrimonial"[tiab] OR "interés sexual"[tiab] OR "sexualidad"[tiab] OR "población autóctona"[tiab] OR "población indígena"[tiab] OR "Geschlecht"[tiab] OR "Gender"[tiab] OR "Geschlechtsidentität"[tiab] OR "geschlechtliche Merkmale"[tiab] OR "Geschlechtsunterschiede"[tiab] OR "geschlechtsbasiert"[tiab] OR "Altersfaktoren"[tiab] OR "Alter"[tiab] OR "Jugendlicher"[tiab] OR "Erwachsener"[tiab] OR "älter"[tiab] OR "geriatrisch"[tiab] OR "Ethnizität"[tiab] OR "ethnische Gruppe"[tiab] OR "Bevölkerungsdichte"[tiab] OR "Multikulturalismus"[tiab] OR "Vielfalt"[tiab] OR "gemeinschaftliche Vielfalt"[tiab] OR "Langlebigkeit"[tiab] OR "Lebenserwartung"[tiab] OR "Überlebensrate"[tiab] OR "Überlebensanalyse"[tiab] OR "Abstinenz"[tiab] OR "Heiratsfähigkeit"[tiab] OR "sexuelles Interesse"[tiab] OR "Sexualität"[tiab] OR "autochthone Bevölkerung"[tiab] OR "indigene Bevölkerung"[tiab] OR "sexo"[tiab] OR "gênero"[tiab] OR "identidade de gênero"[tiab] OR "características sexuais"[tiab] OR "diferenças sexuais"[tiab] OR "baseado no gênero"[tiab] OR "fatores etários"[tiab] OR "idade"[tiab] OR "adolescente"[tiab] OR "adulto"[tiab] OR "idoso"[tiab] OR "geriátrico"[tiab] OR "etnicidade"[tiab] OR "grupo étnico"[tiab] OR "densidade populacional"[tiab] OR "multiculturalismo"[tiab] OR "diversidade"[tiab] OR "diversidade comunitária"[tiab] OR "longevidade"[tiab] OR "expectativa de vida"[tiab] OR "taxa de sobrevivência"[tiab] OR "análise de sobrevivência"[tiab] OR "abstinência"[tiab] OR "aptidão para o casamento"[tiab] OR "interesse sexual"[tiab] OR "sexualidade"[tiab] OR "população autóctone"[tiab] OR "população indígena"[tiab] OR "economics"[MeSH Subheading] OR "income"[MeSH Terms] OR "financial stress"[MeSH Terms] OR "Medically Uninsured"[Mesh] OR "Malnutrition"[Mesh] OR "Food Security"[Mesh] OR "Poverty"[MeSH] OR "Socioeconomic Factors"[MeSH] OR "economic*"[tiab] OR "cost*"[tiab] OR "financial*"[tiab] OR "affordability*"[tiab] OR "income*"[tiab] OR "socioeconomic*"[tiab] OR "economic barrier*"[tiab] OR "cost-benefit"[tiab] OR "economic burden*"[tiab] OR "financial burden"[tiab] OR "economic impact"[tiab] OR "cost* of illness"[tiab] OR "economic strain"[tiab] OR "financial hardship"[tiab] OR "povert*"[tiab] OR "low-income"[tiab] OR "low income"[tiab] OR "economic hardship*"[tiab] OR "financial hardship*"[tiab] OR "economic disadvantag*"[tiab] OR "socioeconomic status"[tiab] OR "socioeconomic deprivation"[tiab] OR "income inequalit*"[tiab] OR "social class"[tiab] OR "education"[tiab] OR "educational attainment"[tiab] OR "literacy"[tiab] OR "food security"[tiab] OR "food insecurity"[tiab] OR "nutritional deprivation"[tiab] OR "deprivation"[tiab] OR "housing instability"[tiab] OR "basic needs"[tiab] OR "inequities"[tiab] OR "debt*"[tiab] OR "asset*"[tiab] OR "cash*"[tiab] OR "financial strain"[tiab] OR "economic recession*"[tiab] OR "macroeconomic"[tiab] OR "livelihood*"[tiab] OR "unemploy*"[tiab] OR "employ*"[tiab] OR "financial stress*"[tiab] OR "uninsured"[tiab] OR "insur*"[tiab] OR "malnutrition"[tiab] OR "food insecurity"[tiab] OR "food security"[tiab] OR "Transportation cost*"[tiab] OR "economic disparity*"[tiab] OR "économie"[tiab] OR "revenu"[tiab] OR "stress financier"[tiab] OR "coût"[tiab] OR "financier"[tiab] OR "accessibilité financière"[tiab] OR "revenus faibles"[tiab] OR "socioéconomique"[tiab] OR "barrière économique"[tiab] OR "fardeau économique"[tiab] OR "fardeau financier"[tiab] OR "impact économique"[tiab] OR "coût de la maladie"[tiab] OR "pression économique"[tiab] OR "privation économique"[tiab] OR "inégalités de revenus"[tiab] OR "classe sociale"[tiab] OR "éducation"[tiab] OR "niveau d'instruction"[tiab] OR "alphabétisation"[tiab] OR "sécurité alimentaire"[tiab] OR "insécurité alimentaire"[tiab] OR "privation nutritionnelle"[tiab] OR "privation"[tiab] OR "instabilité du logement"[tiab] OR "besoins de base"[tiab] OR "inégalités"[tiab] OR "economía"[tiab] OR "ingresos"[tiab] OR "estrés financiero"[tiab] OR "costo"[tiab] OR "financiero"[tiab] OR "asequibilidad"[tiab] OR "bajos ingresos"[tiab] OR "socioeconómico"[tiab] OR "barrera económica"[tiab] OR "carga económica"[tiab] OR "carga financiera"[tiab] OR "impacto económico"[tiab] OR "costo de la enfermedad"[tiab] OR "tensión económica"[tiab] OR "privación económica"[tiab] OR "desigualdad de ingresos"[tiab] OR "clase social"[tiab] OR "educación"[tiab] OR "nivel educativo"[tiab] OR "alfabetización"[tiab] OR "seguridad alimentaria"[tiab] OR "inseguridad alimentaria"[tiab] OR "privación nutricional"[tiab] OR "privación"[tiab] OR "inestabilidad habitacional"[tiab] OR "necesidades básicas"[tiab] OR "desigualdades"[tiab] OR "Wirtschaft"[tiab] OR "Einkommen"[tiab] OR "finanzieller Stress"[tiab] OR "Kosten"[tiab] OR "finanziell"[tiab] OR "Bezahlbarkeit"[tiab] OR "niedriges Einkommen"[tiab] OR "sozioökonomisch"[tiab] OR "wirtschaftliche Barriere"[tiab] OR "wirtschaftliche Belastung"[tiab] OR "finanzielle Belastung"[tiab] OR "wirtschaftlicher Einfluss"[tiab] OR "Krankheitskosten"[tiab] OR "wirtschaftlicher Druck"[tiab] OR "wirtschaftliche Notlage"[tiab] OR "Einkommensungleichheit"[tiab] OR "soziale Klasse"[tiab] OR "Bildung"[tiab] OR "Bildungsstand"[tiab] OR "Alphabetisierung"[tiab] OR "Ernährungssicherheit"[tiab] OR "Ernährungsunsicherheit"[tiab] OR "ernährungsbedingte Notlage"[tiab] OR "Entbehrung"[tiab] OR "Wohnungsinstabilität"[tiab] OR "Grundbedürfnisse"[tiab] OR "Ungleichheiten"[tiab] OR "economia"[tiab] OR "renda"[tiab] OR "estresse financeiro"[tiab] OR "custo"[tiab] OR "financeiro"[tiab] OR "acessibilidade financeira"[tiab] OR "baixa renda"[tiab] OR "socioeconômico"[tiab] OR "barreira econômica"[tiab] OR "fardo econômico"[tiab] OR "fardo financeiro"[tiab] OR "impacto econômico"[tiab] OR "custo da doença"[tiab] OR "pressão econômica"[tiab] OR "privação econômica"[tiab] OR "desigualdade de renda"[tiab] OR "classe social"[tiab] OR "educação"[tiab] OR "escolaridade"[tiab] OR "alfabetização"[tiab] OR "segurança alimentar"[tiab] OR "insegurança alimentar"[tiab] OR "privação nutricional"[tiab] OR "privação"[tiab] OR "instabilidade habitacional"[tiab] OR "necessidades básicas"[tiab] OR "desigualdades"[tiab] OR "Residence Characteristics"[Mesh] OR "Neighborhood Characteristics"[Mesh] OR "Community Health Centers"[Mesh] OR "Housing Quality"[Mesh] OR "Housing"[Mesh] OR "Accident Prevention"[Mesh] OR "Social Deprivation"[Mesh] OR "neighborhood*"[tiab] OR "local area*"[tiab] OR "setting*"[tiab] OR "housing quality*"[tiab] OR "overcrowd*"[tiab] OR "disinvest*"[tiab] OR "security*"[tiab] OR "safety*"[tiab] OR "public facility*"[tiab] OR "recreation*"[tiab] OR "park*"[tiab] OR "urban"[tiab] OR "rural"[tiab] OR "peri urban"[tiab] OR "quartier"[tiab] OR "zone locale"[tiab] OR "environnement"[tiab] OR "qualité du logement"[tiab] OR "surpopulation"[tiab] OR "désinvestissement"[tiab] OR "sécurité"[tiab] OR "sûreté"[tiab] OR "infrastructure publique"[tiab] OR "loisirs"[tiab] OR "espace vert"[tiab] OR "parc"[tiab] OR "urbain"[tiab] OR "rural"[tiab] OR "périurbain"[tiab] OR "vecindario"[tiab] OR "zona local"[tiab] OR "entorno"[tiab] OR "calidad de la vivienda"[tiab] OR "hacinamiento"[tiab] OR "desinversión"[tiab] OR "seguridad"[tiab] OR "protección"[tiab] OR "instalaciones públicas"[tiab] OR "recreación"[tiab] OR "espacio verde"[tiab] OR "parque"[tiab] OR "urbano"[tiab] OR "rural"[tiab] OR "periurbano"[tiab] OR "Nachbarschaft"[tiab] OR "lokales Gebiet"[tiab] OR "Umgebung"[tiab] OR "Wohnqualität"[tiab] OR "Überbelegung"[tiab] OR "Desinvestition"[tiab] OR "Sicherheit"[tiab] OR "Schutz"[tiab] OR "öffentliche Einrichtung"[tiab] OR "Freizeit"[tiab] OR "Grünfläche"[tiab] OR "Park"[tiab] OR "städtisch"[tiab] OR "ländlich"[tiab] OR "periurban"[tiab] OR "vizinhança"[tiab] OR "área local"[tiab] OR "ambiente"[tiab] OR "qualidade da habitação"[tiab] OR "superlotação"[tiab] OR "desinvestimento"[tiab] OR "segurança"[tiab] OR "proteção"[tiab] OR "instalações públicas"[tiab] OR "recreação"[tiab] OR "espaço verde"[tiab] OR "parque"[tiab] OR "urbano"[tiab] OR "rural"[tiab] OR "periurbano"[tiab] OR "Natural Disasters"[Mesh] OR "Climate Change"[Mesh] OR "Environmental Pollution"[Mesh] OR "Armed Conflicts"[Mesh] OR "Human Migration"[Mesh] OR "Global Warming"[MeSH] OR "Extreme Weather"[MeSH] OR "Extreme Cold"[MeSH] OR "Extreme Heat"[MeSH] OR "Environmental Exposure"[MeSH] OR "Climate"[MeSH] OR "Weather"[MeSH] OR "Droughts"[MeSH] OR "Floods"[MeSH] OR "Temperature"[MeSH] OR "Greenhouse Effect"[MeSH] OR "Air Pollution"[MeSH] OR "Environmental Health"[MeSH] OR "Fires"[MeSH] OR "Wildfires"[MeSH] OR "Accidents, Occupational"[Mesh] OR "industrial disaster*"[tiab] OR "chemical spill*"[tiab] OR "oil spill*"[tiab] OR "natural disaster*"[tiab] OR "earthquake*"[tiab] OR "flood*"[tiab] OR "hurricane*"[tiab] OR "cyclone*"[tiab] OR "wildfire*"[tiab] OR "drought*"[tiab] OR "landslide*"[tiab] OR "climate change*"[tiab] OR "global warming*"[tiab] OR "environmental hazard*"[tiab] OR "environmental degradation*"[tiab] OR "war"[tiab] OR "conflict*"[tiab] OR "forced migration*"[tiab] OR "refugee*"[tiab] OR "internally displaced person*"[tiab] OR "informal settlement*"[tiab] OR "environmental cris*"[tiab] OR "displacement*"[tiab] OR "climate variabilit*"[tiab] OR "climate crisis"[tiab] OR "climate-related"[tiab] OR "changing climate"[tiab] OR "climate emergency"[tiab] OR "global heating"[tiab] OR "climate shock*"[tiab] OR "weather variabilit*"[tiab] OR "extreme weather"[tiab] OR "extreme heat"[tiab] OR "extreme cold"[tiab] OR "temperature rise"[tiab] OR "temperature increase"[tiab] OR "heatwave*"[tiab] OR "storm*"[tiab] OR "bushfire*"[tiab] OR "fire season"[tiab] OR "climate-induced displacement"[tiab] OR "climate migration"[tiab] OR "environmental migration"[tiab] OR "ecosystem degradation"[tiab] OR "ecological cris*"[tiab] OR "climate hazard*"[tiab] OR "environmental stress*"[tiab] OR "climate stress*"[tiab] OR "sea level rise"[tiab] OR "coastal erosion"[tiab] OR "desertification"[tiab] OR "glacial melt"[tiab] OR "ice loss"[tiab] OR "pollution"[tiab] OR "catastrophe naturelle"[tiab] OR "tremblement de terre"[tiab] OR "inondation"[tiab] OR "ouragan"[tiab] OR "cyclone"[tiab] OR "feu de forêt"[tiab] OR "sécheresse"[tiab] OR "glissement de terrain"[tiab] OR "changement climatique"[tiab] OR "réchauffement climatique"[tiab] OR "risque environnemental"[tiab] OR "dégradation environnementale"[tiab] OR "guerre"[tiab] OR "conflit"[tiab] OR "migration forcée"[tiab] OR "réfugié"[tiab] OR "personne déplacée intérieure"[tiab] OR "quartier informel"[tiab] OR "crise environnementale"[tiab] OR "déplacement"[tiab] OR "variabilité climatique"[tiab] OR "crise climatique"[tiab] OR "lié au climat"[tiab] OR "climat changeant"[tiab] OR "urgence climatique"[tiab] OR "choc climatique"[tiab] OR "variabilité météorologique"[tiab] OR "chaleur extrême"[tiab] OR "froid extrême"[tiab] OR "hausse de température"[tiab] OR "augmentation de température"[tiab] OR "vague de chaleur"[tiab] OR "tempête"[tiab] OR "feu de brousse"[tiab] OR "saison des incendies"[tiab] OR "déplacement climatique"[tiab] OR "migration climatique"[tiab] OR "migration environnementale"[tiab] OR "dégradation de l'écosystème"[tiab] OR "crise écologique"[tiab] OR "stress environnemental"[tiab] OR "stress climatique"[tiab] OR "élévation du niveau de la mer"[tiab] OR "érosion côtière"[tiab] OR "désertification"[tiab] OR "fonte des glaciers"[tiab] OR "perte de glace"[tiab] OR "desastre natural"[tiab] OR "terremoto"[tiab] OR "inundación"[tiab] OR "huracán"[tiab] OR "ciclón"[tiab] OR "incendio forestal"[tiab] OR "sequía"[tiab] OR "deslizamiento de tierra"[tiab] OR "cambio climático"[tiab] OR "calentamiento global"[tiab] OR "peligro ambiental"[tiab] OR "degradación ambiental"[tiab] OR "guerra"[tiab] OR "conflicto"[tiab] OR "migración forzada"[tiab] OR "refugiado"[tiab] OR "desplazado interno"[tiab] OR "asentamiento informal"[tiab] OR "crisis ambiental"[tiab] OR "desplazamiento"[tiab] OR "variabilidad climática"[tiab] OR "crisis climática"[tiab] OR "relacionado con el clima"[tiab] OR "clima cambiante"[tiab] OR "emergencia climática"[tiab] OR "choque climático"[tiab] OR "variabilidad del clima"[tiab] OR "clima extremo"[tiab] OR "calor extremo"[tiab] OR "frío extremo"[tiab] OR "aumento de temperatura"[tiab] OR "ola de calor"[tiab] OR "tormenta"[tiab] OR "incendio forestal"[tiab] OR "temporada de incendios"[tiab] OR "desplazamiento climático"[tiab] OR "migración climática"[tiab] OR "migración ambiental"[tiab] OR "degradación del ecosistema"[tiab] OR "crisis ecológica"[tiab] OR "estrés ambiental"[tiab] OR "estrés climático"[tiab] OR "aumento del nivel del mar"[tiab] OR "erosión costera"[tiab] OR "desertificación"[tiab] OR "derretimiento de glaciares"[tiab] OR "pérdida de hielo"[tiab] OR "contaminación"[tiab] OR "Naturkatastrophe"[tiab] OR "Erdbeben"[tiab] OR "Überschwemmung"[tiab] OR "Hurrikan"[tiab] OR "Zyklon"[tiab] OR "Waldbrand"[tiab] OR "Dürre"[tiab] OR "Erdrutsch"[tiab] OR "Klimawandel"[tiab] OR "globale Erwärmung"[tiab] OR "Umweltrisiko"[tiab] OR "Umweltverschlechterung"[tiab] OR "Krieg"[tiab] OR "Konflikt"[tiab] OR "erzwungene Migration"[tiab] OR "Flüchtling"[tiab] OR "Binnenvertriebene"[tiab] OR "informelle Siedlung"[tiab] OR "Umweltkrise"[tiab] OR "Vertreibung"[tiab] OR "klimatische Variabilität"[tiab] OR "Klimakrise"[tiab] OR "klimabedingt"[tiab] OR "sich veränderndes Klima"[tiab] OR "Klima-Notfall"[tiab] OR "Klimaschock"[tiab] OR "Wettervariabilität"[tiab] OR "extremes Wetter"[tiab] OR "extreme Hitze"[tiab] OR "extreme Kälte"[tiab] OR "Temperaturanstieg"[tiab] OR "Hitzewelle"[tiab] OR "Sturm"[tiab] OR "Buschfeuer"[tiab] OR "Feuersaison"[tiab] OR "klimabedingte Vertreibung"[tiab] OR "Klimamigration"[tiab] OR "Umweltmigration"[tiab] OR "Ökosystemdegradation"[tiab] OR "ökologische Krise"[tiab] OR "Umweltstress"[tiab] OR "Klimastress"[tiab] OR "Anstieg des Meeresspiegels"[tiab] OR "Küstenerosion"[tiab] OR "Desertifikation"[tiab] OR "Gletscherschmelze"[tiab] OR "Eisverlust"[tiab] OR "Verschmutzung"[tiab] OR "desastre natural"[tiab] OR "terremoto"[tiab] OR "inundação"[tiab] OR "furacão"[tiab] OR "ciclone"[tiab] OR "incêndio florestal"[tiab] OR "seca"[tiab] OR "deslizamento de terra"[tiab] OR "mudança climática"[tiab] OR "aquecimento global"[tiab] OR "risco ambiental"[tiab] OR "degradação ambiental"[tiab] OR "guerra"[tiab] OR "conflito"[tiab] OR "migração forçada"[tiab] OR "refugiado"[tiab] OR "deslocado interno"[tiab] OR "assentamento informal"[tiab] OR "crise ambiental"[tiab] OR "deslocamento"[tiab] OR "variabilidade climática"[tiab] OR "crise climática"[tiab] OR "relacionado ao clima"[tiab] OR "clima em mudança"[tiab] OR "emergência climática"[tiab] OR "choque climático"[tiab] OR "variabilidade do clima"[tiab] OR "tempo extremo"[tiab] OR "calor extremo"[tiab] OR "frio extremo"[tiab] OR "aumento da temperatura"[tiab] OR "onda de calor"[tiab] OR "tempestade"[tiab] OR "incêndio florestal"[tiab] OR "temporada de incêndios"[tiab] OR "deslocamento climático"[tiab] OR "migração climática"[tiab] OR "migração ambiental"[tiab] OR "degradação do ecossistema"[tiab] OR "crise ecológica"[tiab] OR "estresse ambiental"[tiab] OR "estresse climático"[tiab] OR "elevação do nível do mar"[tiab] OR "erosão costeira"[tiab] OR "desertificação"[tiab] OR "degelo glacial"[tiab] OR "perda de gelo"[tiab] OR "poluição"[tiab] OR "culture"[MeSH Terms] OR "traditional medicine practitioners"[MeSH Terms] OR "medicine, traditional"[MeSH Terms] OR "Faith Healing"[MeSH Terms] OR "Awareness"[MeSH Terms] OR "Knowledge"[MeSH Terms] OR "Attitude"[MeSH Terms] OR "Attitude to Health"[MeSH Terms] OR "Perception"[MeSH Terms] OR "Health Literacy"[MeSH Terms] OR "Social Isolation"[MeSH Terms] OR "Ostracism"[MeSH Terms] OR "Social Marginalization"[MeSH Terms] OR "Social Determinants of Health"[MeSH Terms] OR culture*[tiab] OR "cultural practices"[tiab] OR tradition*[tiab] OR ritual*[tiab] OR ceremony*[tiab] OR "social custom*"[tiab] OR heritage[tiab] OR "indigenous practice*"[tiab] OR folk*[tiab] OR folklore*[tiab] OR "cultural norm*"[tiab] OR value*[tiab] OR worldview*[tiab] OR "cultural identit*"[tiab] OR acculturation[tiab] OR enculturation[tiab] OR "cultural adaptation*"[tiab] OR ethnicit*[tiab] OR "social tradition*"[tiab] OR "traditional healer"[tiab] OR faith*[tiab] OR religion*[tiab] OR spirituality[tiab] OR "spiritual belief*"[tiab] OR worship*[tiab] OR theolog*[tiab] OR denomination*[tiab] OR "religious practice*"[tiab] OR "faith-based"[tiab] OR "divine belief*"[tiab] OR "religious identit*"[tiab] OR pilgrimage[tiab] OR sect*[tiab] OR healer*[tiab] OR knowledge*[tiab] OR attitude*[tiab] OR belief*[tiab] OR perception*[tiab] OR awareness[tiab] OR opinion*[tiab] OR practice*[tiab] OR KAP[tiab] OR literacy[tiab] OR "health literacy"[tiab] OR "information uptake"[tiab] OR understanding[tiab] OR "knowledge transfer"[tiab] OR "social exclusion"[tiab] OR "social marginalization"[tiab] OR "social isolation"[tiab] OR "marginalized communit*"[tiab] OR "social disadvantage*"[tiab] OR "excluded communit*"[tiab] OR "structural exclusion"[tiab] OR "systemic exclusion"[tiab] OR "social inequalit*"[tiab] OR discrimination[tiab] OR "social support network*"[tiab] OR "community connection*"[tiab] OR "social capital"[tiab] OR "access to technology"[tiab] OR "access to information"[tiab] OR "digital access"[tiab] OR "technology access"[tiab] OR "culture"[tiab] OR "pratiques culturelles"[tiab] OR "tradition"[tiab] OR "rituel"[tiab] OR "cérémonie"[tiab] OR "coutumes sociales"[tiab] OR "patrimoine"[tiab] OR "pratique autochtone"[tiab] OR "folklore"[tiab] OR "normes culturelles"[tiab] OR "valeurs"[tiab] OR "vision du monde"[tiab] OR "identité culturelle"[tiab] OR "acculturation"[tiab] OR "enculturation"[tiab] OR "adaptation culturelle"[tiab] OR "ethnicité"[tiab] OR "tradition sociale"[tiab] OR "guérisseur traditionnel"[tiab] OR "foi"[tiab] OR "religion"[tiab] OR "spiritualité"[tiab] OR "croyance spirituelle"[tiab] OR "culte"[tiab] OR "théologie"[tiab] OR "dénomination"[tiab] OR "pratique religieuse"[tiab] OR "fondé sur la foi"[tiab] OR "croyance divine"[tiab] OR "identité religieuse"[tiab] OR "pèlerinage"[tiab] OR "secte"[tiab] OR "guérisseur"[tiab] OR "connaissance"[tiab] OR "attitude"[tiab] OR "croyance"[tiab] OR "perception"[tiab] OR "sensibilisation"[tiab] OR "opinion"[tiab] OR "pratique"[tiab] OR "littératie en santé"[tiab] OR "acquisition d'information"[tiab] OR "compréhension"[tiab] OR "transfert de connaissances"[tiab] OR "exclusion sociale"[tiab] OR "marginalisation sociale"[tiab] OR "isolement social"[tiab] OR "communauté marginalisée"[tiab] OR "désavantage social"[tiab] OR "communauté exclue"[tiab] OR "exclusion structurelle"[tiab] OR "exclusion systémique"[tiab] OR "inégalités sociales"[tiab] OR "discrimination"[tiab] OR "réseau de soutien social"[tiab] OR "lien communautaire"[tiab] OR "capital social"[tiab] OR "accès à la technologie"[tiab] OR "accès à l'information"[tiab] OR "accès numérique"[tiab] OR "accès technologique"[tiab] OR "cultura"[tiab] OR "prácticas culturales"[tiab] OR "tradición"[tiab] OR "ritual"[tiab] OR "ceremonia"[tiab] OR "costumbres sociales"[tiab] OR "patrimonio"[tiab] OR "práctica indígena"[tiab] OR "folclore"[tiab] OR "normas culturales"[tiab] OR "valores"[tiab] OR "cosmovisión"[tiab] OR "identidad cultural"[tiab] OR "aculturación"[tiab] OR "enculturación"[tiab] OR "adaptación cultural"[tiab] OR "etnicidad"[tiab] OR "tradición social"[tiab] OR "curandero tradicional"[tiab] OR "fe"[tiab] OR "religión"[tiab] OR "espiritualidad"[tiab] OR "creencia espiritual"[tiab] OR "adoración"[tiab] OR "teología"[tiab] OR "denominación"[tiab] OR "práctica religiosa"[tiab] OR "basado en la fe"[tiab] OR "creencia divina"[tiab] OR "identidad religiosa"[tiab] OR "peregrinación"[tiab] OR "secta"[tiab] OR "curandero"[tiab] OR "conocimiento"[tiab] OR "actitud"[tiab] OR "creencia"[tiab] OR "percepción"[tiab] OR "conciencia"[tiab] OR "opinión"[tiab] OR "práctica"[tiab] OR "alfabetización en salud"[tiab] OR "adquisición de información"[tiab] OR "comprensión"[tiab] OR "transferencia de conocimiento"[tiab] OR "exclusión social"[tiab] OR "marginalización social"[tiab] OR "aislamiento social"[tiab] OR "comunidad marginada"[tiab] OR "desventaja social"[tiab] OR "comunidad excluida"[tiab] OR "exclusión estructural"[tiab] OR "exclusión sistémica"[tiab] OR "desigualdades sociales"[tiab] OR "discriminación"[tiab] OR "red de apoyo social"[tiab] OR "conexión comunitaria"[tiab] OR "capital social"[tiab] OR "acceso a la tecnología"[tiab] OR "acceso a la información"[tiab] OR "acceso digital"[tiab] OR "acceso tecnológico"[tiab] OR "Kultur"[tiab] OR "kulturelle Praktiken"[tiab] OR "Tradition"[tiab] OR "Ritual"[tiab] OR "Zeremonie"[tiab] OR "soziale Bräuche"[tiab] OR "kulturelles Erbe"[tiab] OR "indigene Praxis"[tiab] OR "Volkskultur"[tiab] OR "kulturelle Normen"[tiab] OR "Werte"[tiab] OR "Weltanschauung"[tiab] OR "kulturelle Identität"[tiab] OR "Akkulturation"[tiab] OR "Enkulturation"[tiab] OR "kulturelle Anpassung"[tiab] OR "Ethnizität"[tiab] OR "soziale Tradition"[tiab] OR "traditioneller Heiler"[tiab] OR "Glaube"[tiab] OR "Religion"[tiab] OR "Spiritualität"[tiab] OR "spiritueller Glaube"[tiab] OR "Anbetung"[tiab] OR "Theologie"[tiab] OR "Konfession"[tiab] OR "religiöse Praxis"[tiab] OR "glaubensbasiert"[tiab] OR "göttlicher Glaube"[tiab] OR "religiöse Identität"[tiab] OR "Pilgerreise"[tiab] OR "Sekte"[tiab] OR "Heiler"[tiab] OR "Wissen"[tiab] OR "Einstellung"[tiab] OR "Glauben"[tiab] OR "Wahrnehmung"[tiab] OR "Bewusstsein"[tiab] OR "Meinung"[tiab] OR "Praxis"[tiab] OR "Gesundheitskompetenz"[tiab] OR "Informationsaufnahme"[tiab] OR "Verständnis"[tiab] OR "Wissensweitergabe"[tiab] OR "soziale Ausgrenzung"[tiab] OR "soziale Marginalisierung"[tiab] OR "soziale Isolation"[tiab] OR "marginalisierte Gemeinschaft"[tiab] OR "soziale Benachteiligung"[tiab] OR "ausgeschlossene Gemeinschaft"[tiab] OR "strukturelle Ausgrenzung"[tiab] OR "systemische Ausgrenzung"[tiab] OR "soziale Ungleichheiten"[tiab] OR "Diskriminierung"[tiab] OR "soziales Unterstützungsnetzwerk"[tiab] OR "gemeinschaftliche Verbindung"[tiab] OR "soziales Kapital"[tiab] OR "Zugang zu Technologie"[tiab] OR "Zugang zu Informationen"[tiab] OR "digitaler Zugang"[tiab] OR "technologischer Zugang"[tiab] OR "cultura"[tiab] OR "práticas culturais"[tiab] OR "tradição"[tiab] OR "ritual"[tiab] OR "cerimônia"[tiab] OR "costumes sociais"[tiab] OR "patrimônio"[tiab] OR "prática indígena"[tiab] OR "folclore"[tiab] OR "normas culturais"[tiab] OR "valores"[tiab] OR "visão de mundo"[tiab] OR "identidade cultural"[tiab] OR "aculturação"[tiab] OR "enculturação"[tiab] OR "adaptação cultural"[tiab] OR "etnicidade"[tiab] OR "tradição social"[tiab] OR "curandeiro tradicional"[tiab] OR "fé"[tiab] OR "religião"[tiab] OR "espiritualidade"[tiab] OR "crença espiritual"[tiab] OR "adoração"[tiab] OR "teologia"[tiab] OR "denominação"[tiab] OR "prática religiosa"[tiab] OR "baseado na fé"[tiab] OR "crença divina"[tiab] OR "identidade religiosa"[tiab] OR "peregrinação"[tiab] OR "seita"[tiab] OR "curandeiro"[tiab] OR "conhecimento"[tiab] OR "atitude"[tiab] OR "crença"[tiab] OR "percepção"[tiab] OR "consciência"[tiab] OR "opinião"[tiab] OR "prática"[tiab] OR "alfabetização em saúde"[tiab] OR "obtenção de informação"[tiab] OR "compreensão"[tiab] OR "transferência de conhecimento"[tiab] OR "exclusão social"[tiab] OR "marginalização social"[tiab] OR "isolamento social"[tiab] OR "comunidade marginalizada"[tiab] OR "desvantagem social"[tiab] OR "comunidade excluída"[tiab] OR "exclusão estrutural"[tiab] OR "exclusão sistêmica"[tiab] OR "desigualdades sociais"[tiab] OR "discriminação"[tiab] OR "rede de apoio social"[tiab] OR "conexão comunitária"[tiab] OR "capital social"[tiab] OR "acesso à tecnologia"[tiab] OR "acesso à informação"[tiab] OR "acesso digital"[tiab] OR "acesso tecnológico"[tiab])
