# Supplementary material for: Mental health, social determinants and One Health in skin neglected tropical diseases (NTDs): A scoping review
Source: PLoS Negl Trop Dis. 2026 Aug 6;20(8):e0014034. doi: 10.1371/journal.pntd.0014034 (PMC13485127; doi:10.1371/journal.pntd.0014034)
Supplement: Supplementary file 3 [file pntd.0014034.s003.docx]

**DATA EXTRACTION QUESTIONS**

Using the article **“Record_ID_1”** as your source, answer all questions and statements listed in the document **“DATA EXTRACTION QUESTION LIST_REVISED W SCREENING 3.0.pdf“ .**For each question, provide concise bullet points summarizing the extracted information. Where the study explicitly presents findings, include **verbatim quotes** from the article as supporting evidence, enclosing each quote in quotation marks. If a statement or finding is **not derived directly from the study’s data** (for example, if the authors are citing prior literature), please include the **citation referenced in the article**, formatted in **Harvard referencing style**. In cases where a question cannot be answered because the article does not address the topic, clearly indicate that the **information is not covered** in the study, and note any **other relevant observations** that might inform the question. Overall, be comprehensive (AND MAKE SURE YOU PROVIDE VERBATIM QUOTES FOR ALL EXTRACTION QUESTIONS AS YOU KEEP MISSING IT). Please ensure that you include citations in Harvard referencing style, alongside relevant quotes.

# **SCREENING**

# **Q0 — Screening (Inclusion/Exclusion)**

**How to use:** Answer each item in bullets. Include short verbatim quotes in quotation marks. End with **Q0.SUM**.

### **Q0.1 – Publication type (Inclusion)**

**Question:** Is the article a **peer-reviewed** primary research article or review?
 **Include examples:** “peer-reviewed empirical study”, “systematic review”, “randomized controlled trial”, “cross-sectional survey”, “qualitative ethnography”.
 **Exclude examples:** “conference abstract”, “preprint without peer review”, “dissertation”, “grey literature report/brief”.

### **Q0.2 – Skin NTD focus (Inclusion)**

**Question:** Does the article focus on **at least one skin NTD** with skin manifestations **central**?
 **Examples (incl. synonyms/etiologic agents):** Leprosy (Mycobacterium leprae; multibacillary/paucibacillary/lepromatous/tuberculoid/borderline); Buruli ulcer (Mycobacterium ulcerans); Cutaneous/Mucocutaneous/Diffuse CL, PKDL; Lymphatic filariasis (elephantiasis; Wuchereria bancrofti; Brugia spp.; skin/lymphatic manifestations); Chromoblastomycosis (dematiaceous fungi—Fonsecaea/Cladophialophora/Phialophora/Exophiala/Rhinocladiella); Mycetoma (actinomycetoma/eumycetoma; Madurella; “Madura foot”); Sporotrichosis (Sporothrix schenckii/brasiliensis/globosa); Onchocerciasis (**include only if skin disease is central**, e.g., onchodermatitis/pruritus/depigmentation); Scabies (Sarcoptes scabiei; crusted scabies); Tungiasis (Tunga penetrans); Yaws (Treponema pallidum pertenue; framboesia/goundou/bouba); Noma (cancrum oris; severe facial skin/soft tissue).
 **Tricky cases (apply here):**

- **Onchocerciasis**: Include only when **skin disease** is a central focus (not eye disease alone).
- **Lymphatic filariasis**: Include when **skin/lymphatic** manifestations and their social/psychological impacts are addressed.
- **Non-skin NTDs** (e.g., **Chagas**, intestinal **schistosomiasis**): **Exclude** unless a **central skin manifestation** is the analytic focus.

### **Q0.3 – Three domains explicitly present (Inclusion)**

**Question:** Does the study **explicitly engage all three** domains **in the context of a skin NTD**?

- **Mental Health (MH):** mental health/psychological well-being/quality of life; disorders (depression, anxiety, PTSD, OCD, bipolar, psychosis, schizophrenia); **stigma** (public/perceived/enacted/self); **disability/impairment/functioning**.
- **Social Determinants of Health (SDH):** gender/sex; age; ethnicity/race; SES (income, poverty, employment, education/literacy, insurance); **housing/overcrowding**; neighborhood (urban/rural/peri-urban, disinvestment, safety, green space/public facilities); **migration/displacement/conflict**; **food insecurity/transport costs**; **culture/traditional healers/faith**; **social exclusion/support/capital**; **access to information/technology**; climate/weather framed as **social context** affecting livelihoods/exposure.
- **One Health:** **human–animal–environment** interfaces; **zoonoses/reservoirs/intermediate hosts** (bats, rodents, dogs, livestock; **peridomestic/sylvatic**); **agriculture/gardening/subsistence farming**; **environmental exposures/ecosystem change** (e.g., deforestation, floods, droughts, heat waves) **linked back to human health**.
   **Tricky cases (apply here):**
- Studies on CL/PKDL often have clear One Health pathways (reservoirs/vectors) and SDH (housing, livelihoods, displacement); **ensure MH is actually measured/discussed**.
- LF (elephantiasis): look for **stigma/depression/QoL (MH)** + **poverty/employment/gendered caregiving (SDH)** + **vector/ecological or peridomestic links (One Health)**.

### **Q0.4 – Conceptual gaps or wrong NTD (Exclusion)**

**Question:** Does the article **omit** one or more domains (only 1–2 present) **or** is it a **non-skin NTD** without central skin disease?
 **Exclude examples:**

- MH + NTD only (no SDH/One Health), e.g., “depression among leprosy patients” with **no social context or human–animal–environment links**.
- SDH + NTD only (no MH), e.g., “poverty and access to care in CL” without MH constructs.
- One Health + NTD only (no MH), e.g., “rodent reservoirs of Leishmania” without human MH outcomes.
- **Chagas/schistosomiasis** without central skin disease.

### **Q0.5 – Depth and substance (Include vs Exclude)**

**Question:** Where all three domains are “present,” are they **substantively analyzed**, not just name-checked?
 **Include if:** MH outcomes (e.g., depression scores, stigma scales, QoL) **measured/discussed**; SDH indicators **analyzed** (income, gender, education, housing, displacement); One Health components **traced to human health pathways** (reservoirs/vectors/environment → exposure → MH/SDH impacts).
 **Exclude if:** Any domain is **only mentioned in passing** without data/analysis (e.g., “mental health was also affected”).
 **Tricky emphasis:** For **onchocerciasis**, ensure skin disease and its **psychosocial** and **SDH** impacts are analyzed alongside ecological/host/vector pathways.

### **Q0.6 – Disease ecology only (Exclusion unless integrated)**

**Question:** Is the article **primarily disease ecology** (reservoirs, transmission routes, environmental persistence, land-use/climate models) **without integrating** MH **and** SDH in people?
 **Exclude if:** Vector/animal/environment modeling **without** human MH and SDH outcomes.
 **Include if:** Ecology is **explicitly tied back** to human MH outcomes **and** SDH (e.g., “deforestation → vector proliferation → ↑ CL incidence → stigma/anxiety → employment loss”).

### **Q0.7 – Full-text availability (Exclusion)**

**Question:** Is **full text** available?
 **Include:** Full text accessible in any language (note “translation needed” if applicable).
 **Exclude:** Abstract only / full text unavailable.

### **Q0.9 – Quick domain triggers (Helper checklist)**

Use to justify Q0.3 and Q0.5 decisions.

- **MH present if any of:** depression, anxiety, PTSD, OCD, bipolar, psychosis, schizophrenia, “mental health”, QoL, stigma (public/perceived/enacted/self), disability/impairment/functioning, shame/prejudice/discrimination.
- **SDH present if any of:** gender/sex, age, ethnicity/race, SES (income/poverty/employment/education/literacy/insurance), housing/overcrowding, neighborhood (urban/rural/peri-urban; disinvestment; safety; public facilities/green space), migration/displacement/refugee/IDP, conflict/war, **food insecurity/transport costs**, **culture/traditional healers/faith**, social support/exclusion/capital, **access to information/technology**, climate/weather as social context.
- **One Health present if any of:** human–animal–environment interactions, zoonoses/reservoirs/intermediate hosts (bats/rodents/dogs/livestock), peridomestic/sylvatic, agriculture/gardening/subsistence farming, pet ownership/companion animals, environmental exposures/ecosystem change **linked to human outcomes**.

### **Q0.SUM – Inclusion decision**

- **Decision:** Include / Exclude / Borderline–team review
- **Primary reason(s):** (tie to Q0.1–Q0.7)
- **Verbatim support:** Short quotes for key decision points in quotation marks.
- **If Borderline:** Specify which domain(s) are weak/unclear (e.g., “One Health only mentioned; no human MH outcomes measured”).

**_______________________________________________________________________________**

**DATA EXTRACTION**

**1. Lead Author**
Question: What is the lead author’s name (use *et al.* format if multiple)? Answer the question and, in addition, extract *all relevant verbatim quotes* and put quotes in quotation marks.
Example: Diallo I. et al.

**2. Title**
Question: What is the title of the study? Answer the question and, in addition, extract *all relevant verbatim quotes* and put quotes in quotation marks.
Example: *“Mental health impacts of climate change in Mali: A mixed-methods study”*

**3. Year**
Question: In what year was the study published? Answer the question and, in addition, extract *all relevant verbatim quotes* and put quotes in quotation marks.
Example: *“2021”*

**4. Journal/Source**
Question: What is the journal or source of publication? Answer the question and, in addition, extract *all relevant verbatim quotes* and put quotes in quotation marks.
Example: *“BMC Public Health”*; *“WHO Policy Brief on Neglected Tropical Diseases”*

**5. DOI/URL**
Question: What is the DOI or URL of the study? Answer the question and, in addition, extract *all relevant verbatim quotes* and put quotes in quotation marks.
Example: *“10.1186/s12889-021-10025-3”*

**6. Country(ies)**
Question: Which country or countries were studied in this study (focus on the setting of the study not countries loosely described or referred to)? Answer the question and, in addition, extract *all relevant verbatim quotes* and put quotes in quotation marks. If a review, discuss countries where the review occurred.
Example: *“Mali”*; *“Nigeria”*; *“A multi-country study across West Africa including Ghana, Nigeria, and Mali”*

**6B. Geographical Regions (LMICs, African regions, etc.)**

**Question:** Which geographical regions are represented in this study (e.g., Sub-Saharan Africa, West Africa, East Africa, LMICs)? Focus on the broader regional classification of the study setting, not countries merely mentioned in passing. Answer the question and, in addition, extract all relevant verbatim quotes and put quotes in quotation marks.
**Examples:**

- “Sub-Saharan Africa”
- “West African countries including Mali and Ghana”
- “Low- and middle-income countries (LMICs)”
- “Horn of Africa region”

**7. Study Type**
Question: What is the study type (e.g., empirical, review, policy)? Answer the question and, in addition, extract *all relevant verbatim quotes* and put quotes in quotation marks.
Examples: *“Empirical study using mixed-methods”*; *“Systematic review of observational studies”*; *“Qualitative ethnography”*

**8. Study Design**
Question: What is the study design? Answer the question and, in addition, extract *all relevant verbatim quotes* and put quotes in quotation marks.
Examples: *“Cross-sectional household survey”*; *“Longitudinal cohort study following participants over 5 years”*; *“Randomized controlled trial”*

**9. Intervention or Policy Overview**
Question: What intervention or policy is being studied or described (if any)? Answer the question and, in addition, extract *all relevant verbatim quotes* and put quotes in quotation marks.
Examples: *“A community health education campaign to reduce leprosy stigma”*; *“The national Buruli ulcer elimination strategy”*; *“mHealth program for self-monitoring of mental health symptoms”, "this was a an intervention study" "the study did not evaluate an intervention or policy".*

**10. Co-design/Participation**
Question: Was co-design or participation involved in the study? Answer the question and, in addition, extract *all relevant verbatim quotes* and put quotes in quotation marks.
Examples: *“No participation from community members”*; *“Stakeholder workshops were conducted with affected persons”*; *“Study used a community-led participatory design model”*

**11. Study Aims/Objectives**
Question: What are the aims and objectives of the study? Answer the question and, in addition, extract *all relevant verbatim quotes* and put quotes in quotation marks.
Example: *“To explore stigma and mental health among persons affected by leprosy in rural Mali”*

**12. Sample Size**
Question: What is the sample size (answer even for reviews)? Answer the question and, in addition, extract *all relevant verbatim quotes* and put quotes in quotation marks.
Examples: *“N = 350 participants”*; *“A total of 27 in-depth interviews were conducted”*

**13. Setting**
Question: What is the study setting? Answer the question and, in addition, extract *all relevant verbatim quotes* and put quotes in quotation marks.
Examples: *“Hospital outpatient clinic”*; *“Rural community health center”*; *“Urban refugee camp”*

**14. Population Groups**
Question: What population groups are included? Answer the question and, in addition, extract *all relevant verbatim quotes* and put quotes in quotation marks.
Examples: *“Women of reproductive age”*; *“Adolescents affected by Buruli ulcer”*; *“Community health workers”*

**15. Age Group Categories**
Question: Is an age group or the age of the study presented through a breakdown (yes or no in a full sentence)? If yes, what age group categories are represented in the study by the study sample (if discussed)? Answer the question and, in addition, extract *all relevant verbatim quotes* and put them in quotation marks.
Examples: *“Children under 18 years”*; *“Adults aged 18–49”*; *“Older adults aged 50 years and above”, "Age groups or age of study participants not specified in this study"*

**16. Sex/Gender**
Question: Is a sex/gender breakdown presented for the study sample (yes or no in a full sentence)? If yes, what sex or gender groups are represented? Answer the questions and, in addition, extract *all relevant verbatim quotes* and put quotes in quotation marks.
Examples: *“Male participants only”*; *“Mixed sample of men and women”*; *“Gender not reported”*

**17. NTD Condition**
Question: Which neglected tropical disease condition(s) are addressed? Answer the question and, in addition, extract *all relevant verbatim quotes* and put quotes in quotation marks.
Examples: *“Leprosy”*; *“Buruli ulcer”*; *“Lymphatic filariasis”*

**18. MH Constructs**
Question: Which mental health constructs are examined? Answer the question and, in addition, extract *all relevant verbatim quotes* and put quotes in quotation marks.
Examples: *“Depression”*; *“Anxiety”*; *“Stigma and discrimination”*; *“Quality of life”*

**19. MH Instruments**
Question: Are mental health measures used (yes or no in a full sentence)? What mental health instruments or measures were used (if any)? Answer the questions and, in addition, extract *all relevant verbatim quotes* and put quotes in quotation marks.
Examples: *“CES-D”*; *“GAD-7”*; *“WHOQOL-BREF”*

**20. MH Outcome Focus/Discussion**
Question: What are the findings or main discussions related to mental health outcomes? Answer the question and, in addition, extract *all relevant verbatim quotes* and put quotes in quotation marks.
Examples: *“High levels of depressive symptoms were reported”*; *“Suicidal ideation was associated with stigma”*; *“Improved functional outcomes following intervention”*

**21. SDH Domains**
Question: Which social determinants of health domains or indicators are included? Answer the question and, in addition, extract *all relevant verbatim quotes* and put quotes in quotation marks.
Examples: *“Education”*; *“Employment”*; *“Housing conditions”*; *“Food insecurity”*

**22. SDH Indicators/Discussion**
Question: What are the findings or main discussions around specific SDH indicators? Answer the question and, in addition, extract *all relevant verbatim quotes* and put quotes in quotation marks.
Examples: *“Illiteracy rates were higher among persons affected by leprosy”*; *“Household income was below the poverty line”*; *“Food insecurity was a common stressor”*

**23. One Health Components**
Question: Which One Health components are included (if any)? Answer the question and, in addition, extract *all relevant verbatim quotes* and put quotes in quotation marks.
Examples: *“Human health”*; *“Animal health”*; *“Environmental health”*

**24. One Health Component(s) Discussion**
Question: What are the findings or main discussions of One Health components? Answer the question and, in addition, extract *all relevant verbatim quotes* and put quotes in quotation marks.
Examples: *“Deforestation increased exposure to disease vectors”*; *“Livestock transmission pathways worsened stigma”*

**25. Cross-Sectoral Collaborations/Actors**
Question: Are cross-sectoral collaborators mentioned (yes or no in a full sentence)? If yes, what cross-sectoral collaborations or actors are mentioned (within the study and not by authorship of the paper, if at all discussed)? Answer the question and, in addition, extract *all relevant verbatim quotes* and put quotes in quotation marks.
Examples: *“Partnership with Ministry of Health”*; *“Collaboration with NGOs and traditional healers”*

**26. Conceptual Frameworks**
Question: Are conceptual frameworks used to contextualize the study or the study findings (Yes or no in a full sentence)? And if yes, please say so and indicate which ones? Answer the questions and, in addition, extract *all relevant verbatim quotes* and put quotes in quotation marks.
Examples: *“Social-ecological model”*; *“Health belief model”*; *“One Health framework”*

**26B. Mechanistic Pathway Linked to One Health**

**Question:** Is a mechanistic pathway explicitly linked to the One Health framework discussed in the paper (yes or no in a full sentence)? If yes, describe how human, animal, and/or environmental components are integrated or interact within that pathway. Answer the question and, in addition, extract all relevant verbatim quotes and put quotes in quotation marks.
**Examples:**

- “The study explicitly connects environmental degradation to increased vector exposure, leading to mental distress.”
- “A One Health pathway linking livestock management practices, zoonotic infections, and psychosocial outcomes was described.”
- “No explicit mechanistic pathway linked to One Health was discussed.”

**27. Proposed Mechanisms/Pathways**
Question: What mechanisms or pathways are proposed on the link between mental health, one health, social determinants of health, and one health? Answer the question and, in addition, extract *all relevant verbatim quotes* and put quotes in quotation marks.
Example: *“Climate stress → food insecurity → depression and anxiety”*

**27B. Diagram of Proposed Pathway**

**Question:** Does the paper include a visual or conceptual diagram illustrating the proposed mechanisms or pathways (yes or no in a full sentence)? If yes, describe or summarize the key elements shown in the figure or diagram and indicate figure number. Answer the question and, in addition, extract all relevant verbatim quotes and put them in quotation marks.
**Examples:**

- “Figure 2 illustrates the conceptual model linking climate stress, food insecurity, and mental health outcomes.”
- “The study includes a causal pathway diagram demonstrating environmental, biological, and psychosocial interactions.”
- “No diagram of the proposed pathway was included.”

**28. Intersection of SDH, Mental Health, and One Health in NTDs**
Question: How do social determinants of health, mental health, and One Health components intersect in the context of skin NTDs (or other relevant NTDs)? Answer the question and, in addition, extract *all relevant verbatim quotes* and put quotes in quotation marks.
Examples: *“Stigma related to leprosy limited employment opportunities, which worsened poverty and exacerbated depression.”*; *“Environmental degradation and poor sanitation increased exposure to Buruli ulcer, with psychological distress linked to disability and social exclusion.”*

**29. Primary Outcomes**
Question: What are the primary outcomes? Answer the question and, in addition, extract *all relevant verbatim quotes* and put quotes in quotation marks.
Examples: *“Depression scores were reduced following intervention”*; *“Treatment adherence improved among participants”*

**30. Secondary Outcomes**
Question: What are the secondary outcomes (if any)? Answer the question and, in addition, extract *all relevant verbatim quotes* and put quotes in quotation marks.
Examples: *“Knowledge of NTD prevention increased”*; *“Community stigma decreased”*; *“Greater school attendance among affected children”, "No secondary outcomes"*

**31. Key Findings**
Question: What are the key findings? Answer the question and, in addition, extract *all relevant verbatim quotes* and put quotes in quotation marks.
Example: *“Depression prevalence was 35% among persons affected by leprosy in rural areas”*

**32. Gendered Dimensions**
Question: What cross-cutting gendered dimensions are discussed or implied in the study (if any)? Consider how gender influences study design, participation, population groups, health outcomes, social determinants, One Health factors, and recommendations. Answer the question and, in addition, extract *all relevant verbatim quotes* and put quotes in quotation marks.

E**xamples**: “Women were disproportionately excluded from community decision-making processes” “Men reported higher levels of stigma related to visible disability compared to women” “Gender roles shaped access to treatment, with women more likely to delay care due to caregiving responsibilities” “Findings highlighted intersectional vulnerabilities, particularly among young women in rural areas” “Gender not reported”

**33. Challenges/Limitations**
Question: What challenges or limitations of the study are reported? (e.g., issues with bias or sample size) Answer the question and, in addition, extract *all relevant verbatim quotes* and put quotes in quotation marks.
Examples: *“Small sample size limited statistical power”*; *“Self-report bias may have influenced responses”*; *“Generalizability is limited to rural settings”*

**34. Recommendations**
Question: What recommendations are made (if any, especially as they relate to mental health, skin NTDS, social determinants of health, and one health? Answer the question and, in addition, extract *all relevant verbatim quotes* and put quotes in quotation marks.
Examples: *“Scale up integration of mental health services into NTD programs”*; *“Strengthen community-based psychosocial support”*

**35. Explicit One Health Mention**
**Question:** Does the paper explicitly mention “One Health”? Answer the question and, in addition, extract all relevant verbatim quotes and put quotes in quotation marks.
**Examples:** “The study was framed using a One Health approach”; “One Health was not explicitly mentioned in the article.”
