# Supplementary material for: Mental health, social determinants and One Health in skin neglected tropical diseases (NTDs): A scoping review
Source: PLoS Negl Trop Dis. 2026 Aug 6;20(8):e0014034. doi: 10.1371/journal.pntd.0014034 (PMC13485127; doi:10.1371/journal.pntd.0014034)
Supplement: Supplementary file 4 [file pntd.0014034.s004.docx]

**Extraction process**

1. Select a paper assigned to you in the [hyperlink to excel sheet] – filter so you can just see this paper, for ease
2. Read the paper in full – ask yourself if its eligible for inclusion (see criteria below). If not, please mark on the sheet and give reason (we can review those marked for exclusion later on and come to agreement as a team)

| **Inclusion criteria** | **Exclusion criteria** |
| --- | --- |
| Peer-reviewed primary research articles or reviews | Articles that focus on non-skin NTDs (e.g., Chagas, schistosomiasis, onchocerciasis unless a skin manifestation is central) |
| Articles that explicitly engage with all three conceptual domains: mental health, social determinants of health, and One Health, within the context of at least one skin NTD | Articles that mention only one or two of the conceptual domains (e.g., mental health and skin NTDs without SDH or One Health framing) |
| Articles published in all languages | Grey literature, conference abstracts, dissertations, and non-peer-reviewed materials |
| Articles published from database inception | Articles not available in full text |
|  | Articles where all three conceptual domains are discussed, but only in passing e.g., if mental health is only mentioned once or twice and isn’t a focus |
|  | Article focuses on disease ecology of the respective skin NTD e.g., Reservoir system, Transmission routes, Persistence in environment, Effects of climate, land use change on the reservoir system |

3. Open [notebooklm](https://notebooklm.google.com/notebook/d4276630-1a1f-48fb-b6ec-90b01643b9ff) and open the batch you’re working on
4. Tick the question sheet and the article you’re reviewing as **the only two sources**
5. Enter the prompt:

*Answer the questions and statements in the document "DATA EXTRACTION QUESTION LIST" using the document "Record_ID_1" as a source using bullet points. Where findings or statements are not derived from the study directly ie they are citing literature, please include the citation they used in Harvard referencing style.* Work through the list of answers, cross checking the extract (hovering over the numbers) and pasting in **the answers + the associated verbatim quotes** into the extraction form

6. Obviously, AI isn’t perfect, so try and think critically about whether the summaries are accurate and whether the system is missing any important details – suggest going in and out of the pdf to check this as you go and checking that the quotations align with the summaries.
7. If you feel that there’s additional info / quotes that should be included, please add to the excel sheet.
8. When you’ve finished, please enter the date of extraction on the sheet and add any necessary comments.
9. Throughout the process, you might notice that there are questions missing etc – if you think of anything, please email the group and we can agree on adaptions to the question form/process.
