# Supplementary material for: Mental health, social determinants and One Health in skin neglected tropical diseases (NTDs): A scoping review
Source: PLoS Negl Trop Dis. 2026 Aug 6;20(8):e0014034. doi: 10.1371/journal.pntd.0014034 (PMC13485127; doi:10.1371/journal.pntd.0014034)
Supplement: Supplementary file 5 [file pntd.0014034.s006.docx]

**Additional Editor Comments (if provided):**

 All three reviewers find this to be an important and well-conducted scoping review. There is no conflict between their comments. Please address their various comments, particularly Reviewer 3 who has given detailed comments.

**Journal Requirements:**

If the reviewer comments include a recommendation to cite specific previously published works, please review and evaluate these publications to determine whether they are relevant and should be cited. There is no requirement to cite these works unless the editor has indicated otherwise.

1) Please upload all main figures as separate Figure files in .tif or .eps format. For more information about how to convert and format your figure files please see our guidelines: 
[https://journals.plos.org/plosntds/s/figures](https://url.uk.m.mimecastprotect.com/s/GwZECAMzliV9Dr7CYsOcGO05I?domain=journals.plos.org)

2) Please upload a copy of Figure Fig 1 and 2 which you refer to in your text on pages 11, and 13. Or, if the figure is no longer to be included as part of the submission please remove all reference to it within the text.

Thank you, we have now addressed this and the point above.

3) As required by our policy on Data Availability, please ensure your manuscript or supplementary information includes the following:
- A numbered table of all studies identified in the literature search, including those that were excluded from the analyses.
- For every excluded study, the table should list the reason(s) for exclusion.
- If any of the included studies are unpublished, include a link (URL) to the primary source or detailed information about how the content can be accessed.
- A table of all data extracted from the primary research sources for the systematic review and/or meta-analysis. The table must include the following information for each study:
  - Name of data extractors and date of data extraction
  - Confirmation that the study was eligible to be included in the review.
  - All data extracted from each study for the reported systematic review and/or meta-analysis that would be needed to replicate your analyses.

Thank you. All of the above information is now available in SI3 Screening and extraction table, which we have cited in the manuscript (line: 206)

  - If data or supporting information were obtained from another source (e.g. correspondence with the author of the original research article), please provide the source of data and dates on which the data/information were obtained by your research group.

All data was obtained from the manuscripts directly, so this is not applicable.

  - If applicable for your analysis, a table showing the completed risk of bias and quality/certainty assessments for each study or outcome.  Please ensure this is provided for each domain or parameter assessed. For example, if you used the Cochrane risk-of-bias tool for randomized trials, provide answers to each of the signalling questions for each study. If you used GRADE to assess certainty of evidence, provide judgements about each of the quality of evidence factor. This should be provided for each outcome.

As this was a scoping review, it did not include any risk of bias and quality/certainty assessments, and this point is not applicable.

  - An explanation of how missing data were handled.
This information can be included in the main text, supplementary information, or relevant data repository. Please note that providing these underlying data is a requirement for publication in this journal, and if these data are not provided your manuscript might be rejected.

Thank you. As this is a scoping review and did not entail statistical analysis or meta-analysis, this is not applicable. We have highlighted this, as well as explaining that where study reporting was incomplete, unavailable information was documented as ‘not reported’ and no imputations were performed (line: 227-230)

4) We have amended your Competing Interest statement to comply with journal style. We kindly ask that you double check the statement and let us know if anything is incorrect.

Thank you, we confirm that the statement is accurate.

**Reviewers' comments:**

Reviewer's Responses to Questions

**Key Review Criteria Required for Acceptance?**

As you describe the new analyses required for acceptance, please consider the following:

**Methods**
-Are the objectives of the study clearly articulated with a clear testable hypothesis stated?

-Is the study design appropriate to address the stated objectives?

-Is the population clearly described and appropriate for the hypothesis being tested?

-Is the sample size sufficient to ensure adequate power to address the hypothesis being tested?

-Were correct statistical analysis used to support conclusions?

-Are there concerns about ethical or regulatory requirements being met?

Reviewer #1: The objectives of the study are clearly articulated and appropriate for a scoping review, aiming to map evidence and identify gaps at the intersection of mental health, social determinants, and One Health in skin NTDs. While no explicit testable hypothesis is stated, this is acceptable given the exploratory nature of the study. The scoping review design is suitable and aligns with the stated aims; however, the requirement that studies address all four domains may have been overly restrictive and limited the number of included studies (n=22).

The population is clearly described and appropriate. As this is a scoping review, issues of sample size and statistical power are not directly applicable, though the limited number of included studies somewhat constrains generalizability. The use of descriptive thematic analysis is appropriate, but the synthesis remains largely descriptive and could benefit from deeper analytical integration.

There are no major ethical concerns, as the study is based on published literature. However, the use of AI tools for screening and data extraction requires clearer reporting to ensure transparency and reproducibility. Overall, the methods are appropriate, with minor clarifications needed.

Thank you for this feedback. We agree with these reflections and have now highlighted the small sample size limiting generalizability and suggesting that we were perhaps overly restrictive, within the limitations section (line: 744-751). Whilst we recognise that if we had used broader criteria, we may have found more papers that were theoretically aligned. However, we want to emphasise that our application of the ‘one health’ component of the search strategy was quite broad, in that the paper only had to include a search term related to either animals or the environment, and the use of the specific term ‘One Health’ was not required. We have now emphasised this within the eligibility criteria (line: 182-183).

We are unaware of a universal framework for reporting on the use of AI in study methodologies. However, after reviewing a proposed [Transparent Reporting of AI in Systematic Literature Reviews: Development of the PRISMA-trAIce Checklist](https://ai.jmir.org/2025/1/e80247) and PLOS NTD’s AI policy, we feel that the level of detail that we have provided is sufficient. In addition to this, we have added a citation for Chat GPT along with the extraction guide (SI4), which provides more details on the step-by-step approach, as well as specifying the input data for NotebookLM (line: 217) We have also now included the extraction table for further transparency (SI5).

Reviewer #2: The study objectives are clearly stated and the use of a scoping review design is appropriate for exploring the emerging intersection of mental health, social determinants, One Health (OH), and skin NTDs. The search strategy and eligibility criteria are generally well described and transparently reported. However, the review includes a relatively small and highly heterogeneous body of literature, with most studies focusing on single diseases and few explicitly applying integrated OH frameworks. In addition, many included studies did not directly measure mental health outcomes using validated tools, limiting the strength of some conceptual conclusions. While the descriptive thematic synthesis is suitable for a scoping review, the absence of formal quality appraisal or risk-of-bias assessment makes it difficult to judge the robustness of the evidence.

Many thanks for this reflection. We feel that the shortcomings you mentioned are highlighted within our results and discussion sections, though we have added a reflection to the limitations section on the implication of the small sample size for generalizability, as well as the lack of studies using validated tools to measure mental health outcomes (line: 744-753).

The manuscript is commendably transparent regarding the use of AI-assisted tools for screening and data extraction, although concerns related to reproducibility, extraction bias, and consistency of interpretation remain and should be more explicitly acknowledged when interpreting the findings.

Thank you for this feedback. We agree with these reflections and have now highlighted the small sample size limiting generalizability and acknowledge the heterogenous and small number of studies identified, within the limitations section (line: 744-753). After reviewing a proposed [Transparent Reporting of AI in Systematic Literature Reviews: Development of the PRISMA-trAIce Checklist](https://ai.jmir.org/2025/1/e80247) and PLOS NTD’s AI policy, we feel that the level of detail that we have provided is sufficient. However, we also added a citation for Chat GPT along with the extraction guide, which provide more details on the step-by-step approach, as well as specifying the input data for NotebookLM (SI3). We have also now included the extraction table for further transparency (SI5)

We have also expanded our limitations section to include the challenges you have mentioned, explicitly raising the risks of extraction bias and consistency of interpretation (line: 740).

Reviewer #3: Yes overall. This concerns a scoping review, so sample size and statistical analysis are not relevant.
No concerns regarding methodology and ethics.

Many thanks for this positive feedback.

**Results**


-Does the analysis presented match the analysis plan?

-Are the results clearly and completely presented?

-Are the figures (Tables, Images) of sufficient quality for clarity?

Reviewer #1: The analysis presented is generally consistent with the stated analysis plan. The authors indicate a descriptive thematic analysis approach, and the results reflect this, with findings structured across key domains such as mental health, One Health dimensions, and social determinants. However, the analysis remains largely descriptive, and a clearer linkage between the extraction framework and the presented themes would improve transparency.

The results are clearly presented and well-organized, with logical subheadings and a coherent narrative. Key findings, such as the limited number of studies (n=22), the dominance of quantitative designs, and the lack of integrated One Health approaches, are communicated effectively. That said, some sections are repetitive, and the presentation could benefit from more concise synthesis and clearer highlighting of the most important insights.

The figures and tables are generally of good quality and support understanding. The PRISMA flow diagram (Figure 1) is clear and appropriate, and the conceptual framework (Figure 2) adds value by illustrating the relationships between mental health, social determinants, and One Health dimensions. Minor improvements in figure labeling and integration into the text could further enhance clarity.

Many thanks for your feedback. We recognise that some of the contents is quite descriptive and there is some overlap. This was a major focus during the write up, but unfortunately due to the scoping review methodology and overlapping domains of mental health, one health and social determinants of health, we feel that no further synthesis can be made without losing clarity. We have now added the risk factors for mental health in each study to the study characteristics table to highlight the important insights (table 1).

Many thanks for the feedback regarding the figure – we have now revised it to make it clearer (line: 318)

Reviewer #2: The findings are presented clearly overall, and the authors appropriately acknowledge the limited and heterogeneous nature of the evidence base. However, some sections move beyond descriptive synthesis into broader conceptual interpretation, particularly regarding OH integration, despite the relatively small number of studies explicitly addressing OH approaches. The presentation would also benefit from clearer differentiation between directly measured findings and interpretive inferences made by the authors. The PRISMA flow diagram and conceptual framework figure are useful and generally of adequate quality, although Figure 2 is conceptually dense and may benefit from simplification or clearer labeling to improve readability. In addition, summary tables synthesizing key study characteristics, mental health outcomes, and OH dimensions in a more concise comparative format would strengthen clarity and accessibility of the results section.

Many thanks for the feedback regarding the figure – we have now revised it to make it clearer (line: 318)

Thank you for sharing your valid concerns regarding the interpretive inferences made by us versus what was stated by the authors themselves. Generally, the degree of our own inferences beyond those of the authors are limited in the results, but we recognise that this could be clearer. We have added the following sentence to the start of the one health section “Therefore, our interpretation presented below draws on OH synthesis to consider how reported changes in SDH may plausibly relate to mental health outcomes, even when this linkage is not made explicit by study authors” (line: 378-380). We have also re-read the results section and made the distinction between ours and authors inferences clearer throughout.

Additionally, we have updated the study characteristics table to include the risk factors explored in each study and the outcomes measured, as well as embedding it within the manuscript body (table 1).

Reviewer #3: Line 246: Since only a relatively small number of studies were included, it would have been helpful to have a table indicating some key parameters, such as where the study was done, the sample size, risk factors assessed and outcome measures (where relevant).

Many thanks for this feedback. Please note that the supplementary table of study characteristics has been changed to an embedded table (table 1) for the reader’s ease. We have also incorporated the risk factors and outcome measures, as suggested.

Line 247: Figure 1 has asterisks (**) against ‘Records excluded’, presumably pointing to explanations of why these were excluded. However, a table footer providing these explanations is missing. Kindly add this. Because of the large number of articles excluded and the role of AI in this, reasons for exclusion are important.
Also, the Included studies box includes “Reports of included studies (n=0”. It is not clear what this means or why it is there if n=0.

Many thanks for this feedback, we have now updated the PRISMA chart to omit the content you highlighted, which was included in the original PRISMA template and is irrelevant to our study (line: 243)

Line 269: Suggest rephrasing, since ‘Most’ is not relevant here: “Thirteen studies used quantitative and 6 used qualitative methods.”

Many thanks, this has now been amended (line: 278)

Line 286: “none of the frameworks applied a OH approach to mental illness in persons affected by skin NTDs”. It is not clear what this means, since mental illness in animals is presumably not relevant? So does it concern anxiety about animal health caused by NTDs, contributing to mental ill-health? Despite the elaboration in the subsequent paragraphs, it doesn’t become clear how the authors envisage the relationship or interaction between animal health and human mental health.

Thank you for raising this. We have now amended it to “none of the articles explicitly applied a OH framework in considering the impact of NTDs on human mental health, for example through planetary/environmental factors or the relevance of diseases having zoonotic pathway” (line: 295-297).

Line 427: I wonder if this is the right reference, since the article is about leishmaniasis, rather than leprosy?

Many thanks for spotting this error, which has now been amended, with the reference updated.

Line 466: Is 58 the wrong reference, or should the country by Tunisia, instead of Tanzania?

Again, thanks so much for spotting this error. It should have been Tunisia, and has now been corrected (line: 484)

Line 492-4: “Only two studies considered the role of existing health status. An Indonesian study found that milder LF symptoms reduced healthcare-seeking behaviour.” This doesn’t seem to be demographics? This whole section doesn’t mention One Health at all. The reported results are likely to be true, but it is not possible to judge the extent to which they are biased by excluding so many studies that did not include the OH concept.
The same could be said for the section on economic factors. Many studies are excluded because of the above bias. Most studies that measured social and work participation were excluded for that reason.

Thank you for highlighting this important point, which we agree with. We have now added a reflection on this source of bias within the strengths and limitations section of the discussion (line: 744-753). Lund’s social determinants of health model includes longevity and survival under demographics, so we decided that health status fit best within this section. However, in response to your comment, we have amended the sub-heading to ‘demographic and health status’ (line: 509)

Line 578: Since the results have been quite non-specific and made hardly any reference to OH, I would recommend calling these at best recommendations for ‘good practice’, since ‘best’ suggests they have been tested against or at least compared with others and found to be better.

Many thanks for this feedback, we have amended the wording as you suggest (line: 601)

**Conclusions**


-Are the conclusions supported by the data presented?

-Are the limitations of analysis clearly described?

-Do the authors discuss how these data can be helpful to advance our understanding of the topic under study?

-Is public health relevance addressed?

Reviewer #1: The conclusions are supported by the data presented, appropriately highlighting the limited evidence base and lack of integrated One Health approaches without overinterpretation.

The limitations are clearly described, including the small number of included studies, heterogeneity, and methodological constraints such as the use of AI tools and exclusion of grey literature.

The authors adequately discuss how the findings advance understanding by identifying key gaps and priorities for future research, particularly around integration of mental health and One Health. Public health relevance is clearly addressed, with strong links to global health priorities and implications for policy and health systems.

Many thanks for this positive feedback.

Reviewer #2: The conclusions are generally consistent with the overall findings of the review and appropriately emphasize the limited and fragmented evidence base linking mental health, social determinants, and One Health approaches in skin NTDs. However, some conclusions regarding integrated OH mechanisms and frameworks appear stronger than the available evidence can fully support, given that only a small number of studies explicitly operationalized OH approaches. The limitations of the review are transparently acknowledged, including the heterogeneity of included studies, limited intervention evidence, exclusion of grey literature, and challenges related to AI-assisted screening and extraction. The discussion appropriately highlights important research and policy implications, particularly the need for participatory, interdisciplinary, and person centred approaches. However, the manuscript could further strengthen the conclusions by more cautiously distinguishing between evidence directly supported by the included studies and broader conceptual or theoretical interpretations proposed by the authors.

Thank you for this helpful feedback. In response, we have added a reflection on the limited generalizability due to the sample size within the limitations section (line: 744-753). We have also added the following sentence to the conclusion: “Ultimately, whilst this review was able to contribute to the evidence base for understanding NTDs framed around OH, more empirical studies directly measuring mental health outcomes in relation to OH determinants are required to support and validate the broader conceptual interpretations proposed” (line: 766-769).

Reviewer #3: Yes, with the limitation that OneHealth was little mentioned, even though the studies met the inclusion criteria.

Line 674-6: “OH plays a key role in shaping experiences of mental health in persons affected by skin NTDs, largely through SDH, such as changing environment leading to increased risk to traditional livelihoods.” It should be noted that this is still a hypothesis, since the current review did not find clear evidence to support this.

Thank you, we have now added this caveat (line: 699)

Limitations:
Line 694: the above-mentioned bias in the article selection because of using OH as a co-selection criterion is not mentioned. Many studies on Skin NTDs, stigma, mental health, social participation and even economic aspects were not included because of this.

Thank you. We have now addressed this comment, as detailed in earlier response (line: 744-751)

**Editorial and Data Presentation Modifications?**


Use this section for editorial suggestions as well as relatively minor modifications of existing data that would enhance clarity. If the only modifications needed are minor and/or editorial, you may wish to recommend “Minor Revision” or “Accept”.

Reviewer #1: The manuscript is generally well written and clearly structured, but a few editorial and presentation improvements would enhance clarity and readability. Some sections, particularly the Results and Discussion, are repetitive and could be streamlined to reduce redundancy (e.g., repeated emphasis on "limited evidence" and "lack of integration"). The conceptualization of One Health should be more consistently defined and applied throughout the manuscript to avoid ambiguity. Minor language edits are recommended to improve flow and precision.

Regarding data presentation, clearer linkage between the data extraction framework and the thematic results would improve transparency. Tables summarizing key characteristics of included studies (e.g., mental health measures used, degree of One Health integration) could be more explicitly referenced in the main text. Figure labeling and captions could be slightly improved for clarity, particularly to guide readers in interpreting the conceptual framework. Overall, only minor editorial and presentation revisions are needed.

Many thanks for your feedback. We recognise that some of the contents is quite descriptive and there is some overlap. This was a major focus during the write up, but unfortunately due to the scoping review methodology and overlapping domains of mental health, one health and social determinants of health, we feel that no further synthesis can be made without losing clarity. We have now added the risk factors for mental health in each study to the study characteristics table to highlight the important insights (table 1).

Many thanks for the feedback regarding the figure – we have now revised it to make it clearer (line: 318)

Reviewer #2: (No Response)

Reviewer #3: Line 246: Since only a relatively small number of studies were included, it would have been helpful to have a table indicating some key parameters, such as where the study was done, the sample size, risk factors assessed and outcome measures (where relevant).

Many thanks for this feedback. We have now embedded table 1, with key study characteristics, including sample size, risk factors and outcome measures (line: 254).

Line 247: Figure 1 has asterisks (**) against ‘Records excluded’, presumably pointing to explanations of why these were excluded. However, a table footer providing these explanations is missing. Kindly add this. Because of the large number of articles excluded and the role of AI in this, reasons for exclusion are important.
Also, the Included studies box includes “Reports of included studies (n=0”. It is not clear what this means or why it is there if n=0.

Many thanks for this feedback, we have now updated the PRISMA chart to omit the content you highlighted, which was included in the original PRISMA template and is irrelevant to our study (line: 243)

**Summary and General Comments**


Use this section to provide overall comments, discuss strengths/weaknesses of the study, novelty, significance, general execution and scholarship. You may also include additional comments for the author, including concerns about dual publication, research ethics, or publication ethics. If requesting major revision, please articulate the new experiments that are needed.

Reviewer #1: This manuscript presents a timely and relevant scoping review on the intersection of mental health, social determinants, and One Health in skin neglected tropical diseases. The topic is important and aligns well with current global health priorities, particularly WHO efforts to integrate mental health and holistic approaches into NTD programmes.

A key strength is the novel interdisciplinary perspective and the attempt to synthesize evidence across multiple domains. The manuscript is well structured, and the identification of major gaps, such as the limited number of studies (n=22), lack of intervention research, and weak integration of OH approaches, is a valuable contribution.

However, some aspects could be improved. The inclusion criteria may have been overly restrictive, limiting the evidence base, and this should be more clearly justified. The use of AI-assisted tools for screening and data extraction requires clearer explanation to ensure transparency. Additionally, the analysis remains largely descriptive, and a slightly deeper synthesis would strengthen the manuscript. The concept of One Health could also be applied more consistently throughout.

Overall, this is a strong and relevant study with no major ethical concerns. With minor revisions to improve clarity, methodological transparency, and analytical depth, the manuscript would be suitable for publication.

Many thanks for this feedback, which has been addressed and detailed in the responses to your detailed comments above.

Reviewer #2: The title addresses a wide subject (NTDs, Mental Health, Social determinant, one health and integration), however, looking the content of the manuscript the current title may slightly overstate the extent to which the included studies truly applied or evaluated One Health approaches in practice and neither integration. Based on the evidence presented, the review synthesizes literature on stigma, psychosocial distress, poverty, and other social determinants linked to skin NTDs (very limited NTDs) than on integrated One Health frameworks or interventions.

Thanks for this feedback. We feel that our title ‘Mental health, social determinants and One Health in skin neglected tropical diseases (NTDs): a scoping review ‘ is sufficiently broad to describe the topic explored in our review.

Reviewer #3: The authors describe nicely how they have used AI to facilitate the review process yet have taken good precautions to verify that the findings and conclusions are indeed correct. This is very commendable.
The use of OneHealth as a co-selection lens has led to the exclusion of many articles that were as such very relevant to skin NTDs, mental health and social determinants of health. One wonders whether a review of these areas (skin NTDs, MH and SDH) and just looking for those that also included OH would have prevented this ‘bias’. Because of the overall lack specific inclusion of OH in the studies reviewed, in terms of risk factors assessed and outcomes measured, results are presented and discussed that pertain only to a relatively small selection of studies. Most of the content of the review would have benefited from a wider selection. However, this is likely to be done (or may have been done already) in another review in this series?

Thank you for this feedback. We agree that we were perhaps overly restrictive and have now reflected on this within the limitations section (line: 744-751). The unique angle of this review was focusing on the one health lens, and there are a number of existing reviews on skin NTDs, mental health and social determinants of health, as you say. Whilst we recognise that if we had used broader criteria, we may have found more papers that were theoretically aligned. However, we want to emphasise that our application of the ‘one health’ component of the search strategy was quite broad, in that the paper only had to include a search term related to either animals or the environment, and the use of the specific term ‘One Health’ was not required. We have now emphasised this within the eligibility criteria (line: 182-183).

Introduction
Line 102: “However” is not a logical term here. Perhaps ‘At the same time’?

Amended, thank you (line: 101)

Line 136: reference error

Thank you for picking this up. I can no longer see it, so assume it has corrected itself.

Line 208/9: “The NotebookLM (32) question sheet was piloted and amended, with support from Chat GPT, to ensure accuracy and quality.” I am not sure what this means, other than checking an output from one non-human source with another non-human source. I’m glad to read later on that the lead author also did manual checking and correcting of the content!

Thank you for highlighting this. Chat GPT supported us to make required amendments to the sheet, suggesting probing questions etc. We have now expanded this to be clearer (Line: 203-204).

Results

Line 269: Suggest rephrasing, since ‘Most’ is not relevant here: “Thirteen studies used quantitative and 6 used qualitative methods.”

Many thanks, this has now been amended (line: 278)

Line 286: “none of the frameworks applied a OH approach to mental illness in persons affected by skin NTDs”. It is not clear what this means, since mental illness in animals is presumably not relevant? So does it concern anxiety about animal health caused by NTDs, contributing to mental ill-health? Despite the elaboration in the subsequent paragraphs, it doesn’t become clear how the authors envisage the relationship or interaction between animal health and human mental health.

Thank you for raising this. We have now amended it to “none of the articles explicitly applied a OH framework in considering the impact of NTDs on human mental health, for example through planetary/environmental factors or the relevance of diseases having zoonotic pathway” (line: 295-297).

Line 427: I wonder if this is the right reference, since the article is about leishmaniasis, rather than leprosy?

Many thanks for spotting this error, which has now been amended, with the reference updated.

Line 466: Is 58 the wrong reference, or should the country by Tunisia, instead of Tanzania?

Again, thanks so much for spotting this error. It should have been Tunisia, and has now been corrected (line: 484)

Line 492-4: “Only two studies considered the role of existing health status. An Indonesian study found that milder LF symptoms reduced healthcare-seeking behaviour.” This doesn’t seem to be demographics? This whole section doesn’t mention OneHealth at all. The reported results are likely to be true, but it is not possible to judge the extent to which they are biased by excluding so many studies that did not include the OH concept.
The same could be said for the section on economic factors. Many studies are excluded because of the above bias. Most studies that measured social and work participation were excluded for that reason.

Thank you for highlighting this important point, which we agree with. We have now added a reflection on this source of bias within the strengths and limitations section of the discussion (line: 744-753). Lund’s social determinants of health model includes longevity and survival under demographics, so we decided that health status fit best within this section. However, in response to your comment, we have amended the sub-heading to ‘demographic and health status’ (line: 509)

Line 578: Since the results have been quite non-specific and made hardly any reference to OH, I would recommend calling these at best recommendations for ‘good practice’, since ‘best’ suggests they have been tested against or at least compared with others and found to be better.

Many thanks for this feedback, we have amended the wording as you suggest (line: 601)

Discussion
Line 589/90: it is still unclear what this phrase means: “…mental health in relation to skin NTDs from a OH perspective.” The results hardly mention OneHealth, so, while the studies may have mentioned OH, this is not really a OH perspective.

Many thanks for this feedback. In response, we have amended the phrasing too: ‘whilst we identified some literature outlining the relationship between skin NTDs and mental health that considered factors aligned with the OH perspective’ (line: 612-613)

Additionally, we have highlighted this caveat in the limitations (line: 749-751) and in the conclusion (line: ).

Line 649/50: This has already been done in the field of leprosy and LF and intervention studies have shown this to be very effective (e.g. Peters et al, 2015, 2016). Especially involvement of persons with lived experience is effective in this context (Sermrittirong et al, 2014, 2015; Lusli et al, 2015, 2016; Agarwal et al, 2023; Mol et al, 2023).

# Great. Many thanks for these additional references, which we have now incorporated (Line: 673-675)

PLOS authors have the option to publish the peer review history of their article ([what does this mean?](https://url.uk.m.mimecastprotect.com/s/W8LmCBPAmUAVLPEIWtwc2ZJgN?domain=track.editorialmanager.com)). If published, this will include your full peer review and any attached files.


If you choose “no”, your identity will remain anonymous but your review may still be made public.


**Do you want your identity to be public for this peer review?** For information about this choice, including consent withdrawal, please see our [Privacy Policy](https://url.uk.m.mimecastprotect.com/s/7sXFCDQDofMB8jghkuXcj3i-u?domain=track.editorialmanager.com).

Reviewer #1: **Yes:** Anil Fastenau

Reviewer #2: **Yes:** Wendemagegn Enbiale

Reviewer #3: **Yes:** Wim van Brakel
