# Supplementary material for: Mental health, social determinants and One Health in skin neglected tropical diseases (NTDs): A scoping review
Source: PLoS Negl Trop Dis. 2026 Aug 6;20(8):e0014034. doi: 10.1371/journal.pntd.0014034 (PMC13485127; doi:10.1371/journal.pntd.0014034)
Supplement: Peer review history

# Peer review: Decision Letter 0

**DOI:** 10.1371/journal.pntd.0014034.r001

**Authors:** Lynne Elson, Lian F. Thomas

10 Jun 2026

Response to Reviewers Revised Manuscript with Track Changes Manuscript

Shaden Kamhawi

co-Editor-in-Chief

PLOS Neglected Tropical Diseases

orcid.org/0000-0003-4304-636XX

Paul Brindley

co-Editor-in-Chief

PLOS Neglected Tropical Diseases

orcid.org/0000-0003-1765-0002

**Additional Editor Comments (if provided):** **Journal Requirements:**

1) Please upload all main figures as separate Figure files in .tif or .eps format. For more information about how to convert and format your figure files please see our guidelines:

https://journals.plos.org/plosntds/s/figures

2) Please upload a copy of Figure Fig 1 and 2 which you refer to in your text on pages 11, and 13. Or, if the figure is no longer to be included as part of the submission please remove all reference to it within the text.

3) As required by our policy on Data Availability, please ensure your manuscript or supplementary information includes the following:

- A numbered table of all studies identified in the literature search, including those that were excluded from the analyses.

- For every excluded study, the table should list the reason(s) for exclusion.

- If any of the included studies are unpublished, include a link (URL) to the primary source or detailed information about how the content can be accessed.

- A table of all data extracted from the primary research sources for the systematic review and/or meta-analysis. The table must include the following information for each study:

- Name of data extractors and date of data extraction

- Confirmation that the study was eligible to be included in the review.

- All data extracted from each study for the reported systematic review and/or meta-analysis that would be needed to replicate your analyses.

- If data or supporting information were obtained from another source (e.g. correspondence with the author of the original research article), please provide the source of data and dates on which the data/information were obtained by your research group.

- If applicable for your analysis, a table showing the completed risk of bias and quality/certainty assessments for each study or outcome.  Please ensure this is provided for each domain or parameter assessed. For example, if you used the Cochrane risk-of-bias tool for randomized trials, provide answers to each of the signalling questions for each study. If you used GRADE to assess certainty of evidence, provide judgements about each of the quality of evidence factor. This should be provided for each outcome.

- An explanation of how missing data were handled.

This information can be included in the main text, supplementary information, or relevant data repository. Please note that providing these underlying data is a requirement for publication in this journal, and if these data are not provided your manuscript might be rejected.

4) We have amended your Competing Interest statement to comply with journal style. We kindly ask that you double check the statement and let us know if anything is incorrect.

**Reviewers' comments:**

**Key Review Criteria Required for Acceptance?**

As you describe the new analyses required for acceptance, please consider the following:

**Methods**

-Are the objectives of the study clearly articulated with a clear testable hypothesis stated?

-Is the study design appropriate to address the stated objectives?

-Is the population clearly described and appropriate for the hypothesis being tested?

-Is the sample size sufficient to ensure adequate power to address the hypothesis being tested?

-Were correct statistical analysis used to support conclusions?

-Are there concerns about ethical or regulatory requirements being met?

Reviewer #1: The objectives of the study are clearly articulated and appropriate for a scoping review, aiming to map evidence and identify gaps at the intersection of mental health, social determinants, and One Health in skin NTDs. While no explicit testable hypothesis is stated, this is acceptable given the exploratory nature of the study. The scoping review design is suitable and aligns with the stated aims; however, the requirement that studies address all four domains may have been overly restrictive and limited the number of included studies (n=22).

The population is clearly described and appropriate. As this is a scoping review, issues of sample size and statistical power are not directly applicable, though the limited number of included studies somewhat constrains generalizability. The use of descriptive thematic analysis is appropriate, but the synthesis remains largely descriptive and could benefit from deeper analytical integration.

There are no major ethical concerns, as the study is based on published literature. However, the use of AI tools for screening and data extraction requires clearer reporting to ensure transparency and reproducibility. Overall, the methods are appropriate, with minor clarifications needed.

Reviewer #2: The study objectives are clearly stated and the use of a scoping review design is appropriate for exploring the emerging intersection of mental health, social determinants, One Health (OH), and skin NTDs. The search strategy and eligibility criteria are generally well described and transparently reported. However, the review includes a relatively small and highly heterogeneous body of literature, with most studies focusing on single diseases and few explicitly applying integrated OH frameworks. In addition, many included studies did not directly measure mental health outcomes using validated tools, limiting the strength of some conceptual conclusions. While the descriptive thematic synthesis is suitable for a scoping review, the absence of formal quality appraisal or risk-of-bias assessment makes it difficult to judge the robustness of the evidence. The manuscript is commendably transparent regarding the use of AI-assisted tools for screening and data extraction, although concerns related to reproducibility, extraction bias, and consistency of interpretation remain and should be more explicitly acknowledged when interpreting the findings.

Reviewer #3: Yes overall. This concerns a scoping review, so sample size and statistical analysis are not relevant.

No concerns regarding methodology and ethics.

**********

**Results**

-Does the analysis presented match the analysis plan?

-Are the results clearly and completely presented?

-Are the figures (Tables, Images) of sufficient quality for clarity?

Reviewer #1: The analysis presented is generally consistent with the stated analysis plan. The authors indicate a descriptive thematic analysis approach, and the results reflect this, with findings structured across key domains such as mental health, One Health dimensions, and social determinants. However, the analysis remains largely descriptive, and a clearer linkage between the extraction framework and the presented themes would improve transparency.

The results are clearly presented and well-organized, with logical subheadings and a coherent narrative. Key findings, such as the limited number of studies (n=22), the dominance of quantitative designs, and the lack of integrated One Health approaches, are communicated effectively. That said, some sections are repetitive, and the presentation could benefit from more concise synthesis and clearer highlighting of the most important insights.

The figures and tables are generally of good quality and support understanding. The PRISMA flow diagram (Figure 1) is clear and appropriate, and the conceptual framework (Figure 2) adds value by illustrating the relationships between mental health, social determinants, and One Health dimensions. Minor improvements in figure labeling and integration into the text could further enhance clarity.

Reviewer #2: The findings are presented clearly overall, and the authors appropriately acknowledge the limited and heterogeneous nature of the evidence base. However, some sections move beyond descriptive synthesis into broader conceptual interpretation, particularly regarding OH integration, despite the relatively small number of studies explicitly addressing OH approaches. The presentation would also benefit from clearer differentiation between directly measured findings and interpretive inferences made by the authors. The PRISMA flow diagram and conceptual framework figure are useful and generally of adequate quality, although Figure 2 is conceptually dense and may benefit from simplification or clearer labeling to improve readability. In addition, summary tables synthesizing key study characteristics, mental health outcomes, and OH dimensions in a more concise comparative format would strengthen clarity and accessibility of the results section.

Reviewer #3: Line 246: Since only a relatively small number of studies were included, it would have been helpful to have a table indicating some key parameters, such as where the study was done, the sample size, risk factors assessed and outcome measures (where relevant).

Line 247: Figure 1 has asterisks (**) against ‘Records excluded’, presumably pointing to explanations of why these were excluded. However, a table footer providing these explanations is missing. Kindly add this. Because of the large number of articles excluded and the role of AI in this, reasons for exclusion are important.

Also, the Included studies box includes “Reports of included studies (n=0”. It is not clear what this means or why it is there if n=0.

Line 269: Suggest rephrasing, since ‘Most’ is not relevant here: “Thirteen studies used quantitative and 6 used qualitative methods.”

Line 286: “none of the frameworks applied a OH approach to mental illness in persons affected by skin NTDs”. It is not clear what this means, since mental illness in animals is presumably not relevant? So does it concern anxiety about animal health caused by NTDs, contributing to mental ill-health? Despite the elaboration in the subsequent paragraphs, it doesn’t become clear how the authors envisage the relationship or interaction between animal health and human mental health.

Line 427: I wonder if this is the right reference, since the article is about leishmaniasis, rather than leprosy?

Line 466: Is 58 the wrong reference, or should the country by Tunisia, instead of Tanzania?

Line 492-4: “Only two studies considered the role of existing health status. An Indonesian study found that milder LF symptoms reduced healthcare-seeking behaviour.” This doesn’t seem to be demographics? This whole section doesn’t mention OneHealth at all. The reported results are likely to be true, but it is not possible to judge the extent to which they are biased by excluding so many studies that did not include the OH concept.

The same could be said for the section on economic factors. Many studies are excluded because of the above bias. Most studies that measured social and work participation were excluded for that reason.

Line 578: Since the results have been quite non-specific and made hardly any reference to OH, I would recommend calling these at best recommendations for ‘good practice’, since ‘best’ suggests they have been tested against or at least compared with others and found to be better.

**********

**Conclusions**

-Are the conclusions supported by the data presented?

-Are the limitations of analysis clearly described?

-Do the authors discuss how these data can be helpful to advance our understanding of the topic under study?

-Is public health relevance addressed?

Reviewer #1: The conclusions are supported by the data presented, appropriately highlighting the limited evidence base and lack of integrated One Health approaches without overinterpretation.

The limitations are clearly described, including the small number of included studies, heterogeneity, and methodological constraints such as the use of AI tools and exclusion of grey literature.

The authors adequately discuss how the findings advance understanding by identifying key gaps and priorities for future research, particularly around integration of mental health and One Health. Public health relevance is clearly addressed, with strong links to global health priorities and implications for policy and health systems.

Reviewer #2: The conclusions are generally consistent with the overall findings of the review and appropriately emphasize the limited and fragmented evidence base linking mental health, social determinants, and One Health approaches in skin NTDs. However, some conclusions regarding integrated OH mechanisms and frameworks appear stronger than the available evidence can fully support, given that only a small number of studies explicitly operationalized OH approaches. The limitations of the review are transparently acknowledged, including the heterogeneity of included studies, limited intervention evidence, exclusion of grey literature, and challenges related to AI-assisted screening and extraction. The discussion appropriately highlights important research and policy implications, particularly the need for participatory, interdisciplinary, and person centred approaches. However, the manuscript could further strengthen the conclusions by more cautiously distinguishing between evidence directly supported by the included studies and broader conceptual or theoretical interpretations proposed by the authors.

Reviewer #3: Yes, with the limitation that OneHealth was little mentioned, even though the studies met the inclusion criteria.

Line 674-6: “OH plays a key role in shaping experiences of mental health in persons affected by skin NTDs, largely through SDH, such as changing environment leading to increased risk to traditional livelihoods.” It should be noted that this is still a hypothesis, since the current review did not find clear evidence to support this.

Limitations:

Line 694: the above-mentioned bias in the article selection because of using OH as a co-selection criterion is not mentioned. Many studies on Skin NTDs, stigma, mental health, social participation and even economic aspects were not included because of this.

**********

**Editorial and Data Presentation Modifications?**

Use this section for editorial suggestions as well as relatively minor modifications of existing data that would enhance clarity. If the only modifications needed are minor and/or editorial, you may wish to recommend “Minor Revision” or “Accept”.

Reviewer #1: The manuscript is generally well written and clearly structured, but a few editorial and presentation improvements would enhance clarity and readability. Some sections, particularly the Results and Discussion, are repetitive and could be streamlined to reduce redundancy (e.g., repeated emphasis on "limited evidence"and "lack of integration"). The conceptualization of One Health should be more consistently defined and applied throughout the manuscript to avoid ambiguity. Minor language edits are recommended to improve flow and precision.

Regarding data presentation, clearer linkage between the data extraction framework and the thematic results would improve transparency. Tables summarizing key characteristics of included studies (e.g., mental health measures used, degree of One Health integration) could be more explicitly referenced in the main text. Figure labeling and captions could be slightly improved for clarity, particularly to guide readers in interpreting the conceptual framework. Overall, only minor editorial and presentation revisions are needed.

Reviewer #2: (No Response)

Reviewer #3: Line 246: Since only a relatively small number of studies were included, it would have been helpful to have a table indicating some key parameters, such as where the study was done, the sample size, risk factors assessed and outcome measures (where relevant).

Line 247: Figure 1 has asterisks (**) against ‘Records excluded’, presumably pointing to explanations of why these were excluded. However, a table footer providing these explanations is missing. Kindly add this. Because of the large number of articles excluded and the role of AI in this, reasons for exclusion are important.

Also, the Included studies box includes “Reports of included studies (n=0”. It is not clear what this means or why it is there if n=0.

**********

**Summary and General Comments**

Use this section to provide overall comments, discuss strengths/weaknesses of the study, novelty, significance, general execution and scholarship. You may also include additional comments for the author, including concerns about dual publication, research ethics, or publication ethics. If requesting major revision, please articulate the new experiments that are needed.

Reviewer #1: This manuscript presents a timely and relevant scoping review on the intersection of mental health, social determinants, and One Health in skin neglected tropical diseases. The topic is important and aligns well with current global health priorities, particularly WHO efforts to integrate mental health and holistic approaches into NTD programmes.

A key strength is the novel interdisciplinary perspective and the attempt to synthesize evidence across multiple domains. The manuscript is well structured, and the identification of major gaps, such as the limited number of studies (n=22), lack of intervention research, and weak integration of OH approaches, is a valuable contribution.

However, some aspects could be improved. The inclusion criteria may have been overly restrictive, limiting the evidence base, and this should be more clearly justified. The use of AI-assisted tools for screening and data extraction requires clearer explanation to ensure transparency. Additionally, the analysis remains largely descriptive, and a slightly deeper synthesis would strengthen the manuscript. The concept of One Health could also be applied more consistently throughout.

Overall, this is a strong and relevant study with no major ethical concerns. With minor revisions to improve clarity, methodological transparency, and analytical depth, the manuscript would be suitable for publication.

Reviewer #2: The title addresses a wide subject (NTDs, Mental Health, Social determinant, one health and integration), however, looking the content of the manuscript the current title may slightly overstate the extent to which the included studies truly applied or evaluated One Health approaches in practice and neither integration. Based on the evidence presented, the review synthesizes literature on stigma, psychosocial distress, poverty, and other social determinants linked to skin NTDs (very limited NTDs) than on integrated One Health frameworks or interventions.

Reviewer #3: The authors describe nicely how they have used AI to facilitate the review process yet have taken good precautions to verify that the findings and conclusions are indeed correct. This is very commendable.

The use of OneHealth as a co-selection lens has led to the exclusion of many articles that were as such very relevant to skin NTDs, mental health and social determinants of health. One wonders whether a review of these areas (skin NTDs, MH and SDH) and just looking for those that also included OH would have prevented this ‘bias’. Because of the overall lack specific inclusion of OH in the studies reviewed, in terms of risk factors assessed and outcomes measured, results are presented and discussed that pertain only to a relatively small selection of studies. Most of the content of the review would have benefited from a wider selection. However, this is likely to be done (or may have been done already) in another review in this series?

Introduction

Line 102: “However” is not a logical term here. Perhaps ‘At the same time’?

Line 136: reference error

Line 208/9: “The NotebookLM (32) question sheet was piloted and amended, with support from Chat GPT, to ensure accuracy and quality.” I am not sure what this means, other than checking an output from one non-human source with another non-human source. I’m glad to read later on that the lead author also did manual checking and correcting of the content!

Results

Line 269: Suggest rephrasing, since ‘Most’ is not relevant here: “Thirteen studies used quantitative and 6 used qualitative methods.”

Line 286: “none of the frameworks applied a OH approach to mental illness in persons affected by skin NTDs”. It is not clear what this means, since mental illness in animals is presumably not relevant? So does it concern anxiety about animal health caused by NTDs, contributing to mental ill-health? Despite the elaboration in the subsequent paragraphs, it doesn’t become clear how the authors envisage the relationship or interaction between animal health and human mental health.

Line 427: I wonder if this is the right reference, since the article is about leishmaniasis, rather than leprosy?

Line 466: Is 58 the wrong reference, or should the country by Tunisia, instead of Tanzania?

Line 492-4: “Only two studies considered the role of existing health status. An Indonesian study found that milder LF symptoms reduced healthcare-seeking behaviour.” This doesn’t seem to be demographics? This whole section doesn’t mention OneHealth at all. The reported results are likely to be true, but it is not possible to judge the extent to which they are biased by excluding so many studies that did not include the OH concept.

The same could be said for the section on economic factors. Many studies are excluded because of the above bias. Most studies that measured social and work participation were excluded for that reason.

Line 578: Since the results have been quite non-specific and made hardly any reference to OH, I would recommend calling these at best recommendations for ‘good practice’, since ‘best’ suggests they have been tested against or at least compared with others and found to be better.

Discussion

Line 589/90: it is still unclear what this phrase means: “…mental health in relation to skin NTDs from a OH perspective.” The results hardly mention OneHealth, so, while the studies may have mentioned OH, this is not really a OH perspective.

Line 649/50: This has already been done in the field of leprosy and LF and intervention studies have shown this to be very effective (e.g. Peters et al, 2015, 2016). Especially involvement of persons with lived experience is effective in this context (Sermrittirong et al, 2014, 2015; Lusli et al, 2015, 2016; Agarwal et al, 2023; Mol et al, 2023).

**********

PLOS authors have the option to publish the peer review history of their article (what does this mean?). If published, this will include your full peer review and any attached files.

If you choose “no”, your identity will remain anonymous but your review may still be made public.

**Do you want your identity to be public for this peer review?**  For information about this choice, including consent withdrawal, please see our Privacy Policy.

Reviewer #1: **Yes:**  Anil Fastenau

Reviewer #2: **Yes:**  Wendemagegn Enbiale

Reviewer #3: **Yes:**  Wim van Brakel

**Figure resubmission:**

**Reproducibility:**

To enhance the reproducibility of your results, we recommend that authors of applicable studies deposit laboratory protocols in protocols.io, where a protocol can be assigned its own identifier (DOI) such that it can be cited independently in the future. Additionally, PLOS ONE offers an option to publish peer-reviewed clinical study protocols. Read more information on sharing protocols at https://plos.org/protocols?utm_medium=editorial-email&utm_source=authorletters&utm_campaign=protocols

# Author comment: Author response to Decision Letter 1

**DOI:** 10.1371/journal.pntd.0014034.r002

1 Jul 2026

Attachment Submitted filename: Response to Reviewers.docx

# Editor report: Decision Letter 1

**DOI:** 10.1371/journal.pntd.0014034.r003

**Authors:** Lynne Elson, Lian F. Thomas

6 Jul 2026

Dear Dr. Hotopf,

We are pleased to inform you that your manuscript 'Mental health, social determinants and One Health in skin neglected tropical diseases (NTDs): a scoping review' has been provisionally accepted for publication in PLOS Neglected Tropical Diseases.

Before your manuscript can be formally accepted you will need to complete some formatting changes, which you will receive in a follow up email. A member of our team will be in touch with a set of requests.

Please note that your manuscript will not be scheduled for publication until you have made the required changes, so a swift response is appreciated.

IMPORTANT: The editorial review process is now complete. PLOS will only permit corrections to spelling, formatting or significant scientific errors from this point onwards. Requests for major changes, or any which affect the scientific understanding of your work, will cause delays to the publication date of your manuscript.

Should you, your institution's press office or the journal office choose to press release your paper, you will automatically be opted out of early publication. We ask that you notify us now if you or your institution is planning to press release the article. All press must be co-ordinated with PLOS.

Thank you again for supporting Open Access publishing; we are looking forward to publishing your work in PLOS Neglected Tropical Diseases.

Best regards,

Lynne Elson, PhD, MPH

Guest Editor

PLOS Neglected Tropical Diseases

Song Liang

Section Editor

PLOS Neglected Tropical Diseases

Shaden Kamhawi

co-Editor-in-Chief

PLOS Neglected Tropical Diseases

orcid.org/0000-0003-4304-636XX

Paul Brindley

co-Editor-in-Chief

PLOS Neglected Tropical Diseases

orcid.org/0000-0003-1765-0002

***********************************************************

This manuscript required only minor revisions. In my opinion the authors have addressed all of the reviewers' comments appropriately. Therefore I consider there is no need for further review and the manuscript is ready for acceptance.

There is a need for careful copy editing before publication. For instance the Supporting Information files 4 and 5 are sometimes referred to as SL4 and SL5 and other times as SI4 or SI5. In addition, I hope in the final published manuscript Table 1 can be printed in a 'landscape' format for ease of reading.

I notice an extra authors' affiliation has been added. Please check this has been included in the author section of the Editorial Manager.

p.p1 {margin: 0.0px 0.0px 0.0px 0.0px; line-height: 16.0px; font: 14.0px Arial; color: #323333; -webkit-text-stroke: #323333}span.s1 {font-kerning: none

# Editor report: Acceptance letter

**DOI:** 10.1371/journal.pntd.0014034.r004

**Authors:** Lynne Elson, Lian F. Thomas

Dear Hotopf,

We are delighted to inform you that your manuscript, "Mental health, social determinants and One Health in skin neglected tropical diseases (NTDs): a scoping review," has been formally accepted for publication in PLOS Neglected Tropical Diseases.

We have now passed your article onto the PLOS Production Department who will complete the rest of the publication process. All authors will receive a confirmation email upon publication.

The corresponding author will soon be receiving a typeset proof for review, to ensure errors have not been introduced during production. Please review the PDF proof of your manuscript carefully, as this is the last chance to correct any scientific or type-setting errors. Please note that major changes, or those which affect the scientific understanding of the work, will likely cause delays to the publication date of your manuscript. Note: Proofs for Front Matter articles (Editorial, Viewpoint, Symposium, Review, etc...) are generated on a different schedule and may not be made available as quickly.

Soon after your final files are uploaded, the early version of your manuscript will be published online unless you opted out of this process. The date of the early version will be your article's publication date. The final article will be published to the same URL, and all versions of the paper will be accessible to readers.

For Research Articles, you will receive an invoice from PLOS for your publication fee after your manuscript has reached the completed accept phase. If you receive an email requesting payment before acceptance or for any other service, this may be a phishing scheme. Learn how to identify phishing emails and protect your accounts at https://explore.plos.org/phishing.

Thank you again for supporting open-access publishing; we are looking forward to publishing your work in PLOS Neglected Tropical Diseases.

Best regards,

Shaden Kamhawi

co-Editor-in-Chief

PLOS Neglected Tropical Diseases

Paul Brindley

co-Editor-in-Chief

PLOS Neglected Tropical Diseases
